# Distinct genomic signals of lifespan and life history evolution in response to postponed reproduction and larval diet in *Drosophila*

**DOI:** 10.1002/evl3.143

**Published:** 2019-10-02

**Authors:** Katja M. Hoedjes, Joost van den Heuvel, Martin Kapun, Laurent Keller, Thomas Flatt, Bas J. Zwaan

**Affiliations:** ^1^ Department of Ecology and Evolution University of Lausanne Lausanne Switzerland; ^2^ Laboratory of Genetics, Plant Sciences Group Wageningen University Wageningen The Netherlands; ^3^ Institute for Cell and Molecular Biosciences Newcastle University Newcastle Upon Tyne United Kingdom; ^4^ Department of Biology University of Fribourg Fribourg Switzerland; ^5^ Current Address: Department of Evolutionary Biology and Environmental Studies University of Zurich Zurich Switzerland

**Keywords:** Adaptation, diet, “evolve and resequence”, longevity, reproduction

## Abstract

Reproduction and diet are two major factors controlling the physiology of aging and life history, but how they interact to affect the evolution of longevity is unknown. Moreover, although studies of large‐effect mutants suggest an important role of nutrient sensing pathways in regulating aging, the genetic basis of evolutionary changes in lifespan remains poorly understood. To address these questions, we analyzed the genomes of experimentally evolved *Drosophila melanogaster* populations subjected to a factorial combination of two selection regimes: reproductive age (early versus postponed), and diet during the larval stage (“low,” “control,” “high”), resulting in six treatment combinations with four replicate populations each. Selection on reproductive age consistently affected lifespan, with flies from the postponed reproduction regime having evolved a longer lifespan. In contrast, larval diet affected lifespan only in early‐reproducing populations: flies adapted to the “low” diet lived longer than those adapted to control diet. Here, we find genomic evidence for strong independent evolutionary responses to either selection regime, as well as loci that diverged in response to both regimes, thus representing genomic interactions between the two. Overall, we find that the genomic basis of longevity is largely independent of dietary adaptation. Differentiated loci were not enriched for “canonical” longevity genes, suggesting that naturally occurring genic targets of selection for longevity differ qualitatively from variants found in mutant screens. Comparing our candidate loci to those from other “evolve and resequence” studies of longevity demonstrated significant overlap among independent experiments. This suggests that the evolution of longevity, despite its presumed complex and polygenic nature, might be to some extent convergent and predictable.

**Impact Summary**

Both reproduction and diet have a major impact on aging and lifespan, but how these two factors interact to shape the evolution of longevity remains unknown. We have studied the genomes of 24 experimentally evolved fruit fly lines that have adapted their life history, most notably lifespan, in response to selection for postponed reproduction and/or coping with over‐ or undernutrition during the larval stage. Selection on postponed reproduction resulted in a strong and consistent lifespan extension, whereas an effect of larval diet on longevity was observed in early‐reproducing populations only; flies adapted to the poorest diet lived longer than those kept on the control diet. Our genome analyses mirror these findings, with strong, independent responses to the two selective regimes, as well as loci that diverged in response to both regimes, thus indicating genomic interactions. Overall, the evolution of lifespan in response of postponed reproduction is mostly independent of larval diet. Moreover, qualitatively different loci underlie the response in lifespan and life history in early‐reproducing populations adapted to the poor diet. All of our identified candidate loci have little overlap with known “aging” genes, suggesting that naturally occurring variants involved in longevity evolution are distinct from variants identified through classical mutant screens. On the other hand, there is a significant overlap of our candidates with those identified in other independent longevity “evolve and resequence” studies, which may indicate the presence of preferred targets of selection for the evolution of lifespan. In conclusion, our approach provides a powerful method for discovering novel loci involved in the evolution of lifespan and life histories. The application of two selective regimes can help to disentangle genomic differentiation related to different modes of lifespan evolution, and to other life history phenotypes that evolve in concert.

Reproduction and nutrition are major determinants of lifespan, both physiologically and evolutionarily. At the physiological level, reduced reproduction extends lifespan (Maynard Smith 1958; Hsin and Kenyon 1999; Flatt et al. 2008; Flatt 2011). Also, dietary manipulation either during development or adulthood, for instance dietary restriction (DR; reduced food intake without malnutrition), often affects lifespan and fecundity antagonistically (e.g., Economos and Lints 1984; Chippindale et al. 1993; Mair et al. 2005; Mair and Dillin 2008; Tatar 2011; May et al. 2015; Stefana et al. 2017). Levels of dietary intake ultimately influence decisions about how resources are allocated to the competing demands of reproduction versus somatic maintenance (e.g., Kirkwood 1977; Van Noordwijk and De Jong 1986; Kirkwood 1990; De Jong and Van Noordwijk 1992). Mechanistically, these processes may be controlled by modulating the activity of interacting nutrient sensing pathways, such as the insulin/insulin‐like growth factor signaling (IIS) and target of rapamycin (TOR) pathways (e.g., Flatt et al. 2008; Grandison et al. 2009). Indeed, these signaling networks play evolutionarily conserved roles in regulating life history physiology in both invertebrates and vertebrates: mutations in these networks often have major effects on lifespan, so that their components are thought to represent “canonical” large‐effect loci underlying longevity (e.g., Kenyon 2001; Partridge and Gems 2002; Tatar et al. 2003). However, recent studies have found little evidence that these “canonical” loci are the target of selection for lifespan in natural populations (Remolina et al. 2012; Fabian et al. 2018; Flatt and Partridge 2018).

At the evolutionary level, late‐life fertility and longevity can be selected experimentally by postponing reproduction to later adult ages, typically at the expense of reduced early fecundity (Luckinbill et al. 1984; Rose 1984; Partridge et al. 1999, but see Nusbaum and Rose 1999). Such selection experiments, mainly performed in the fruit fly *Drosophila melanogaster*, demonstrate the existence of genetically determined life history trade‐offs, presumably due to pleiotropic alleles with antagonistic effects upon lifespan and reproduction (e.g., Williams 1957; Flatt 2011). Adaptation to different dietary conditions can also have profound effects on life history evolution. In particular, selection for increased survival upon adult starvation often leads to lifespan extension and reduced fecundity as a correlated response (reviewed in Hoffmann and Harshman 1999; Rion and Kawecki 2007). Yet, how adaptation to dietary conditions during development influences adult life history evolution is less well understood; a study by Kolss et al. (2009) found that adaptation to chronic larval malnutrition could constrain the evolution of adult *Drosophila* life history traits. Given that both diet and reproduction affect longevity physiologically, how do they interact evolutionarily? Does adaptation to nutritional resources during development prevent or modify the evolution of longevity and correlated life history traits in response to delayed reproduction? And if so, which are the genetic loci through which the two regimes interact?

To address these questions, we studied a set of 24 experimental evolution (EE) populations of *D. melanogaster* that diverged in lifespan and life history in response to a factorial combination of two selection regimes: developmental diet (“low” [L], “control” [C], or “high” [H] diet) and reproductive age (“early” [E] vs. “postponed” [P] reproduction), that is, six regime combinations with four replicate populations each (May et al. 2019; Fig. 1). In this experiment, selection for postponed reproduction led to the evolution of lifespan extension (up to ∼25%) across all late‐reproducing populations and diets, as well as an increase in adult size and late‐life fecundity. Adaptation to developmental diet alone did not consistently affect lifespan, but it led to a clear evolutionary divergence in development time and adult weight (both decreased), in particular in response to the “low” diet. These observations suggest that the two selective regimes could act relatively independent of each other (May et al. 2019). However, the magnitude of the lifespan and life history responses to each selective regime did depend on the other regime. For example, early‐reproducing flies selected under “low” diet lived longer than early‐reproducing flies kept under a control diet. Also the decrease in development time and adult weight on the “low” diet was most pronounced for early reproducing populations (May et al. 2019). This indicated that the two selection regimes also interacted in affecting the evolution of lifespan and correlated traits.

**Figure 1:**
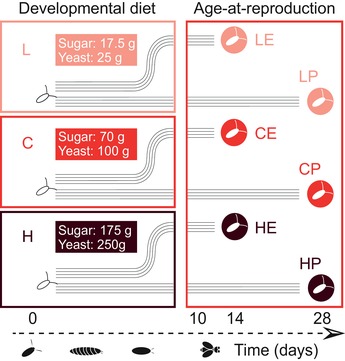
Overview of the experimental evolution (EE) experiment. Two selection regimes, that is, adaptation to developmental diet (“low” [L], “control” [C], or “high” [H] diet) and selection on age at reproduction (“early” [E] versus “postponed” [P] reproduction), have been combined in a fully factorial fashion. The flies were kept on one of the three diets, which differ in the amount of sugar and yeast per liter medium as indicated, throughout their development, whereas adults were all kept on control diet.

Here, we used whole‐genome pool‐sequencing (Pool‐seq) to examine the genetic basis underlying the evolution of lifespan and life history in response to joint selection on reproductive age and adaptation to developmental nutrition, and to investigate whether and how the two influence each other at the genomic level.

## Methods

### EXPERIMENTAL DESIGN AND SAMPLE PREPARATION

We studied the genomes of 24 experimentally evolved (EE) populations of *D. melanogaster* subjected to a fully factorial combination of two selection pressures: larval diet (with three levels differing in their concentration of nutrients: “low” [L], “control” [C], or “high” [H] diet) and age at reproduction (with two levels: “early” [E] vs. “postponed” [P] reproduction), with four independent replicate lines per regime combination (see Fig. 1). The larval diets differ in the amount of sugar and yeast they contain, with the “L” and the “H” diet containing 0.25× and 2.5× nutrients, respectively, compared to the “C” diet. The generation times of the “E” and “L” populations were 14 and 28 days, which means that adults laid eggs for the subsequent generation at approximately 2–4 or 16–18 days after eclosion, respectively. The lines were kept at 25°C, 65% humidity and 12‐h:12‐h light:dark cycle. The setup of the EE study is described in detail in May et al. (2019). In short, to maximize the amount of standing genetic variation on which selection could act, the EE base population was generated by combining flies from six populations collected across Europe that had been maintained in the laboratory for 40 generations (May et al. 2015). After crossing, the founding population was maintained for another 10 generations at a population size of ∼4000 individuals before onset of EE. The population size of each of the selected lines was about 2000–4000 individuals over the course of EE. Life history phenotypes (lifespan, development time, fecundity, and adult weight) were measured after multiple intervals of selection (May et al. 2019).

Pooled gDNA samples from 250 female flies were prepared for sequencing on the Illumia HiSeq 2500 platform (Pool‐seq) for each of the 24 EE populations (see Supporting Information Methods for the full protocol). Flies were sampled at generation 115 (E lines) or 58 (P lines). The genomes of were sequenced to an average coverage of 79–109× per population (Supporting Information Result S1).

### GENOME ANALYSIS

All information on the analysis of the genome data are given in the Supporting Information Methods.

## Results and Discussion

### DIET AND POSTPONED REPRODUCTION PRODUCE GENOME‐WIDE SIGNATURES OF SELECTION

**Figure 2:**
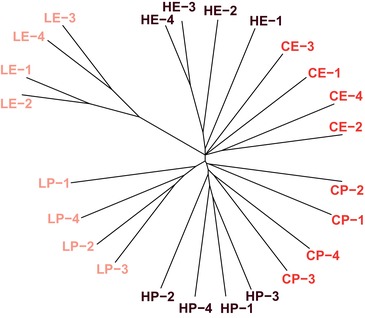
Genetic differentiation among the EE populations. To visualize the overall genetic diversity among the 24 EE populations, we constructed a clustering tree by analyzing pairwise differences among the populations based on 6500 randomly drawn SNPs (1000× bootstrapped). All postponed reproduction (“P”) populations cluster together, as well as the early reproducing (“E”) populations, but a weaker clustering based on diet is visible as well, especially for the “L” diet. The four low‐early (“LE”) populations had differentiated most strongly from all the other 20 EE populations.

To analyze the genomic signatures of larval dietary adaptation, the evolution of adult lifespan (in response to selection for postponed reproduction) and their interaction, we obtained genome‐wide allele frequency estimates from Pool‐seq (Schlotterer et al. 2015) of all 24 EE populations. A clustering tree (Fig. 2), constructed by analyzing pairwise differences of randomly drawn SNPs among the populations, provided a first overview of the genetic differentiation between the selective regimes: (1) the postponed reproduction (“P”) populations, as well as the early reproducing (“E”) populations, cluster together; (2) a weaker clustering based on diet is visible as well, especially for the low nutrient (“L”) diet; and (3) the four low‐early (“LE”) populations had genetically diverged most strongly from all other 20 EE populations. This indicates the existence of both “private” (i.e., specific to a single regime) responses to the two selection regimes as well as interactions between them. Generally speaking, the four replicate lines per regime combination cluster together, indicating parallel responses to selection.

We then analyzed our dataset to identify loci with consistent allele frequency differences among the selective regimes, hereby focusing on responses that are shared among the four replicate lines per regime combination as a robust measure of parallel evolution. An important consideration when aiming to detect such differences between selection regimes in “evolve and resequence” (E&R) studies is the choice of the proper statistical analysis framework (Wiberg et al. 2017). As there is no precedent for analyzing the genomic interaction of EE regimes, we examined the performance of four different types of statistical models using simulated datasets. These datasets were created using different assumptions regarding selection intensity, population size, and initial allele frequencies to simulate Pool‐seq data from evolving populations with characteristics expected to match our dataset. These simulated datasets were analyzed using the following statistical models: analysis of variance on arcsine square root transformed allele frequencies (Kelly et al. 2013), GLM with binomial error structure on the read counts (Martins et al. 2014), GLM with a quasibinomial error structure (Wiberg et al. 2017), and a generalized linear mixed model (GLMM) with binomial error structure and replicate population as a random effect (Jha et al. 2015). The binomial GLM model had a superior performance compared to the three other models in terms of detecting allele frequency differentiation and true discovery rate for both main effects and, importantly, the interaction. Therefore, we considered the binomial GLM model the most suitable method for identifying SNP allele differentiation in our dataset (details of these analyses and their results are given in Supporting Information Result S2). This model however does not account for overdispersion (Lynch et al. 2014; Wiberg et al. 2017; Kelly and Hughes 2019), which may result in unrealistically low *P*‐values (Supporting Information Result S3). To correct for this, we calculated *P*‐values of permuted datasets to generate an empirical null distribution (equally overdispersed) that was subsequently used to estimate FDR of the observed data, following the procedure by Benjamini and Hochberg (1995) (Jha et al. 2015).

**Figure 3:**
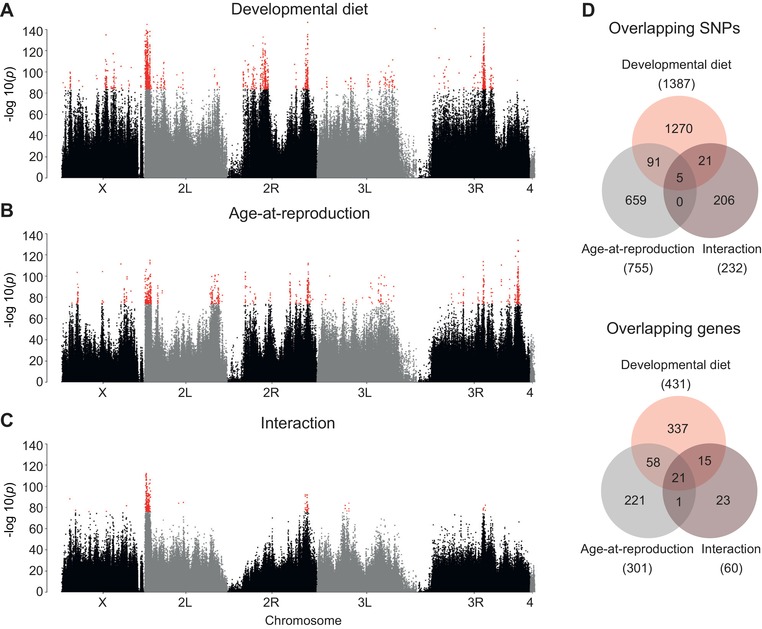
Genome‐wide patterns of SNP allele differentiation as identified with GLM. (A–C) Manhattan plots indicate regions of SNP allele frequency differentiation across the genome for the two main factors, “developmental diet” and “age at reproduction” and their interaction. Significantly differentiated SNPs with a FDR = 0 are indicated in red. (D) Overlaps between the “main effects” of the two selection regimes and the interaction between them, both for significantly differentiated SNPs and genes.

By applying the binomial GLM model to our Pool‐seq data, we identified a total of 2252 significantly diverged SNPs (Fig. 3A–D, Table S1, Supporting Information Result S4). This set of significantly diverged SNPs consists of both loci under selection, as well as hitchhiking loci that are genetically linked to them. Of these, 1387 SNPs in 431 genes were significantly affected by diet (i.e., that show differentiation among the low (L), control (C), and/or high (H) diets), and 755 SNPs in 301 genes exhibiting significant allelic divergence between the early (E) and postponed (P) populations (Fig. 3A,B). Both selection regimes led to highly localized, sharp peaks of genomic differentiation, especially on chromosome arms 2L, 2R, and 3R, indicating strong polygenic responses to selection (Fig. 3A,B). Several genomic regions in both regimes showed particularly pronounced divergence, consistent with strong but partial, soft sweeps (maximum allele frequency differentiation between *E* and *P* = 0.48 and between *L*, *H*, and *C* = 0.56; see Supporting Information Result S4). Although some peaks of divergence overlapped between the two selection regimes; in particular on 2*L* and 2*R*, only 96 SNPs in 79 genes were shared between them (Fig. 3D). We found a relatively small number of “interaction” loci (232 SNPs, 60 genes), that is, loci that responded to a combination of selection for diet adaptation (L, C, or H) and longevity (E or P) (Fig. 3C,D). The identified “interaction” loci were rather specific, with the majority of them (206 out of 232 SNPs, 88%) not overlapping with any candidates from either regime. However, the interaction of the two regimes may be underestimated by the fact that linear models have less power to detect interactions as compared to main effects (see Supporting Information Result S2).

Indeed, upon inspection of the candidates, the GLM appeared underpowered in terms of its ability to distinguish interaction effects from main effects for numerous loci. Given that both the extent and the type of interaction between the selection regimes did not become sufficiently clear from the GLM analysis, we performed a cluster analysis of the complete set of *all* 2252 diverged SNPs. As this analysis clusters together loci with similar allele frequency differentiation patterns, it provided a more accurate method to detect interactions between the two regimes and it enabled us to characterize specific patterns of allelic differentiation of interaction loci, and thus insights in the nature of the interaction (Supporting Information Result S5).

### LIFESPAN EVOLUTION IN RESPONSE TO POSTPONED REPRODUCTION IS MOSTLY INDEPENDENT OF DIET ADAPTATION

**Figure 4:**
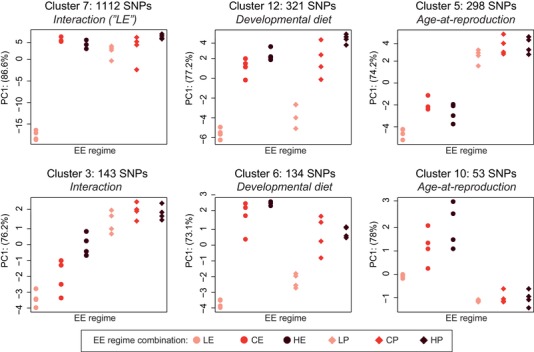
Distinct patterns of allele frequency differentiation reveal responses to the two selective regimes. A cluster analysis groups together SNPs with similar allele differentiation patterns, allowing us to characterize main effects of the two selective regimes and different types of interaction effects. The total number of clusters was 25, ranging in size from 1 to 1112 SNPs. The six largest clusters of SNPs are shown (91.5% of all significant SNPs). For each cluster, PCA was used to extract the eigenvector (PC1, representing 75–85% of total variance in the cluster) of the alleles frequencies; the location of the 24 populations (*x*‐axis) on PC1 (*y*‐axis) is indicated, which is a qualitative representation of the average relative allele frequency pattern among the 24 EE populations for the SNPs in the cluster. Populations with high frequencies of the major allele have a high value on PC1, whereas low frequencies of the major allele are indicated by lower PC1 values. Based on these values, we determined how SNPs in each cluster had responded to the two EE regimes (see Supporting Information Result S5 for methodology and results). Clusters 7 and 3 are examples of SNPs for which the two selection regimes interact, whereas clusters 5 and 10, and clusters 6 and 12 include SNPs that are “private” to the “age at reproduction” and “developmental diet” regimes, respectively.

The cluster analysis revealed that 39% of the diverged SNPs were “private” to one of the regimes (Fig. 4, Supporting Information Result S5, Table S1): 399 SNPs in 154 genes diverged specifically in response to selection for reproductive age (hereafter referred to as E‐P loci), whereas 513 SNPs in 222 genes evolved specifically in response to dietary adaptation. The former represent “high confidence” loci underlying the evolution of longevity and correlated life history traits, whereas the latter represent candidate loci underpinning developmental life history adaptation. These loci may be involved in the consistent differences in lifespan, adult size, and development time that were observed in response to postponed reproduction and adaptation to larval diet, respectively, independent of the second selective regime (May et al., 2019).

Importantly, even though cluster analyses indicated an interaction between the two regimes for the remaining 61% of differentiated SNPs, no significant divergence was observed among the late‐reproducing (P) populations that had evolved on different larval diets for any of the clusters. This indicates that the interaction between the regimes is driven by the early reproducing (E) populations, supporting the notion that the evolution of lifespan and life history in response to postponed reproduction is not heavily constrained by adaptation to diet during larval development. This independence of the selection regimes is also underscored by the clear, distinct grouping of the long‐lived P populations within a monophyletic cluster in terms of pairwise allele frequency differences of randomly drawn SNPs (Fig. 2).

Our findings are interesting in view of previous work showing that larval food conditions impact developmental time, adult body size, and fecundity but—in most cases—not lifespan (e.g., Zwaan et al. 1991; Tu and Tatar 2003; but see below). Similarly, selection experiments have not consistently found genetic correlations between development time, body size, and longevity, suggesting that development might not have a major effect upon longevity (e.g., Chippindale et al. 1994; Zwaan et al. 1995a,b). This is also consistent with an EE experiment showing that adaptation to chronic larval malnutrition affects development time, body size, and early fecundity, but not lifespan (Kolss et al. 2009). On the other hand, recent evidence shows that developmental—not only adult—DR has also the potential to extend lifespan, in part conditional upon adult diet conditions (e.g., May et al. 2015; Stefana et al. 2017).

### FLIES ADAPTED TO LOW DIET AND EARLY REPRODUCTION ARE LONG‐LIVED AND GENETICALLY DISTINCT

Given that developmental DR can extend lifespan, it is noteworthy that in our experiment adaptation to the L diet led to the evolution of extended lifespan in the E, but not in the P populations. In addition, the LE populations had the strongest decrease in development time on low diet, the lowest adult weight, and decreased early fecundity (May et al. 2019). These findings are mirrored by our genomic results: all LE populations clustered clearly together in terms of pairwise allele frequency differences, separated from all other populations (Fig. 2). Indeed, among the 61% of divergent SNPs (*n* = 1340) that showed evidence for an interaction in the cluster analyses, the vast majority (83%, 1112 SNPs in 241 genes; cluster 7) was due to divergence of the LE populations away from all the other populations (Fig. 4, Supporting Information Result S5, Table S1). Moreover, we observed a significant decrease in nucleotide diversity (*π*) and Tajima's *D* for two large regions on chromosome 2L and 3R, suggesting that the LE populations have experienced strong(er) sweeps (Supporting Information Result S6). The number of neutral loci that are genetically linked to loci under selection may be elevated in these large regions, potentially leading to an overestimation of the differentiation of the LE regime (also see Fig. 3 of Supporting Information Result S5 for the genomic location of LE loci). Nonetheless, these results show that the LE populations are distinct from all other EE populations and represent the principal cause of the interaction between the regimes; they also indicate that the LE regime has imposed stronger selection on life history adaptation than the other regimes.

As early egg production in *Drosophila* is determined by dietary resources acquired during development, while later fecundity is mainly affected by resources acquired during adulthood (Min et al. 2006; O'Brien et al. 2008), the nutritional conditions during the larval stage were likely to be more important for the E populations than for P populations. We conjecture that the combination of the “low” developmental diet and the E regime might have constrained flies to evolve reduced fecundity early in life, potentially with a longer lifespan as a pleiotropic side effect of reduced early‐life fecundity. The loci that differentiate the LE regime from the other populations (hereafter referred to as LE loci) are therefore of particular interest: they not only represent candidates for early life history adaptation, but also loci that may be responsible for developmental effects upon lifespan, or which might play a role in trade‐offs between lifespan and early fecundity. These LE loci are qualitatively clearly distinct from the longevity loci shaped by selection for postponed reproduction, with only 10 genes (3%) overlapping between the two sets of genes (Supporting Information Result S5).

Overall, our genomic analysis revealed both private responses and interactions between the selective regimes, which mirrors the phenotypic observations by May et al. (2019).

### EVOLVED CANDIDATE LOCI ARE QUALITATIVELY DIFFERENT FROM ‘CANONICAL’ LONGEVITY GENES

We next sought to examine the biological and molecular functions of the identified candidate loci, especially those implicated in the evolution of longevity. Molecular genetic analyses and mutant screens have successfully identified many genes involved in the regulation of lifespan, most famously in the IIS and TOR pathways that have evolutionarily conserved effects on longevity (e.g., Kenyon 2001; Partridge and Gems 2002; Tatar et al. 2003). Yet, whether loci in these “canonical” pathways harbor segregating alleles that contribute to the evolution of lifespan in natural populations is poorly understood (Flatt 2004; Flatt and Schmidt 2009; Remolina et al. 2012; Flatt and Partridge 2018). Key questions here are do evolved alleles map to previously identified major‐effect loci in these pathways? Or is the evolutionary basis of quantitative phenotypes such as lifespan and correlated life history traits highly context‐dependent and not “predictable” from knowledge of large‐effect mutants or transgenes?

To address these questions, we first used two complementary approaches to test for functional enrichment: (1) gene ontology (GO) and (2) gene set enrichment (GSEA) analyses. Method (1) identifies significant overlaps between user‐defined gene lists and gene ontology information from curated databases, using an arbitrary cut‐off, whereas method (2) essentially analyzes all the genes in the dataset without a cut‐off. Neither the GO analysis nor GSEA detected any significant enrichment of specific biological and molecular functions in our list of candidate targets of selection (Tables S2 and S3, tests were done on the complete set of candidate genes, as well as on subsets of “E‐P”, “diet”, “interaction”, and “LE” genes, and “LE” genes). This implies that the candidate loci underlying both dietary adaptation and the evolution of longevity are functionally diverse. Also, it might reflect the multivariate selection on different sets of correlated life history traits between the two regimes, including developmental time, adult size and female fecundity, which depend at least partly on distinct genetic pathways (Zandveld et al., 2017; May et al., 2019). Finally, hitchhiking loci that are genetically linked to causative sites may hamper the detection of functional enrichment, which may be a problem in particular for regions with low recombination rates. The address this latter issue, we also analyzed our set of candidates after removing non or low‐recombining regions of the genome (in which elevated levels of linked evolution may occur), but excluding these regions did not change our conclusions (see Supporting Information Result S7).

In terms of the genetic basis of aging, the fact that we failed to find an overlap with the GO term “determination of adult lifespan” suggests that our candidate set is not enriched for “canonical” longevity genes, which have been previously discovered using analyses of large‐effect mutants and transgenes. To independently verify this result, we compared our candidates to the comprehensive *GenAge* database (Tacutu et al. 2013), a list containing *Drosophila* genes with an experimentally confirmed role in aging, as determined by genetic manipulations (*n* = 188 genes, though this is likely an underestimation of the true number of longevity genes in *Drosophila*). The overlap between our complete list of candidate loci and those in the *GenAge* database was very small (*n* = 10 genes, *P* = 0.228, Bonferroni: 0.05/43 intersections tested in total = 0.0012). The overlap increased to 31 genes (*P* = 0.066) when we included orthologous longevity genes from other species (*n* = 579 genes), but also this was not significantly different from chance (Tables S4 and S5). The E‐P genes, which are the most promising longevity candidates, in fact have no overlap at all with the *Drosophila* aging genes from *GenAge* and only three genes (*P* = 0.905) when orthologous genes are included in the analyses. The overlap is bigger for the LE genes: five (*P* = 0.135) and 15 genes (*P* = 0.020), respectively, but also this overlap was not significant after correction for multiple testing.

Contrary to the expectation that standing genetic variation in the IIS/TOR pathways might make a major contribution to the evolution of longevity and correlated life history traits (*cf*. discussion in Remolina et al. 2012; Fabian et al. 2018; Flatt and Partridge 2018), we only found a few loci in this signaling network among our candidates. For instance, among the loci significantly differentiated between the E and P populations, we identified the TOR signaling gene *happyhour* (Bryk et al. 2010; Khan et al. 2012) and the gene *Psa*, known to interact with the major IIS/TOR core component *Akt1* (Vinayagam et al. 2016). Similarly, among the loci that differentiate the LE populations from the other EE populations we identified *Tomosyn*, a regulator of insulin secretion with a confirmed role in aging in *C. elegans* (Ch'ng et al. 2008), as well as *Pi3K21B* and *PKCδ*, which both play a role in signal transduction from the insulin receptor (Braiman et al. 2001; Teleman 2010). These loci may play an important role in the evolution of lifespan and life history in our EE populations, even though the IIS/TOR pathways as a whole were not quantitatively enriched in our dataset. The failure to find a clear overlap with well‐known longevity loci is also consistent with other recent “evolve and resequence” studies of *Drosophila* lifespan (Remolina et al. 2012; Carnes et al. 2015; Fabian et al. 2018). Similar to ours, these studies found only weak support for an involvement of loci in the IIS/TOR or other major longevity pathways in the evolution of longevity (Table S5).

This is an interesting observation given that functional variation in canonical “aging” genes in natural populations has been observed to contribute to life history adaptation along latitudinal clines, for example the *Drosophila* Insulin receptor (*InR*) (Paaby et al. 2010; Paaby et al. 2014), transcription factor *foxo* (Durmaz et al. 2019), and *methusaleh* (Paaby and Schmidt 2008). However, the selection pressures to which these alleles respond in clinal populations may be different from selection for postponed reproduction or adaptation to larval diet such as in our experiment. Also, it remains unknown whether the SNP variants in “aging” genes, which we have identified in our study, have functional effects on the phenotype.

A major conclusion emerging from these studies is, therefore, that genic targets of selection for longevity in evolving populations are qualitatively different from the loci identified in analyses of mutants or transgenes (also see Fabian et al. 2018; Flatt and Partridge 2018). This might not be surprising given that mutant screens are biased toward discovering large‐effect alleles (with likely deleterious fitness effects), whereas “E&R” studies are geared toward identifying segregating “minor effect” polymorphisms from natural populations. The fact that most longevity loci discovered in “E&R” studies are novel thus suggests that we might still be far away from reaching “saturation” in terms of understanding the complex genetic architecture of longevity (*cf*. Pollock and Larkin 2004 for a discussion of “saturation” in mutant screens). In addition, “E&R” studies provide an opportunity to capture the full genetic complexity of lifespan, including epistatic interactions and/or antagonistic pleiotropic effects, which may help us to better understand the genetic basis of variation in lifespan. This makes “E&R” studies a valuable method for identifying longevity and life history genes that is complementary to classical mutant screens.

### A CORE SET OF LONGEVITY CANDIDATES IS SHARED ACROSS INDEPENDENT EXPERIMENTS

Despite the fact that most longevity candidate loci in “E&R” studies are novel and do not overlap with previously identified “classical” longevity genes, it is interesting to ask how many of them might be found repeatedly across independent datasets. Given the highly polygenic nature of lifespan one might expect that most evolved longevity loci are population specific (i.e., private), at least at the level of individual SNPs. On the other hand, parallel and convergent evolution in independent populations might result in the repeated use of the same genes underlying a given trait (“gene reuse”; Conte et al. 2012).

**Figure 5:**
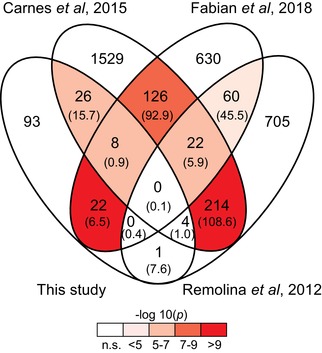
Significant overlap of our candidate longevity (E‐P) genes identified in our study with candidate loci from three other, independent longevity E&R studies (Remolina et al. 2012; Carnes et al. 2015; Fabian et al. 2018). Both the observed overlap and the expected overlap (in brackets) are shown. The overlap of our candidate loci with Carnes et al. (2015) and Fabian et al. (2018) was higher than expected by chance (*P* < 0.0014; Bonferroni correction: 0.05/43 intersections). Significant overlaps of the different intersections are indicated by shades of red.

To address this question, we compared our list of lifespan candidates (i.e., E‐P genes) to those from three previous, independent “E&R” experiments of *Drosophila* longevity that also applied selection on postponed reproduction. An extended lifespan evolved in all studies, in concert with correlated responses in fecundity, but the effects on development time and adult size differ among four independent studies (Remolina et al. 2012; Carnes et al. 2015; Fabian et al. 2018; see Supporting Information Methods for additional information on these E&R studies). Notably, at the candidate gene level (but not at the SNP level), we identified a significant overlap with the datasets of Carnes et al. (2015) (*P* = 3.5 × 10^−6^) and Fabian et al. (2018) (*P* = 2.6 × 10^−10^), but not with the dataset of Remolina et al. (2012) (*P* = 0.96). In total, 39.6% (*n* = 61) of our longevity candidates were also present in one or several of the other datasets (Fig. 5, Tables S4 and S6; the expected overlap being 32.2 genes, 20.9%).

Eight of our significant longevity (E‐P) loci showed a significant overlap with candidates identified by both Carnes et al. (2015) and Fabian et al. (2018). Six of them are involved in neuronal development or function (*Ace*, *Ptp10D*, *nmo*, *Pura*, *CG32373*, and *spg*), which is interesting considering the role of neuronal processes and neuroendocrine signaling in the regulation of lifespan (Tatar 2004; Alcedo et al. 2013). For example, the acetylcholinesterase gene *Ace* is known to affect lifespan in *C. elegans* (Xue et al. 2007), whereas the protein‐tyrosine phosphatase receptor gene *Ptp10D* has been previously identified as a candidate locus in another EE study of *Drosophila* longevity (Michalak et al. 2017).

Among the remaining overlapping candidates, which overlap with only one of the other E&R datasets, we identified several genes (e.g., *Doa*, *Beadex*, and *cappuccino*) that play a role in gonad development or reproduction (Quinlan 2013; Zhao et al. 2013; Kairamkonda and Nongthomba 2014)—they might thus represent loci with pleiotropic effects on fecundity and lifespan, which co‐evolved consistently in all four studies.

Interestingly, although we failed to find an overlap between our E‐P longevity candidate genes and those of Remolina et al. (2012), their candidate loci showed a significant overlap with our LE genes (*n* = 34, *P* = 2.9 × 10^−6^; Table S6). This finding may reflect differences in experimental design among the four studies. For instance, in contrast to May et al. (2019), which applied an egg laying window of 2‐4 hours, Remolina et al. (2012) used a procedure for setting up subsequent generations that allowed females to lay eggs over a period of seven days before collecting offspring at days 10–11. This procedure applied by Remolina et al. (2012) might have selected for shorter development time, in particular in the younger offspring, which is also the most strongly diverged phenotype in the LE populations. These results perhaps indicate that the evolution of lifespan in the Remolina et al. populations is not linked to postponed reproduction, but instead reflects how adaptation of the developmental phase is integrated in the adult life history, similar to the LE populations.

Overall, our results agree well with a similar recent overlap analysis by Fabian et al. (2018) showing that several longevity candidate loci are likely subject to parallel evolution and gene reuse across independent experiments. At the same time, our factorial design of two selective regimes made it possible to distinguish qualitatively different sets among these overlapping loci as shown above (*cf*. Zandveld et al., 2017).

### CONCLUSIONS

Whether and how developmental evolution facilitates or constrains the evolution of adult life history and aging remains poorly understood. To address this fundamental problem, we analyzed the genomes of *Drosophila* populations subject to both selection for larval dietary adaptation and longevity. Our main findings are that (1) the genetic evolution of longer adult life in response to postponed reproduction is largely independent of adaptation to nutritional conditions during development; (2) adaptation to a relatively poor larval diet in conjunction with selection for reproductive performance early in life also causes the evolution of longevity, yet the underlying loci differ from those involved in longevity selection via postponed reproduction; (3) genic targets of selection for longevity in evolving populations are qualitatively different from variants identified in mutant screens or analyses of transgenes; and (4) an appreciable proportion of evolved longevity loci overlap among independent populations, perhaps suggesting that there exist “preferred” genic targets of selection for lifespan and correlated traits. “E&R” studies may thus offer a powerful method for discovering new polymorphic loci that are involved in the evolution of longevity and life history, which might not be discoverable in classical mutant screens. Moreover, applying two or more selective regimes may help disentangle if genomic differentiation is related to lifespan or other life history phenotypes that evolve in concert.

Associate Editor: Z. Gompert

## Supporting information


Supplementary methods (pdf): Description of DNA extraction and all genome analyses.

Supplementary Results S1‐S8 (pdf):
**Supplementary Result S1**: Average coverage
**Supplementary Result S2**: Statistical model comparison
**Supplementary Result S3**: *P‐*value distribution of GLM
**Supplementary Result S4**: Allele frequency differentiation
**Supplementary Result S5**: Cluster analysis
**Supplementary Result S6**: π and Tajima's *D*
**Supplementary Result S7**: Low‐recombining regions
**Supplementary Result S8**: Cosmopolitan inversions

Supplementary Tables S1‐S7 (xls):
**Supplementary Table S1**: Significant SNPs as determined by GLM analysis
**Supplementary Table S2**: GO analysis
**Supplementary Table S3**: GeneSet analysis
**Supplementary Table S4**: Overview of candidate genes
**Supplementary Table S5**: Overlap with the *GenAge* database
**Supplementary Table S6**: Overlap among longevity E&R studies
**Supplementary Table S7**: SNP features
