# Supplementary material for: Distinct genomic signals of lifespan and life history evolution in response to postponed reproduction and larval diet in Drosophila
Source: Evol Lett. 2019 Oct 2;3(6):598–609. doi: 10.1002/evl3.143 (PMC6906992; doi:10.1002/evl3.143)
Supplement: Supplementary file 1 — Supplementary methods (pdf): Description of DNA extraction and all genome analyses. [file EVL3-3-598-s001.pdf]

Supplementary Methods

This Supplementary Methods file provides information on the DNA extraction protocol and on the analysis of the genome data. This includes information on genome assembly, SNP calling and annotation, the estimation of population genetic parameters π and Tajima's D, the analysis of four different statistical models for assessing SNP differentiation, the quantification of SNP differentiation using GLM and FDR calculation, the clustering analysis of random and significantly differentiated SNPs, functional enrichment analyses using Gowinda and GeneSet analyses, and analyses of the overlap of our candidate genes with the GenAge database and three other "longevity" E&R studies.

Pooled DNA extraction and sequencing

The pools of 250 female flies were frozen in liquid nitrogen, homogenized using a mortar and pestle, and then incubated in lysis buffer (100 mM Tris-Cl, 100mM EDTA, 1% SDS, 1 mg/ml Proteinase K) at 56°C for 30 min and 70°C for 30 min. After lysis, the samples were treated with RNAse A (3 mg per 250 μl aliquot) at 37°C for 30 min. Then 39 μl 8M potassium acetate solution was added and the samples were incubated on ice for 30 min to precipitate protein. The samples were centrifuged at 14.000 rpm for 15 minutes and the supernatant was transferred to a new tube. One volume of phenol-chloroform-isoamylalkohol (25:24:1) was added, the sample was thoroughly shaken en then centrifuged 5 min at 14.000 rpm. The upper aqueous phase was transferred to a new tube and washed with 0.75 volume of chloroform, by repeating the steps above. The DNA was then precipitated by adding three volumes of ice cold 100% ethanol and centrifuging for 15 min at 14.000 rpm at 4°C. After washing the pellet with 70% ethanol and allowing it to dry at room temperature, it was resuspended in 50 μl TE buffer. DNA was quantified on a Qubit 2.0 spectrophotometer (Life Technologies) and the quality was assessed with a Fragment Analyzer (Advanced Analytical). DNA libraries were prepared from 300 ng gDNA using the Illumina TruSeq Nano Library preparation kit; following the manufacturer’s instructions for 350 bp insert sizes. The 24 samples were sequenced on eight lanes (three multiplexed samples per lane) of a HiSeq 2500 sequencer.

Genome Assembly

The raw 100-bp paired-end reads were filtered for a minimum base quality of 20 and a minimum read length of 70 using *PoPoolation* v.1.2.2 (Kofler *et al*. 2011). Reads were then aligned to a “hologenome” reference, which consists of the

genomes of *D. melanogaster* (v.6.04) and microbial symbionts (hologenome adapted from http://bergmanlab.genetics.uga.edu/?p=2033) using the *mem* algorithm of *BWA* v.0.7.10 with default settings (Li 2013). Mapped reads were filtered for a minimum mapping quality of 30 using *samtools* v.1.1 (Li and Durbin 2009); duplicate reads were removed using *picard tools* v.1.130 (http://broadinstitute.github.io/picard); and the alignment around indels was optimized using *GATK* v.3.3.0 (McKenna *et al*. 2010). Insertion-deletions (indels) and their five flanking nucleotides (on either side) were masked if the indel was supported by at least 48 reads in total across all populations (Kapun *et al*. unpublished data). In addition, using *RepeatMasker* v.4.0.5 (http://www.repeatmasker.org) with default parameters, we generated a Genome Transfer Format (GTF) file to mask simple sequence repeats and transposable elements using the *D. melanogaster* genome v.6.04.

SNP Calling

Polymorphic sites were identified using conservative criteria (Fabian *et al*. 2012). We first excluded all sites with a coverage below 30 as well as sites that fell within the top 2% of the maximum coverage distribution of each sample since these may mostly represent copy number variations or assembly errors. We identified SNPs by pooling allele counts across all populations and considered only sites with a minor allele count of at least 48 as being polymorphic. This threshold minimizes the impact of sequencing errors; it assumes that alleles with a high allele count in one or few populations, or alleles that have a low number in multiple populations, are correctly called SNPs. In total, we identified 1'238'970 SNPs. Finally, SNPs were screened for markers that indicate the presence of inversion polymorphisms (Kapun *et al*. 2014). The average allele frequency of these inversion-specific marker SNPs was used to estimate inversion frequencies in the EE populations. This analysis indicated that the common cosmopolitan inversions *In(2L)t*, *In(3R)C* and *In(3R)Mo* occurred at very low frequencies (2-6% of the reads on average) in several populations. We did not find consistent frequency differences among regimes, which suggests that inversions do not play a role in our EE populations (Supplementary Result S8).

Estimation of Population Genetic Parameters

Two standard population genetic parameters, π and Tajima's *D* (Charlesworth and Charlesworth 2010), were calculated to characterize genome-wide patterns of genetic variation and differentiation. We used custom scripts to estimate π (Kapun *et al*. unpublished data) and *PoPoolation* v.1.2.2 (Kofler *et al*. 2011) to estimate Tajima's *D* for each polymorphic site. Reads were subsampled to a

coverage of 50x before calculating Tajima's *D*. We calculated average values of π and *D* for non-overlapping windows of 200 kB across the different chromosomal arms. ANOVA was used to analyze genome-wide patterns of π and Tajima's *D* in 200 kb windows, followed by Tukey's HSD post-hoc tests in *R*. Bonferroni correction was applied to account for multiple testing (see Supplementary Result S4 for more information).

Quantifying SNP Differentiation

Our EE design combines two selection regimes; we thus tested for 'main effects' of both regimes on SNP differentiation as well as their interaction. We assessed the performance of four different types of models on simulated datasets, created using different assumptions regarding selection intensity, population size, and initial allele frequencies: (1) ANOVA on arcsine square root transformed allele frequencies, (2) GLM with binomial error structure on the read counts, (3) GLM with a quasibinomial error structure, and (4) generalized linear mixed models (GLMM) with binomial error structure and using replicate population as a random effect. Details of these analyses, which demonstrated that the binomial GLM had the highest true discovery rate, are given in Supplementary Result S2.

We used the binomial GLM approach to analyze the read counts of all bi-allelic SNPs in our dataset, i.e. each read either represents the major allele ('1', defined as the allele that occurs most frequently among all 24 EE populations) or the minor allele ('0'). The model, implemented in *R* (v.3.2.2), included the two selection regimes ('developmental diet' and 'age at reproduction') and their interaction as fixed factors. *P*-values for the two main factors and the interaction term were obtained through analysis of deviance based on χ^2 tests using the *anova* function.

To correct for multiple testing, and to determine a cut-off for significantly differentiated SNPs, we calculated false discovery rates (FDR) following the procedure of Jha *et al*. (2015). To do so, the labels of the SNP dataset were permuted in a pseudo-randomized fashion: i.e., the labels (the identity) of the two selection regimes were randomly reassigned to the complete SNP dataset, with the criterion that randomization had to produce a maximal mismatch with the actually observed data. We applied two randomization criteria to do so: (1) A sample should never get the same treatment label (e.g. an sample 'LE', should never be 'LE' again, but could be for example HP, CE, LP, et cetera); and (2) replicates of a given regime combination can never be replicates of a newly assigned regime combination (e.g., the samples LE-1, LE-2 and LE-3 can never become HP-1, HP-2 and HP-3, but, for example, HP-1, LP-2 and CP-3). Apart from these criteria, samples were completely randomly drawn and assigned new

labels. We generated ten permuted datasets using this procedure (see Table 1 for identity of the samples in the ten permuted datasets used).

Table 1. Identity of the labels for the ten permuted datasets (P1-10). The labels indicate EE regime combinations: larval diet ("L", "C" or "H") and age-at-reproduction ("E" and "P").

True label	P1	P2	P3	P4	P5	P6	P7	P8	P9	P10
LE-1	LP	HE	HP	LP	HP	CE	CE	HE	CP	HP
LE-2	CP	CE	LP	HE	CE	CP	LP	LP	HP	CP
LE-3	CE	LP	CP	HP	LP	HP	CP	HP	HE	HE
LE-4	HE	CP	CE	CP	HE	LP	HE	CP	LP	CE
CE-1	LE	CP	HE	CP	CP	LE	HE	LP	HE	CP
CE-2	HP	HP	LP	LP	HE	LP	LE	HP	HP	LP
CE-3	LP	LP	LE	HP	HP	CP	HP	HE	LE	LE
CE-4	CP	LE	HP	HE	LP	HE	CP	LE	CP	HP
HE-1	HP	CE	CP	LP	CE	CP	CE	CE	HP	LE
HE-2	LE	LP	CE	HP	CP	CE	LP	LE	CE	CE
HE-3	LP	HP	LP	CE	LP	HP	CP	LP	CP	CP
HE-4	CE	LE	HP	LE	LE	LP	HP	CP	LP	LP
LP-1	CP	CP	CE	LE	CP	LE	CE	CP	CP	HP
LP-2	LE	HE	CP	HE	HP	HE	HP	HP	LE	CP
LP-3	HE	HP	LE	CE	HE	HP	HE	HE	CE	CE
LP-4	HP	CE	HE	CP	LE	CP	LE	CE	HP	HE
CP-1	HP	LE	HP	HE	LE	HP	LE	CE	LE	HP
CP-2	HE	CE	LP	LE	HP	HE	HP	HP	CE	LE
CP-3	CE	HP	HE	CE	CE	LE	LP	LE	LP	LP
CP-4	LE	HE	LE	HP	HE	CE	HE	HE	HE	HE
HP-1	CP	HE	CE	LE	LE	LP	LE	CP	LP	LP
HP-2	CE	LP	LE	CE	CP	CE	CP	LP	HE	LE
HP-3	LP	LE	CP	LP	LP	LE	LP	CE	LE	HE
HP-4	HE	CP	HE	CP	CE	HE	CE	LE	CE	CE

Using GLM with the original sample labels as factors, we calculated P-values for every SNP to obtain an "observed" distribution of *P*-values with an unknown proportion of false positives. In addition, we also calculated *P*-values for the complete dataset ten times using each of the ten sets of pseudo-randomized labels to obtain an "expected" *P*-value distribution (i.e. empirical null distribution), which is determined by factors that are not directly related to the effects of the experimental design. *P*-value distribution plots revealed that all randomized datasets had very similar distributions (see Supplementary Result S3 for the

averaged *P*-values distribution plot of empirical and randomized datasets and the lowest *P*-values for each set). We then ranked the genome-wide *P*-values of the true labels and each of the *P*-value datasets of the ten permutations. For every "true" *P*-value we calculated the proportion of permuted *P*-values with a lower value than a given *P*-value. This value is equivalent to an FDR value as defined by Storey and Tibshirani (2003). Since we generated ten permuted datasets, we calculated the FDR for the observed dataset with each of the ten permuted datasets individually (i.e., for each SNP we calculated the FDR ten times). We then averaged these ten FDRs by calculating one mean FDR per SNP, to level out variation among the ten permuted datasets. The mean FDR for each SNP value was used for further analyses.

We used a conservative cut-off of FDR=0 to identify significantly diverged SNPs, i.e. we only considered SNPs with a lower *P*-value than any of the *P*-values observed in the permuted datasets to be significantly diverged. These SNPs are the most extremely differentiated of the dataset and are therefore most likely to include targets of selection."

Clustering Analysis of Random and Significantly Differentiated SNPs

To visualize the overall genetic diversity among the 24 EE populations, we constructed a clustering tree by analyzing pairwise differences among populations based on 6500 randomly drawn SNPs (1000x bootstrapped) using the package *PHYTOOLS* in *R*. To quantify the number of distinct patterns in allele frequencies among significantly diverged loci, all significant SNPs identified by GLM were clustered using hierarchical clustering (“average” method, *hclust* package in *R*). Details of this analysis and results are given in Supplementary Result S5.

Annotation

SNPs were annotated with *snpEff* v.4.1e using the annotations for the *D. melanogaster* genome v.6.04 from FlyBase. SNPs that occurred within 200 bp of a gene were considered to represent upstream or downstream variants that might play a role in the transcriptional regulation of the corresponding gene. SNPs were classified into the following categories: ‘non-synonymous’, ‘synonymous’, ‘UTR’, ‘intronic’, ‘up- or downstream’, and ‘intergenic’. SNPs may have multiple annotations if they fall within overlapping genes or if they can be classified into multiple categories. In total, we identified SNPs located in 16'777 genes (see Supplementary Table S7 for features of annotated SNPs).

Enrichment and Functional Analyses

We performed two complementary functional analyses to examine genetic pathways and functional mechanisms that may have been shaped by selection in our EE populations. First, we investigated enrichment of gene ontology (GO) categories among sets of significant SNPs using *Gowinda* (v.1.12) (Kofler and Schlotterer 2012). We used annotations from the *D. melanogaster* genome v.6.13 and a GO association file from FuncAssociate3.0 (Berriz *et al*. 2003; http://llama.mshri.on.ca/funcassociate) with the following settings: '--mode gene', '--gene-definition updownstream200', '--simulations 100000'. Second, we used a gene set analysis following the procedure described by (Daub *et al*. 2013; https://github.com/CMPG/polysel). In contrast to the first method, this approach essentially analyzes all the genes in the dataset without a cut-off for the test statistic used. We used the *P*-values of the GLM analysis for this purpose. Gene sets and pathways that are evolutionary conserved or specific to *D. melanogaster* were downloaded from the KEGG (386), BIOCYC (250) and REACTOME (1225) databases through the NCBI Biosystems database. After removing gene sets with fewer than 10 genes and merging sets with high similarity, 16507 genes in 635 gene sets were included in this analysis.

Overlap among Candidate Gene Lists

To identify 'canonical' longevity genes among our candidate genes, we calculated the overlap of our dataset with genes from the *GenAge* database, a dataset that enlists genes that have experimental support for being involved in the regulation of lifespan (Tacutu *et al*. 2013). This database contains 188 *Drosophila* genes with a known role in aging as well as 391 'aging' genes in other model species that have orthologs in *D. melanogaster* (http://genomics.senescence.info/genes/models.html). The Entrez IDs of these genes were converted to FlyBase IDs using *DAVID* v.6.8 (Huang *et al*. 2009). The package "*SuperExactTest*" (v0.99.4) (Wang *et al*. 2015) in *R* was used to test for a significantly higher overlap than expected by chance among our dataset and the *GenAge* database. We used the total number of SNPs (1'238'970) and genes with SNPs (16'777) observed in our study as parameters in the analysis. Bonferroni correction (α = 0.05/43 intersections analysed with the *SuperExactTest* in total = 0.0012) was applied to account for multiple testing. We also determined whether there are overlaps between the 'aging' genes from *GenAge* and three published longevity E&R datasets (Remolina *et al*. 2012; Carnes *et al*. 2015; Fabian *et al*. 2018). All three studies used selection on postponed reproduction to evolve a longer lifespan, although details of the selection procedures vary. The three studies all used outbred laboratory populations originating from the USA, but the number of generations these

populations had been reared in the lab before onset of EE is variable (<0.5 year (Fabian *et al*. 2018) *versus* 5-7 years (Remolina *et al*. 2012; Carnes *et al*. 2015). Also the number of generations of EE differs, ranging from 80/50 (E/P regime, respectively) (Remolina *et al*. 2012), 293/144 (Fabian *et al*. 2018), to 850/170 (Carnes *et al*. 2015) generations. Correlated responses in other life history traits, such as fecundity (observed among all populations), development time (observed in the populations studied by Carnes *et al*. 2015), or size (no correlated response observed in the populations studied by Carnes *et al*. 2015 and Fabian *et al*. 2018) among others, were also observed for these EE populations, although the correlated responses differ per study. Next, we investigated the overlap of our dataset with these three longevity E&R studies. To do so, we converted the coordinates from the three published E&R studies to v.6 using the online conversion tool available at FlyBase (http://flybase.org/static_pages/downloads/COORD.html). To compare genes, the published FlyBase IDs of all studies were updated to the most recent IDs using the online tool at FlyBase (http://flybase.org/static_pages/downloads/IDConv.html). The *SuperExactTest* package was used to test for enriched overlaps as described above.

References

Berriz, G.F., King, O.D., Bryant, B., Sander, C., Roth, FP. (2003). Characterizing gene sets with FuncAssociate. *Bioinformatics* 19:2502-2504.

Carnes, M.U., Campbell, T., Huang, W., Butler, D.G., Carbone, M.A., Duncan, L.H., et al. (2015). The Genomic Basis of Postponed Senescence in *Drosophila melanogaster*. *PLoS ONE* 10:e0138569.

Charlesworth, B., Charlesworth, D. (2010). *Elements of Evolutionary Genetics*. Roberts and Company, Greenwood Village, Colorado.

Daub, J.T., Hofer, T., Cutivet, E., Dupanloup, I., Quintana-Murci, L., Robinson-Rechavi, M., *et al*. (2013). Evidence for Polygenic Adaptation to Pathogens in the Human Genome. *Mol Biol Evol*. 30:1544-1558.

Fabian, D.K., Kapun, M., Nolte, V., Kofler, R., Schmidt, P.S., Schlotterer, C., *et al*. (2012). Genome-wide patterns of latitudinal differentiation among populations of *Drosophila melanogaster* from North America. *Mol Ecol*. 21:4748-4769.

Fabian, D.K., Garschall, K., Klepsatel, P., Santos-Matos, G., Sucena, E., Kapun, M., *et al*. (2018). Evolution of longevity improves immunity in *Drosophila*. *Evol Lett*. DOI: 10.1002/evl3.89.

Huang, D.W., Sherman, B.T., Lempicki, R.A. (2009). Systematic and integrative analysis of large gene lists using DAVID bioinformatics resources. *Nat Protoc*. 4:44-57.

Jha, A.R., Zhou, D., Brown, C.D., Kreitman, M., Haddad, G.G., White, K.P. (2016). Shared Genetic Signals of Hypoxia Adaptation in *Drosophila* and in High-Altitude Human Populations. *Mol Biol Evol*. 33:501-517.

Kapun, M., Barron Aduriz, M.G., Staubach, F., Vieira, J., Obbard, D., Goubert, C., *et al*. (2018). Genomic analysis of European *Drosophila populations* reveals longitudinal structure and continent-wide selection. *bioRxiv*: https://doi.org/10.1101/313759.

Kapun, M., Van Schalkwyk, H., McAllister, B., Flatt, T., Schlötterer, C. (2014). Inference of chromosomal inversion dynamics from Pool-Seq data in natural and laboratory populations of *Drosophila melanogaster*. *Mol Ecol*. 23:1813-1827.

Kofler, R., Orozco-terWengel, P., De Maio, N., Pandey, R.V., Nolte, V., Futschik, A., *et al*. (2011). PoPoolation: a toolbox for population genetic analysis of next generation sequencing data from pooled individuals. *PLoS ONE* 6:e15925.

Kofler, R., Schlötterer, C. (2012). Gowinda: unbiased analysis of gene set enrichment for genome-wide association studies. *Bioinformatics* 28:2084-2085.

Li, H. (2013). Aligning sequence reads, clone sequences and assembly contigs with BWA-MEM. *arXiv*:1303.3997.

Li, H., Durbin, R. (2009). Fast and accurate short read alignment with Burrows-Wheeler transform. *Bioinformatics* 25:1754-1760.

McKenna, A., Hanna, M., Banks, E., Sivachenko, A., Cibulskis, K., Kernytsky, A., *et al*. (2010). The Genome Analysis Toolkit: A MapReduce framework for analyzing next-generation DNA sequencing data. *Genome Res*. 20:1297-1303.

Tacutu, R., Craig, T., Budovsky, A., Wuttke, D., Lehmann, G., Taranukha, D., *et al*. (2013). Human Ageing Genomic Resources: Integrated databases and tools for the biology and genetics of ageing. *Nucleic Acids Res*. 41:D1027-D1033.

Remolina, S.C., Chang, P.L., Leips, J., Nuzhdin, S.V., Hughes, K.A. (2012). Genomic basis of aging and life-history evolution in *Drosophila melanogaster*. *Evolution* 66:3390-3403.

Storey, J.D., Tibshirani, R. (2003) Statistical significance for genomewide studies. *Proc Natl Acad Sci U S A.* 100:9440-9445.

Wang, M.H., Zhao, Y.Z., Zhang, B. (2015). Efficient Test and Visualization of Multi-Set Intersections. *Sci Rep*. 5:16923.
