# Supplementary material for: Distinct genomic signals of lifespan and life history evolution in response to postponed reproduction and larval diet in Drosophila
Source: Evol Lett. 2019 Oct 2;3(6):598–609. doi: 10.1002/evl3.143 (PMC6906992; doi:10.1002/evl3.143)
Supplement: Supplementary file 2 — Supplementary Results S1‐S8 (pdf): Supplementary Result S1: Average coverage Supplementary Result S2: Statistical model comparison Supplementary Result S3: P‐value distribution of GLM Supplementary Result S4: Allele frequency differentiation Supplementary Result S5: Cluster analysis Supplementary Result S6: π and Tajima's D Supplementary Result S7: Low‐recombining regions Supplementary Result S8: Cosmopolitan inversions [file EVL3-3-598-s002.pdf]

Supplementary Result S1: Average coverage

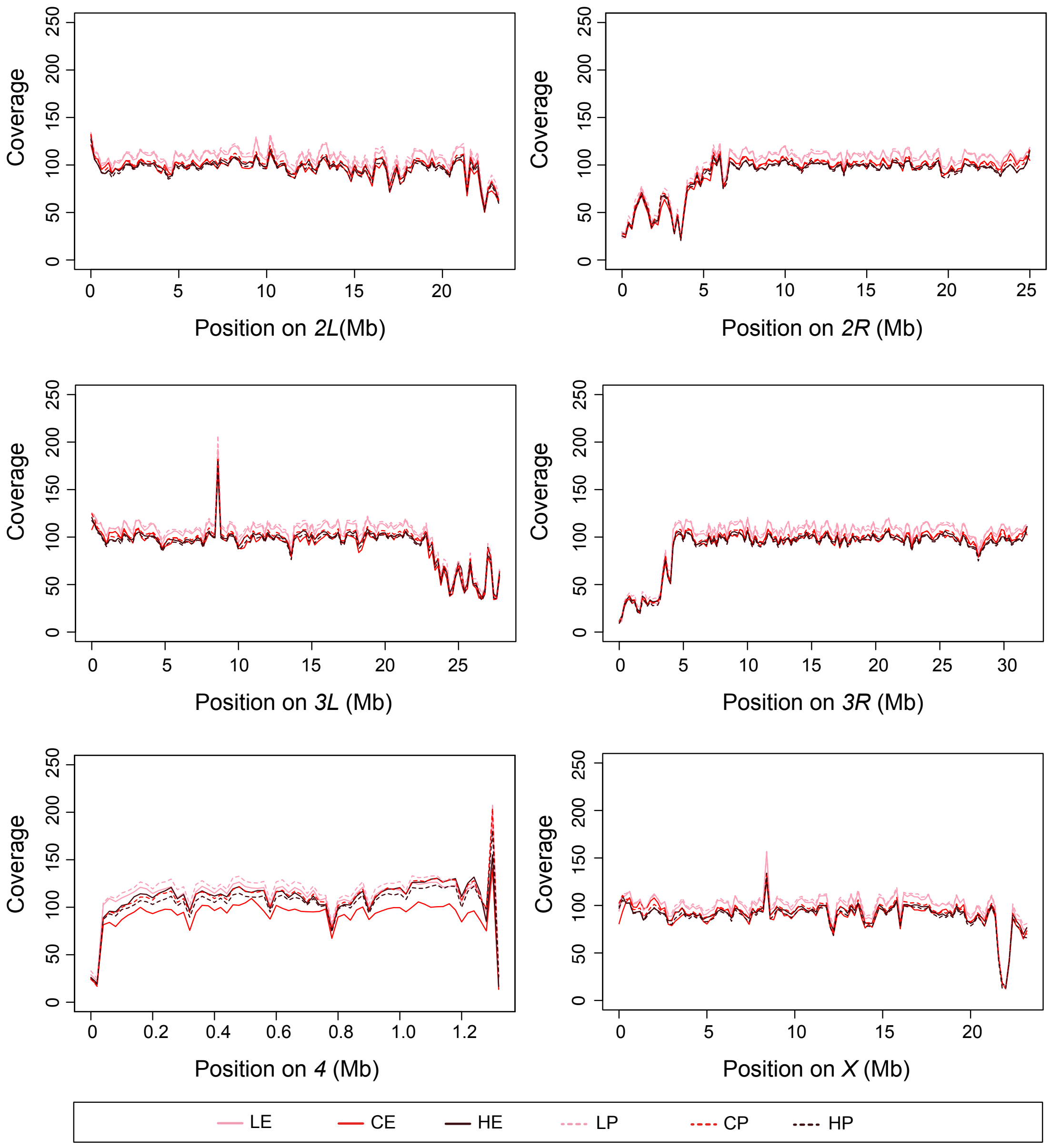

Figure 1: The average coverage per experimental evolution (EE) regime along each chromosome as calculated per 200 kb window (20 kb for the 4th chromosome) is shown. EE regimes (Developmental diet x Age-at-reproduction): low (L), control (C) and high (H) diet, and early (E) versus postponed (P) reproduction.

Supplementary Result S2: Statistical model comparison

Summary and conclusion

<u>**Aim and approach:**</u>

We used simulated datasets to test which statistical method (an ANOVA of arcsine square root transformed allele frequencies, GLM with binomial error distribution, GLMM with allele counts nested in line, or GLM with quasi-binomial error distribution ("QGLM")) is most appropriate to analyze the effects of our two selective regimes (i.e. main effects) and the interaction of the two in a single model. We tested the effects of starting allele frequency, population size and selection intensity on the performance of the different models.

<u>**Model performance**</u>

Overall, the true discovery rate (TDR) was highest for higher starting allele frequencies, higher selection intensities, and a larger population size for all models. Furthermore, TDR was highest for "Age-at-reproduction", intermediate for "Development diet" and lowest for "Interaction", indicating that SNPs detected as significant for "Age-at-reproduction" are more likely to be true candidates. The power to detect the interaction of the two regimes is relatively low for all models tested. For example, for "Age-at-reproduction" TDRs of up to 0.9 are observed, depending on the parameters used, whereas the TDRs for the interaction are ~0.5 maximum. Of the four different models, the highest TDRs were in general observed for the GLM with binomial error structure (except for a few parameter combinations) for different values of initial allele frequencies, selection intensities and for the three contrasts.

Next, we examined the correlation of allele frequency differentiation (dAF) with *P*-value for the different models. Under the assumption that higher selection intensities should lead to larger allele frequency differences, dAF should be a good predictor for selection. We observed that the correlation between dAF and *P*-value is highest for GLM, for both main factors and the interaction contrast.

Finally, we assessed the pairwise correlation in *P*-values between the four models. While generally speaking the shapes of the correlation plots are positive, these plots indicate that the most significant loci for a test would be unlikely to be the most significant in another test. This emphasizes the necessity to test model performance.

<u>**Conclusion**</u>

The GLM with binomial error structure was deemed most suitable for the detection of allelic differentiation associated with our selection regimes using our simulated datasets, and was therefore chosen for further analyses of the

EE genome dataset. This model does not account for overdispersion associated with E&R PoolSeq data, resulting in unrealistically low *P*-values, which need to be corrected (see main text, Supplementary Methods and Supplementary Result S3 for our approach and additional information). Full results from all four models on our real dataset are available upon request from the authors.

Simulation approach

Our simulations are based on population genetic assumptions to model evolving populations that are subjected to both drift and selection. We neglect effects of linkage disequilibrium, although, at least to some extend, these are likely to exist. We model selection from a starting allele frequency (q_0) by using a selection intensity of *s* and for a generation time of *t* generations. We assumed that we can use the formula B3.1.5 from Charlesworth and Charlesworth (2010) with the adaptation for diploids for allelic changes, such that:

$$q_t = \frac{1}{1+\frac{p_0}{q_0}\exp\left(-\frac{t}{2}s\right)} \qquad (1)$$

This assumption is valid when the effective population size is large enough, approximately >200 individuals (depending on *s*). The 24 EE populations were kept at a population size of ~2000-4000 individuals throughout the EE. Although the effective population size is unknown and dependent on the selection pressure, we expect that it is large enough to fulfill the assumptions for equation (1). To be able to model the different selection regimes for "Developmental diet", "Age-at-reproduction" and "Interaction", we considered six different selection intensities, representing the six different treatment levels, i.e., LE, CE, HE, LP, CP and HP. Out of every 1000 simulated SNPs, we considered 925 SNPs evolving under drift and 75 potentially under selection. Out of the 75 under selection, 25 were randomly assigned a "Developmental diet" effect, 25 an "Age-at-reproduction" effect and 25 an "Interaction" effect. As a result, a maximum of 75 SNPs were under selection, however, if some SNPs were under selection from both selection regimes, the total number of SNPs under selection would be lower than 75.

If a SNP was assigned an "Age-at-reproduction" effect, E populations were randomly assigned a value of s_r or -s_r while P populations were then assigned selection intensities of -s_r or s_r, respectively. If a SNP was assigned a "Developmental diet" effect, one of the levels was randomly assigned an effect of s_d, another -s_d and a third 0. Lastly, to model an "Interaction" effect we randomly chose two of the six treatment levels and assigned again one with s_i and one with -s_i as selection intensity. For every SNP we, therefore, summed up the values for selection intensities (i.e., s_r, s_d *and* s_i) that were used to generate the average allele frequency given the initial allele

frequency, generation time and selection intensity. Similar to the EE experiment, every treatment level was replicated four times. To simulate variation between replicate lines due to drift we used a Wright-Fischer model where variance is:

$$V(q_t) = q_t(1 - q_t)[1 - (1 - \frac{1}{2N})^t] \qquad (2)$$

While this is a model for drift, for small selection intensities the variance between replicate populations very much resembles variances under drift alone. Using 24 values of s, 115 and 58 generations for the Early and Postponed reproduction populations (i.e. *t*), respectively, and three different populations sizes N, we sampled 24 allele frequencies (q_s) using the combination of equations (1) and (2). Then we performed two types of further sampling. First we sampled for each population 500 alleles from a binomial distribution with the mean allele frequencies q_s as a mean and then calculated the allele frequency q_g. This resembles the sampling of 250 diploid females in the gDNA pool. Lastly, lines were given an average sequencing depth (S_l), randomly chosen from a Poisson distribution with mean of 100. Then for each SNP again we sampled a coverage from a Poisson distribution with mean S_l and used this number as coverage to sample the allele frequency q_g, which results in a measured sequencing depth S_m and measured allele frequency q_m for every population. This results in a simulated dataset that mimics the application of PoolSeq on evolving populations. Although the allele frequencies at generation 1 follow a more or less binomial distribution, the variance between replicate populations becomes progressively larger than expected from sequencing variation only (i.e. overdispersion), similar to "real" E&R datasets (see Figure 1) (Jonas *et al*. 2017).

Once these measured allele frequencies and coverages were determined, these values were saved for the 24 populations and further statistics were performed. Each simulated SNP was analyzed using four statistical models (ANOVA with arcsine square root transformed allele frequencies (Kelly *et al*. 2013), GLM with binomial error distribution (Martins *et al*. 2014), GLMM with allele counts nested in line (Jha *et al*. 2015; Jha *et al*. 2016), and a GLM with quasi-binomial error distribution ("QGLM") (Wiberg *et al*. 2017) . The latter two models (GLMM and QGLM) account for overdispersion that is commonly observed in population genomic PoolSeq data (Lynch *et al*. 2014; Wiberg *et al*. 2017; Kelly and Hughes 2019). All analyses were performed in *R* (v.3.3.1). ANOVA was performed using the function *aov*, the binomial and quasibinomial GLM were performed using the function *glm* and *P*-values were obtained through a chi-squared test using the *anova* function, GLMM was performed using the package *lme4* (v.1.1-13) and *P*-values were obtained through a likelihood ratio test performed using the *anova* function.

The resulting *P*-values for all the SNPs simulated to be under selection were then compared to SNPs in which all values for *s* were zero, i.e., SNPs segregating under drift alone. We separated the analyses for "Developmental diet", "Age-at-reproduction" and "Interaction". For each of these terms we sorted all SNPs under drift and selection and found the *j*th *P*-value. We calculated True Discovery Rate (TDR) as the number of SNPs with a lower *P*-value than the *j*th *P*-value divided by the total number of SNPs that were called significant. We present TDR as it indicates how well the "true" selection alleles are represented among a predefined quantity of the most significant alleles; it is the inverse of false discovery rate for a set cut-off in our calculations.

Effects of starting allele frequency and selection intensity, contrast and model

We started with simulating selection for fixed starting allele frequencies (0.01, 0.02, 0.05, 0.1, 0.2, 0.3, 0.4, 0.5), three effective population sizes (250, 500, 750) and for five selection intensities (0.01 – 0.05 in steps of 0.01). In total 24,000 SNPs were simulated. Furthermore, we set an artificial threshold for "significant divergence" at either 100, 250 or 500 most significant SNPs. All factors were analyzed in pairwise plots, combining two of the factors to uncover potential interactions in TDR. Averaged over the four models, a higher starting allele frequency and higher selection intensity increased the true discovery rate (see Figure 2A). Furthermore, TDR was highest for "Age-at-reproduction", intermediate for "Development diet" and lowest for "Interaction" (see Figures 2B and 2C) indicating it is more likely that SNPs detected, as "Age-at-reproduction" significant SNPs are true candidates. Analyses using GLM resulted in the highest TDR for different values of initial allele frequencies (Figure 2D), selection intensities (Figure 2E) and for the three contrasts (Figure 2F). An exception is that an initial allele frequency of 0.5, for which GLM had equal TDR as the GLMM.

Effective population size and cut-off stringency

For lower effective population sizes (Figure 3: 500; Figure 4: 250) the TDR decreased (illustrated for the most stringent cut-off used, i.e. 100 lowest *P*-values denoted as significant). While the decreasing population size did decrease TDR, this did not alter the main effects described above.

Decreasing the stringency had larger effects on TDR, which decreases in general with lower stringency. This demonstrates that the simulated "selection" alleles are generally among the alleles with the lowest *P*-values and that more lenient cut-offs results in higher false discovery rates. The decrease in TDR resulted in equal TDRs for initial allele frequencies 0.2 – 0.5, and for higher selection intensities when 250 (Figure 5) and 500 (Figure 6) loci are denoted as significant. Furthermore, with the lowest simulated stringency (500) “Age-at-reproduction” showed an equal TDR as

“Developmental diet” at a high selection intensity (Figure 6C) and GLMM had a higher TDR compared to GLM at lower initial allele frequencies (0.2-0.5, Figure 6D) while in simulations with higher stringencies this either was not the case, or this would be the case for 0.5 initial allele frequency only (for instance at lower population sizes, see Figure 4).

The relationship between P-values and dAF

Different directions of selection lead to allele frequency changes between populations. Given that higher intensities should lead to larger allele frequency differences, dAF should be a good predictor for selection. Therefore, scatterplots for dAF and *P*-values (- log10 transformed) were examined for the four tested models and for each contrast (Figure 7). These scatterplots indicate that a clear relationship exists between dAF and *P*-value for GLM. For GLMM a very broad distribution of dAF can result in low *P*-values, which is consistent with the fact that GLMM weighs variation between replicates within treatments. This indicates that GLMM is especially sensitive to variation among replicate populations, irrespective of dAF. Similarly, for AOV and QGLM the distributions of dAF that relate to lower *P*-values fan out, but interestingly for both these models a subset of loci with low dAF leads to a very low *P*-value, especially for “Developmental Diet” and “Age-at-Reproduction”, which is a pattern that is difficult to reconcile with a biologically sensible prediction of the response to selection. To quantify these relationships a Spearman correlation was fitted for these 12 relationships (four models x three contrasts), which also indicated that the GLM has the highest correlation coefficients for all three contrasts.

Correlation of P-values of the four models

To gain insight into whether highly significantly tested loci in one test would be similar to those in other tested we first plotted the pairwise correlations of for “Developmental Diet” (Figure 8), “Age-at-Reproduction” (Figure 9) and “Interaction” (Figure 10). While generally speaking the shapes of the correlation plots are positive, these plots indicate that the most significant loci for a test would be unlikely to be the most significant in another test (also see the Venn diagrams in Figure 11 and Table 2). Given that only alleles with the lowest *P*-values would normally be considered candidate alleles, this finding indicates that the choice of statistical model will have a major impact on the SNPs that are considered candidate loci.

References

Charlesworth, B., Charlesworth, D. (2010). *Elements of Evolutionary Genetics*. Roberts and Company, Greenwood Village, Colorado.

Jha, A.R., Miles, C.M., Lippert, N.R., Brown, C.D., White, K.P., Kreitman, M. (2015). Whole genome resequencing of experimental populations reveals polygenic basis of egg size variation in *Drosophila melanogaster. Mol Biol Evol*. 32:2616-2632.

Jha, A.R., Zhou, D., Brown, C.D., Kreitman, M., Haddad, G.G., White, K.P. (2016). Shared Genetic Signals of Hypoxia Adaptation in *Drosophila* and in High-Altitude Human Populations. *Mol Biol Evol*. 33:501-517.

Jonas, A., Thomas, T., Kosiol, C., Schlötterer, C. (2017). Estimating the effective population size from temporal allele frequency changes in experimental evolution. *Genetics* 204:723-735.

Kelly, J.K., Hughes, K.A. (2019). Pervasive linked selection and intermediate-frequency alleles are implicated in an evolve-and-resequence experiment of *Drosophila simulans*. *Genetics* 211:943-961.

Kelly, J.K., Koseva, B., Mojica, J.P. (2013). The genomic signal of partial sweeps in Mimulus guttatus. *Genome Biol Evol.* 5:1457-1469.

Lynch, M., Bost, D., Wilson, S., Maruki, T., Harris, S. (2014). Population-genetic inference from Pooled-Sequencing data. *Genome Biol Evol*. 6: 1210-1218.

Martins, N.E., Faria, V.G., Nolte, V., Schlötterer, C., Teixeira, L. (2014). Host adaptation to viruses relies on few genes with different cross-resistance properties. *Proc Natl Acad Sci USA*. 111:5938–5943.

Wiberg, R.A.W., Gaggiotti, O.E., Morrissey, M.B., Ritchie, M.G. (2017). Identifying consistent allele frequency differences in studies of stratified populations. *Methods Ecol Evol*. 8:1899-1909.

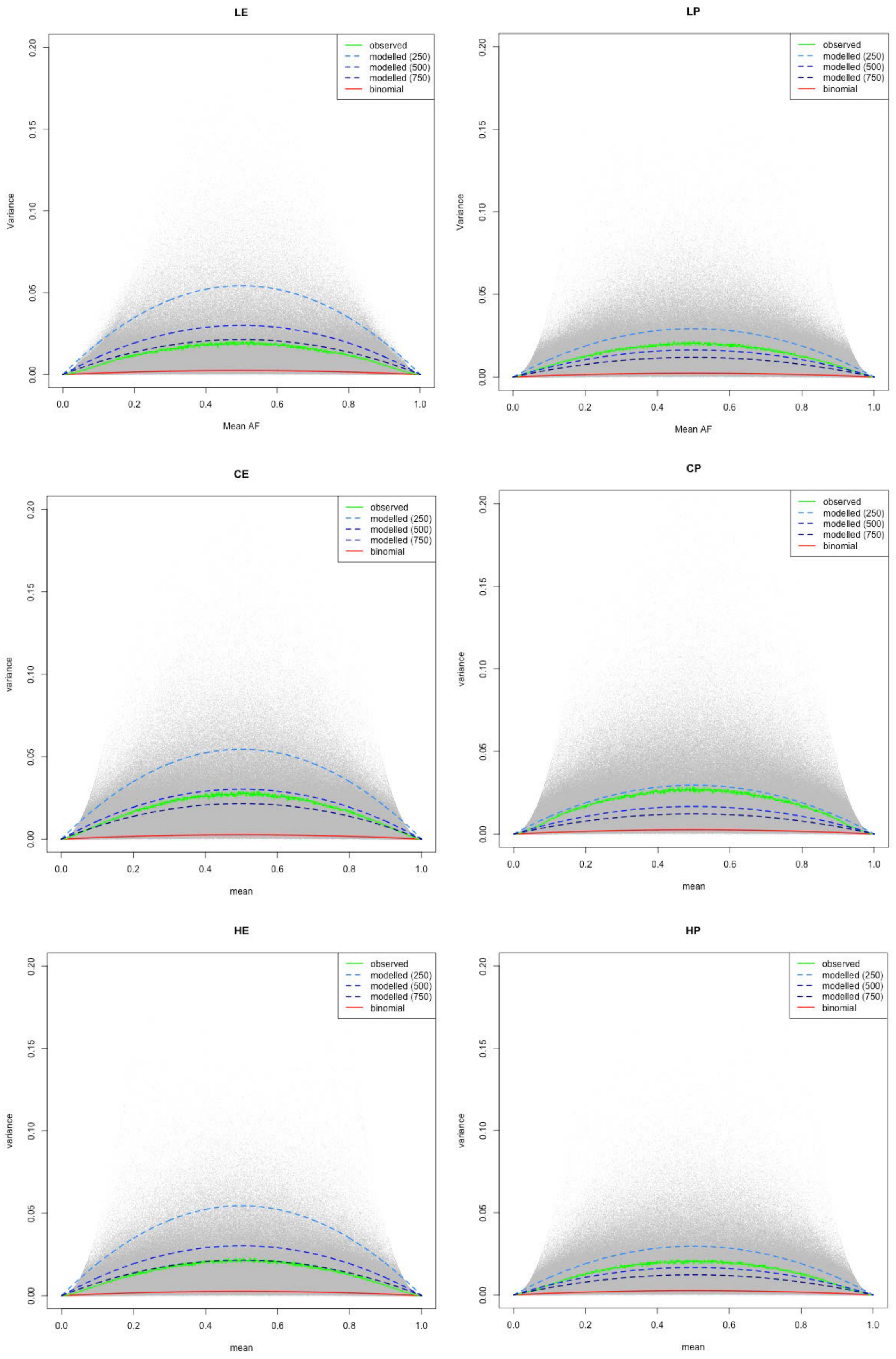

Figure 1. Overdispersion for each of the six regime combinations. Each panel shows the mean allele frequency (x-axis) and the variance (y-axis) of the four replicate populations per regime combination of our real dataset. The green line shows the average variance, taken over non-overlapping windows of 0.01 in allele frequency. The upper, middle and lower dashed blue lines demonstrate the average variance of the simulated datasets with effective populations sizes of 250, 500 and 750 individuals, respectively. The red line demonstrates the variance expected for a binomial distribution.

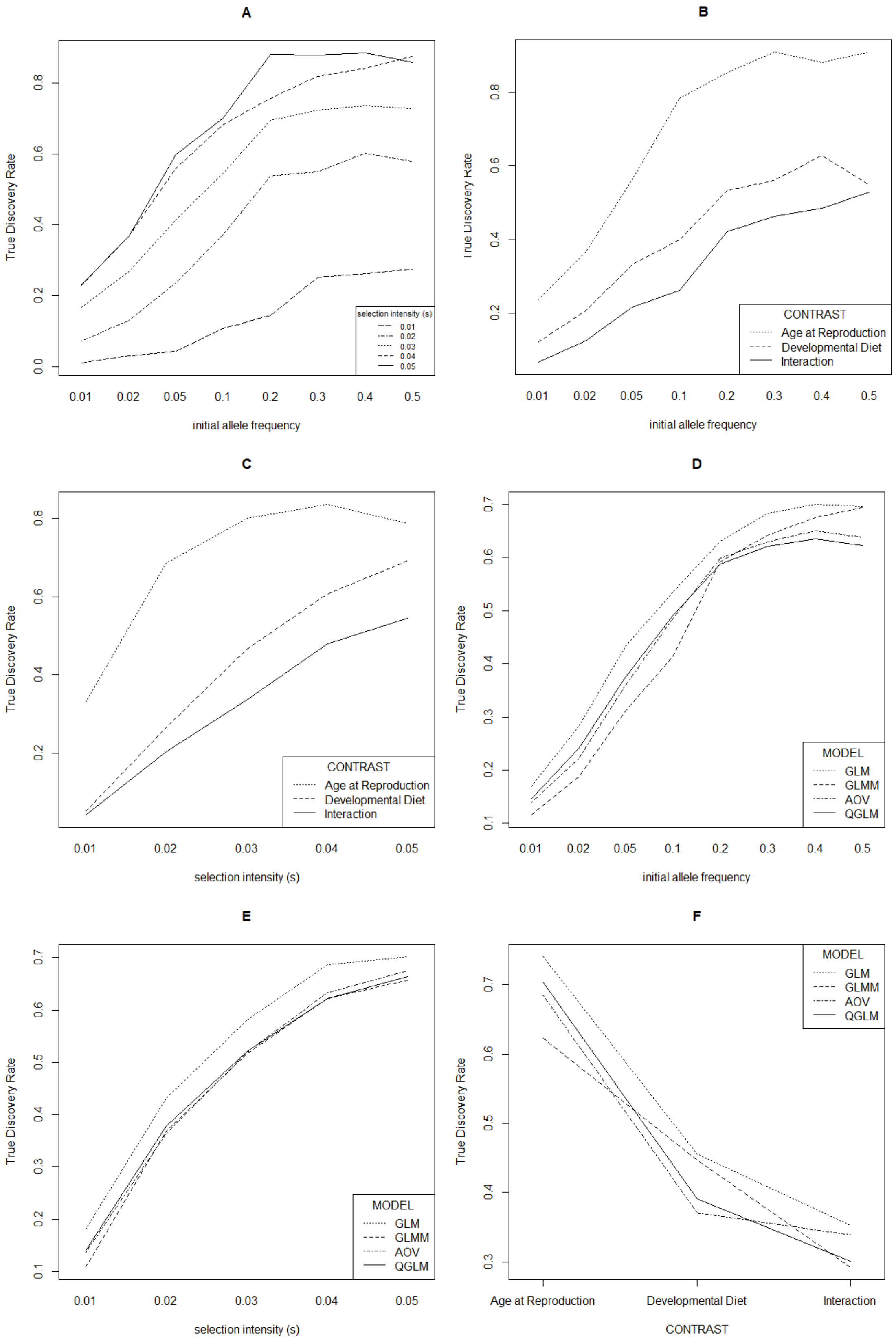

Figure 2. Effects of parameters in the model on TDR (y-axis) for effective population size = 750 and 100 SNPs called significant. Panels A, B and C show the averaged results of all four models with respect to the effects of (A) initial allele frequency (x-axis) and selection intensity (indicated by 5 line types), (B) initial allele frequency (x-axis) and contrast, and (C) selection intensity (x-axis) and contrast. Panels D, E and F show the performance of the four models separately for (D) initial allele frequency, (E) selection intensity and (F) contrast.

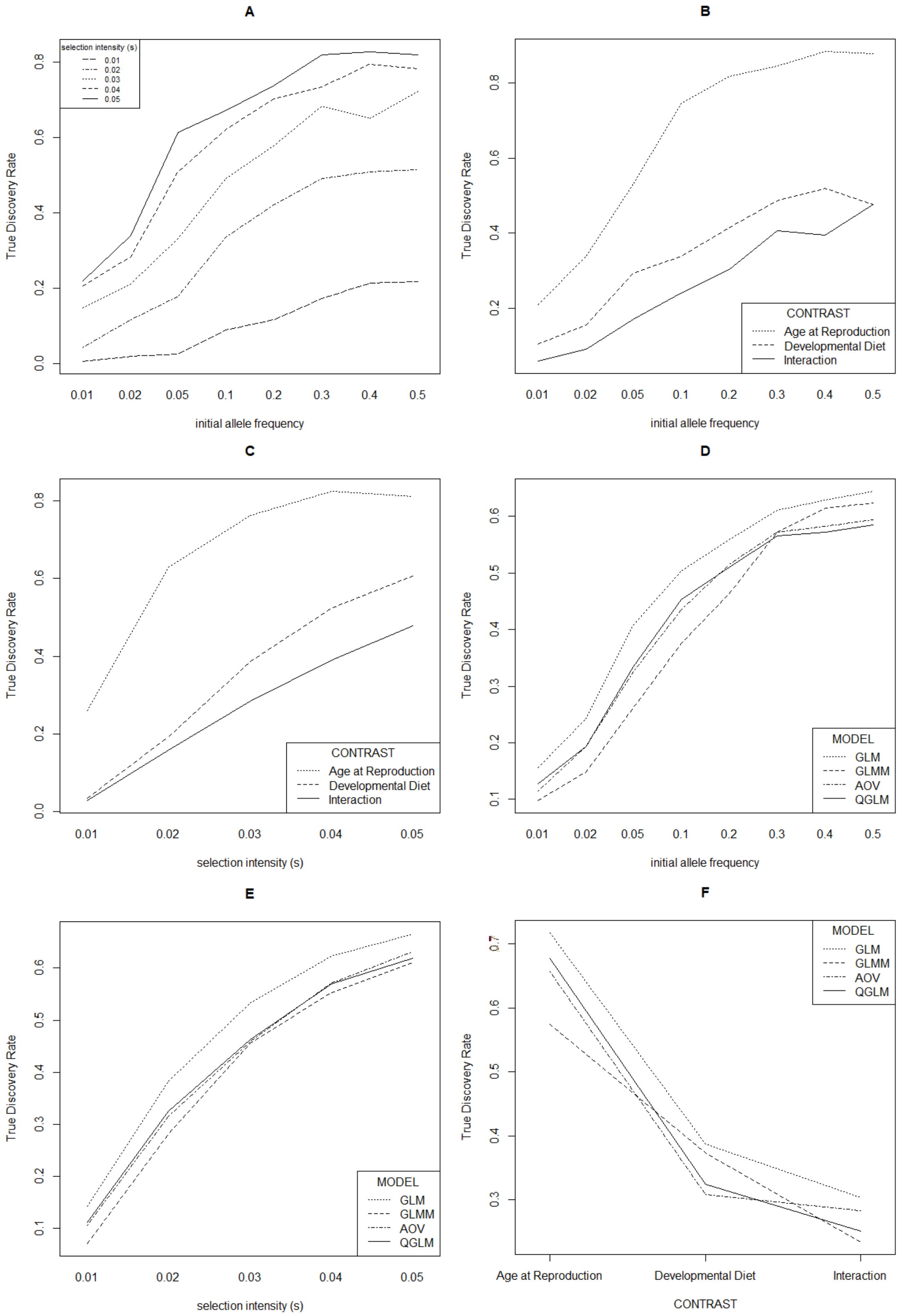

Figure 3. Effects of parameters in the model on TDR (y-axis) for effective population size = 500 and 100 SNPs called significantly. Panels A, B and C show the averaged results of all four models with respect to the effects of (A) initial allele frequency (x-axis) and selection intensity (indicated by 5 line types), (B) initial allele frequency (x-axis) and contrast, and (C) selection intensity (x-axis) and contrast. Panels D, E and F show the performance of the four models separately for (D) initial allele frequency, (E) selection intensity and (F) contrast.

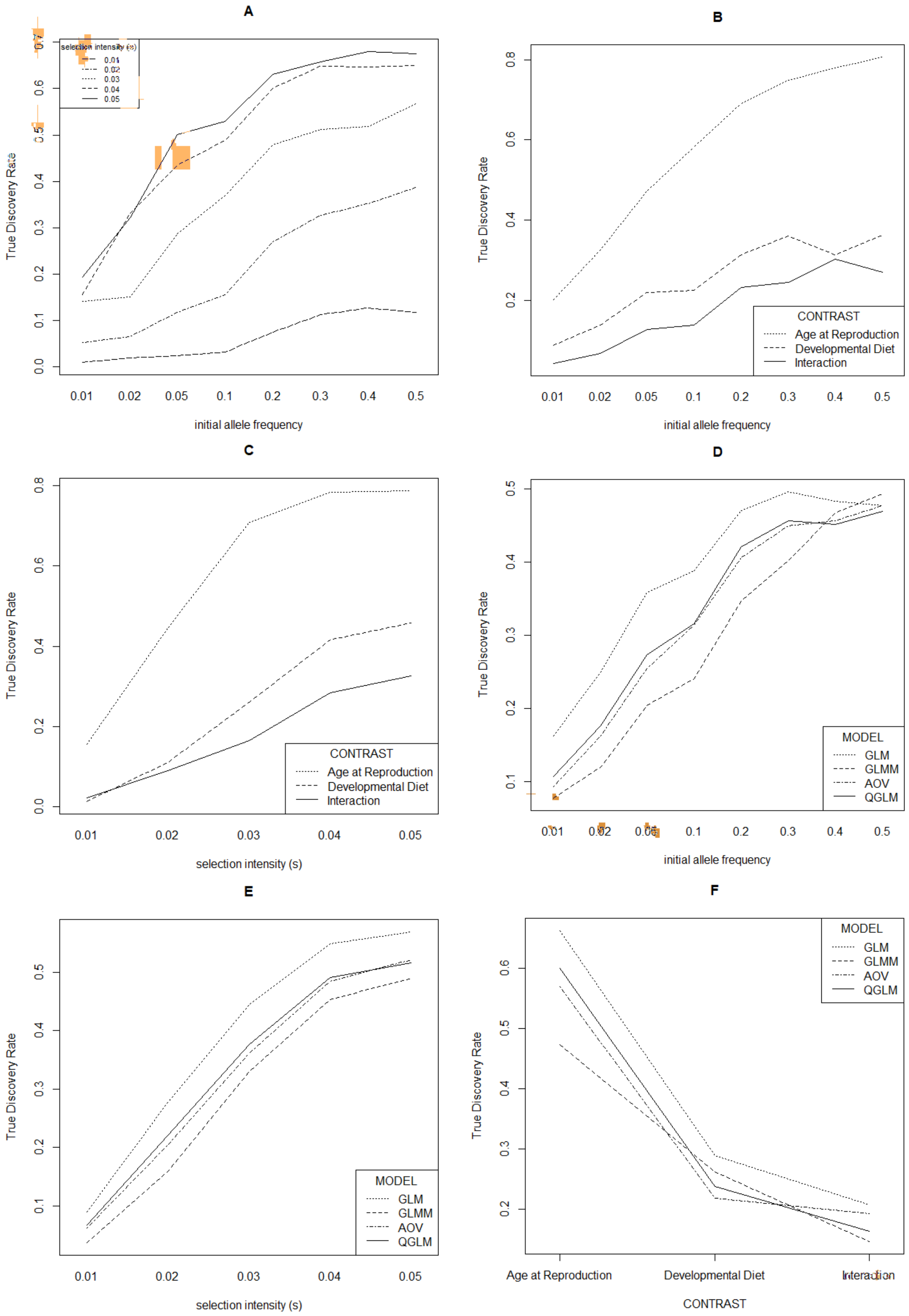

Figure 4. Effects of parameters in the model on TDR (y-axis) for effective population size = 250 and 100 SNPs called significant. Panels A, B and C show the averaged results of all four models with respect to the effects of (A) initial allele frequency (x-axis) and selection intensity (indicated by 5 line types), (B) initial allele frequency (x-axis) and contrast, and (C) selection intensity (x-axis) and contrast. Panels D, E and F show the performance of the four models separately for (D) initial allele frequency, (E) selection intensity and (F) contrast.

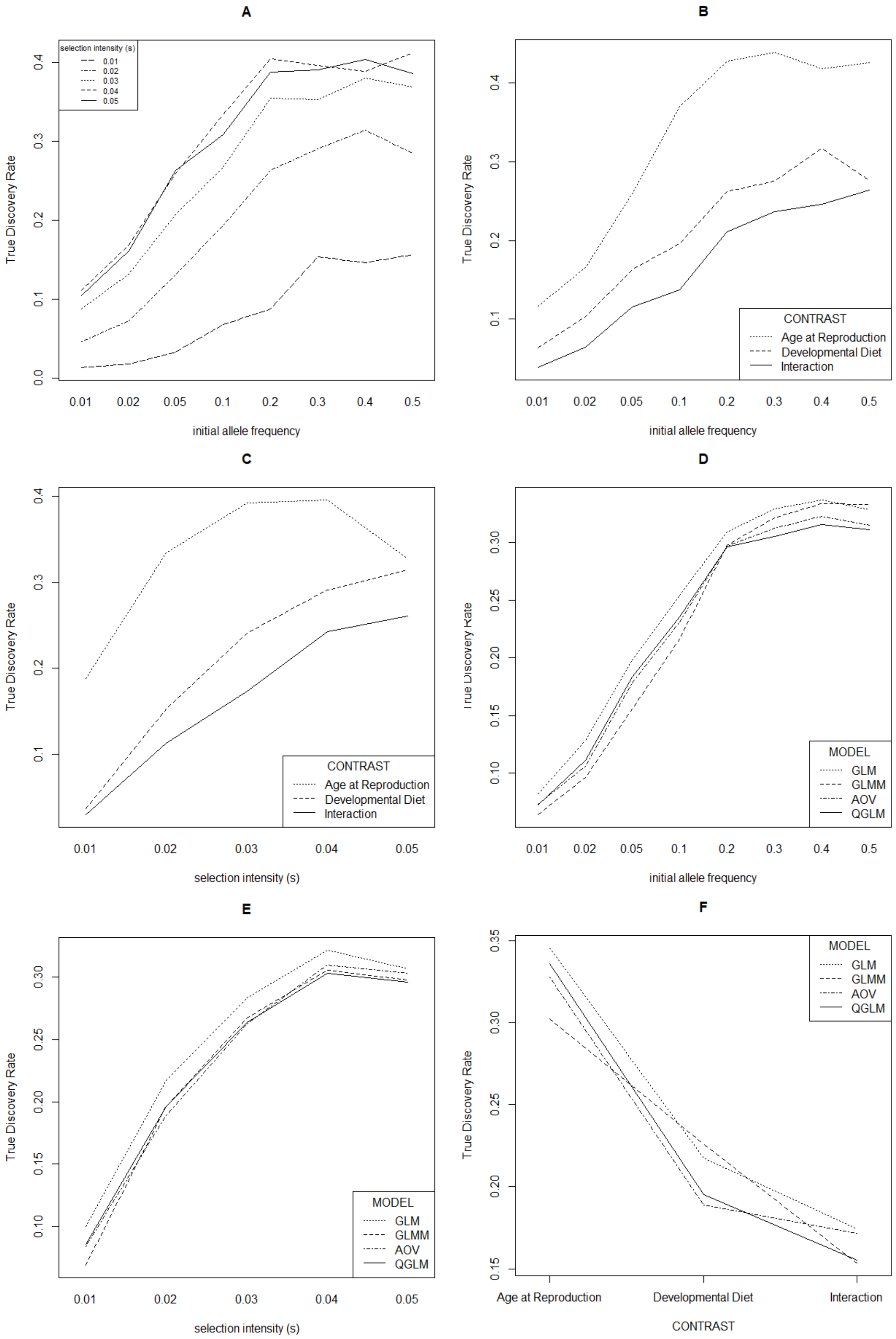

Figure 5. Effects of parameters in the model on TDR (y-axis) for effective population size = 750 and 250 SNPs called significant. Panels A, B and C show the averaged results of all four models with respect to the effects of (A) initial allele frequency (x-axis) and selection intensity (indicated by 5 line types), (B) initial allele frequency (x-axis) and contrast, and (C) selection intensity (x-axis) and contrast. Panels D, E and F show the performance of the four models separately for (D) initial allele frequency, (E) selection intensity and (F) contrast.

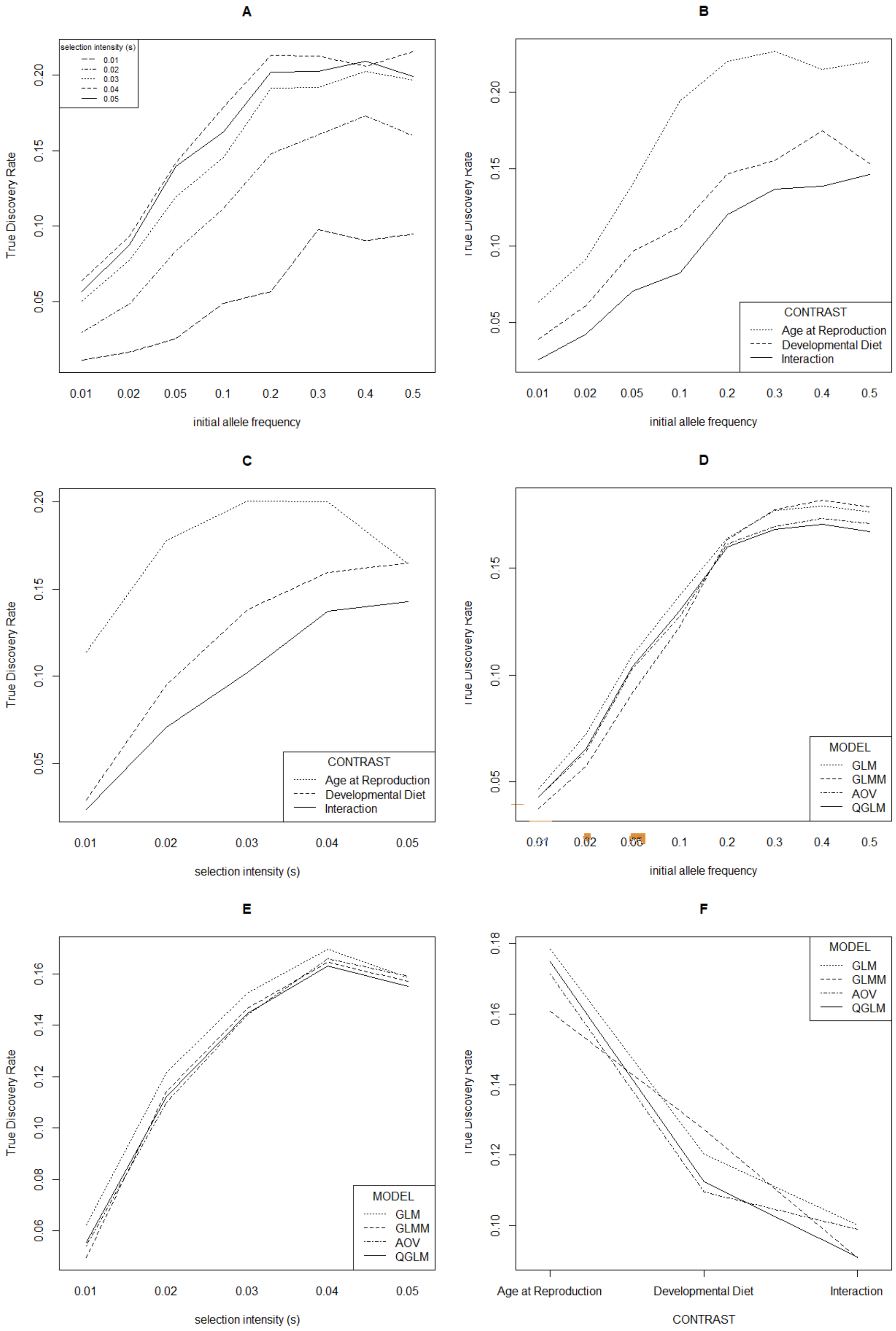

Figure 6. Effects of parameters in the model on TDR (y-axis) for effective population size = 750 and 500 SNPs called significant. Panels A, B and C show the averaged results of all four models with respect to the effects of (A) initial allele frequency (x-axis) and selection intensity (indicated by 5 line types), (B) initial allele frequency (x-axis) and contrast, and (C) selection intensity (x-axis) and contrast. Panels D, E and F show the performance of the four models separately for (D) initial allele frequency, (E) selection intensity and (F) contrast.

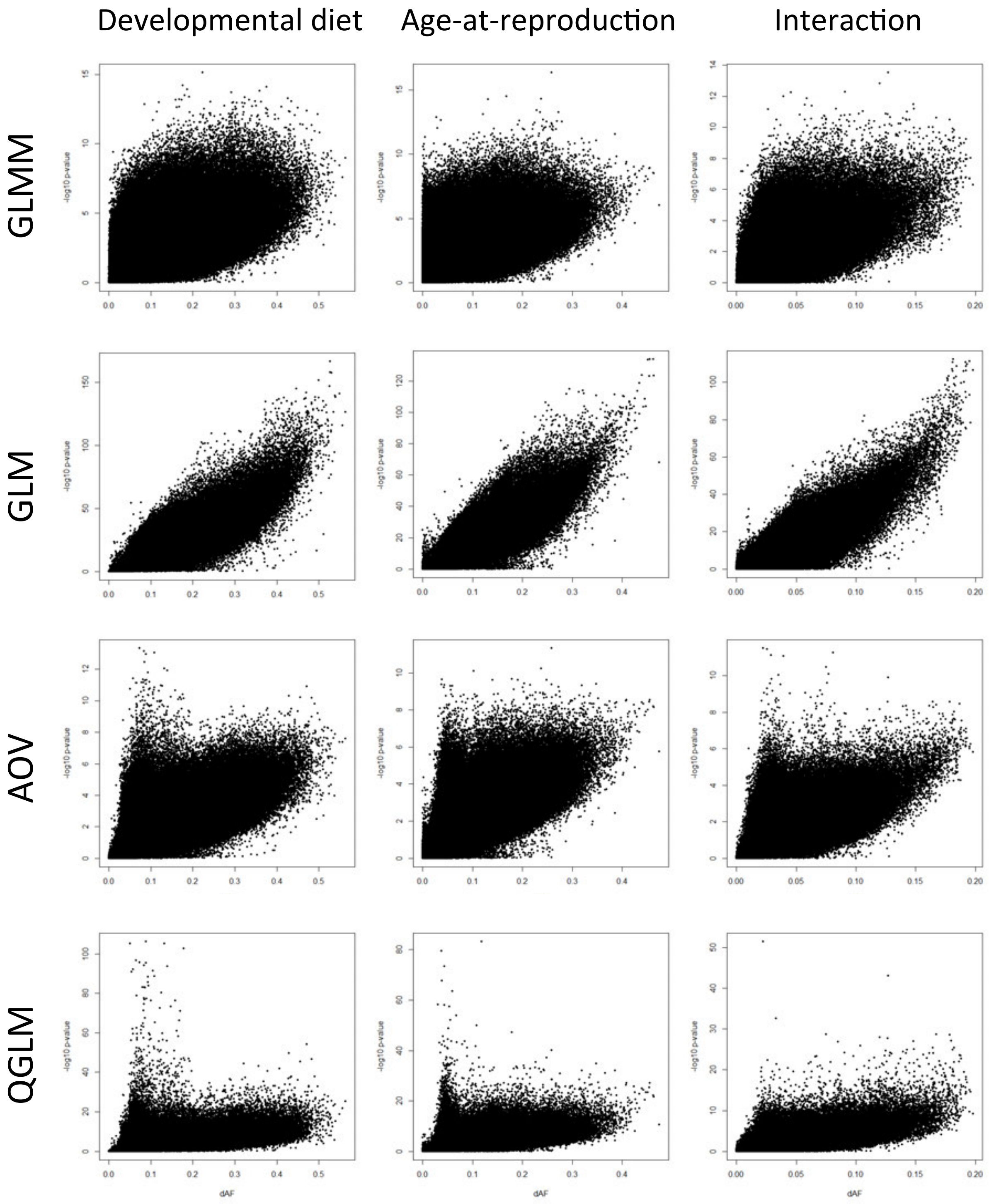

Figure 7. Scatterplots of absolute dAF and *P*-value (-10 log transformed). Every dot represents dAF (x-axis) and the *P*-value (y-axis) for a SNP. The values represent raw *P*-values. Separate plots for each of the three factors ("developmental diet", "age-at-reproduction", and "interaction") and the four models are shown. The dAF for the three factors as shown here is calculated as follows:

Developmental diet: dAF(Dev) =max(|mean(L) – mean(C)|, |mean(P) – mean(H)|, |mean(H) – mean(C)|); Age-at-reproduction: dAF(Rep) = |mean(E) – mean(P)|; Interaction: dAF(Int) =Σ Σ |mean(I,J)-mean(I)-mean(J)+M| *(I is different larval diets [L/C/H], J different reproduction regimes [E/P])/6

Table 1. Values of rho correlation coefficients (resulting from Spearman correlation tests) of the *P*-values and dAF for the four models tested and the three contrasts.

Rho correlation coefficients	Developmental Diet	Age at Reproduction	Interaction
GLMM	0.503	0.518	0.631
GLM	0.812	0.892	0.782
AOV	0.704	0.825	0.720
QGLM	0.661	0.819	0.658

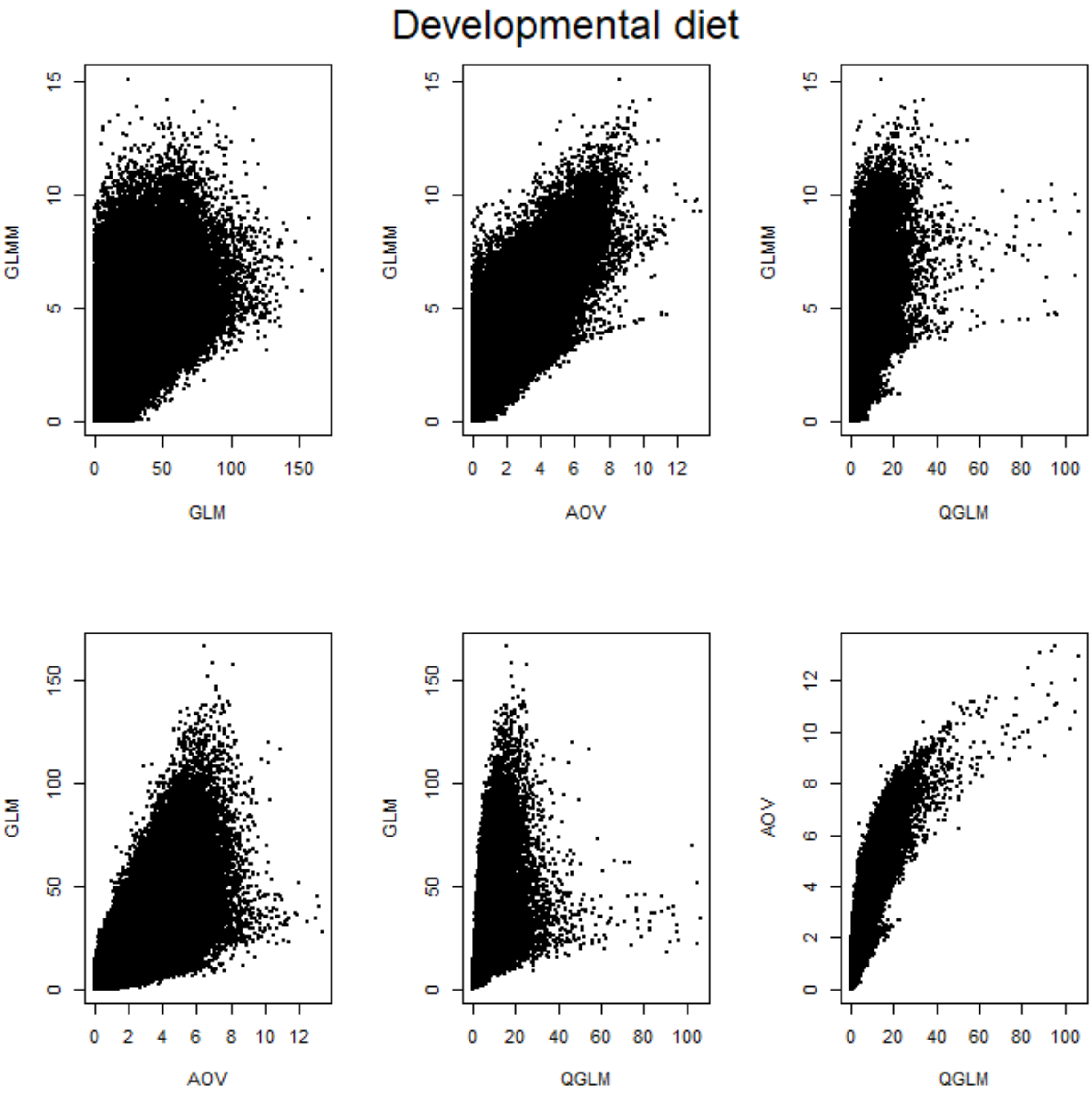

Figure 8. Pairwise *P*-values (-10 log transformed) for "Developmental Diet". Every dot represents the *P*-value for a SNP for two different models indicated on the x and y axis. The values represent raw *P*-values.

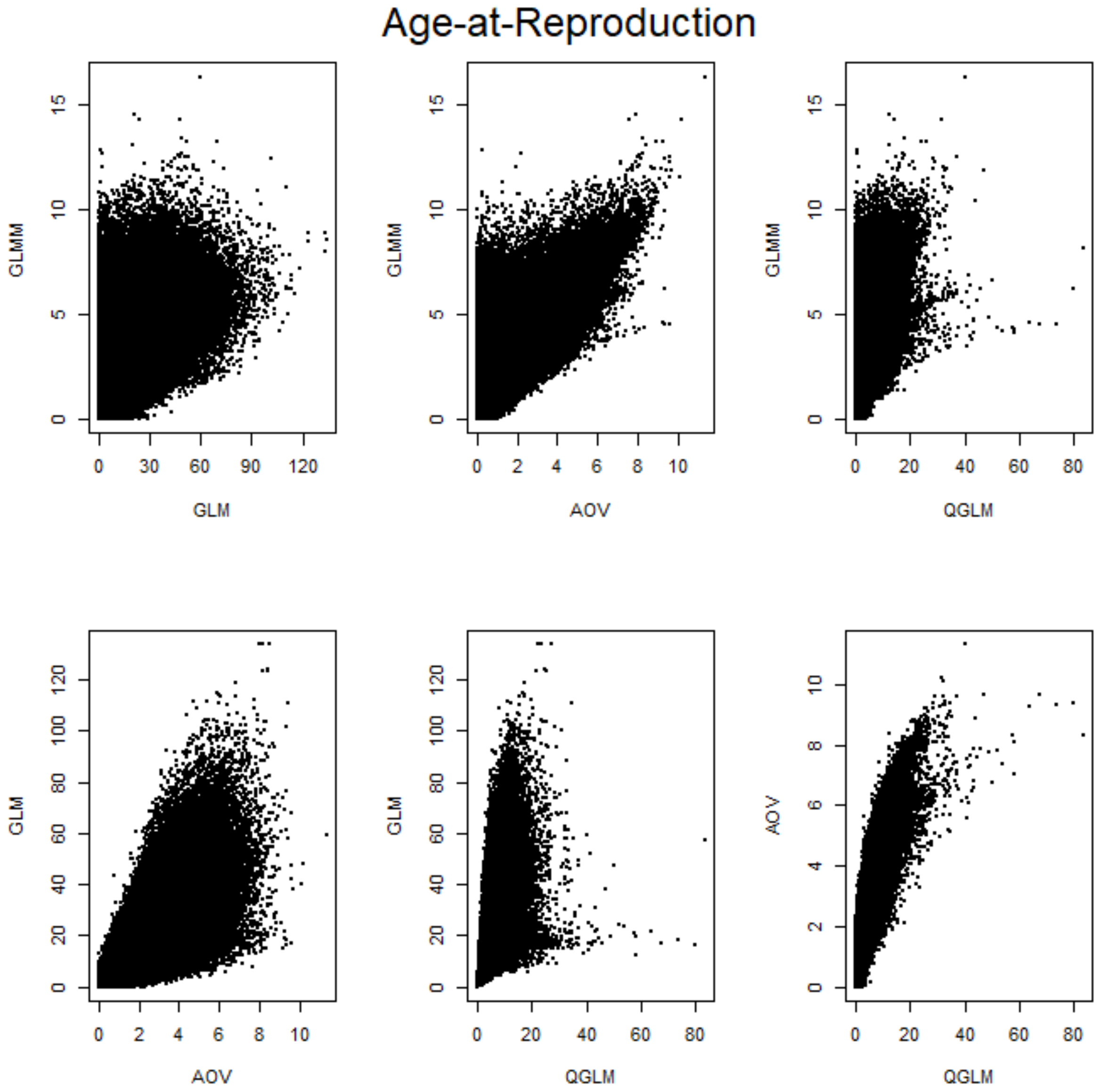

Figure 9. Pairwise *P*-values (-10 log transformed) for "Age-at-reproduction". Every dot represents the *P*-value for a SNP for two different models indicated on the x and y axis. The values represent raw *P*-values.

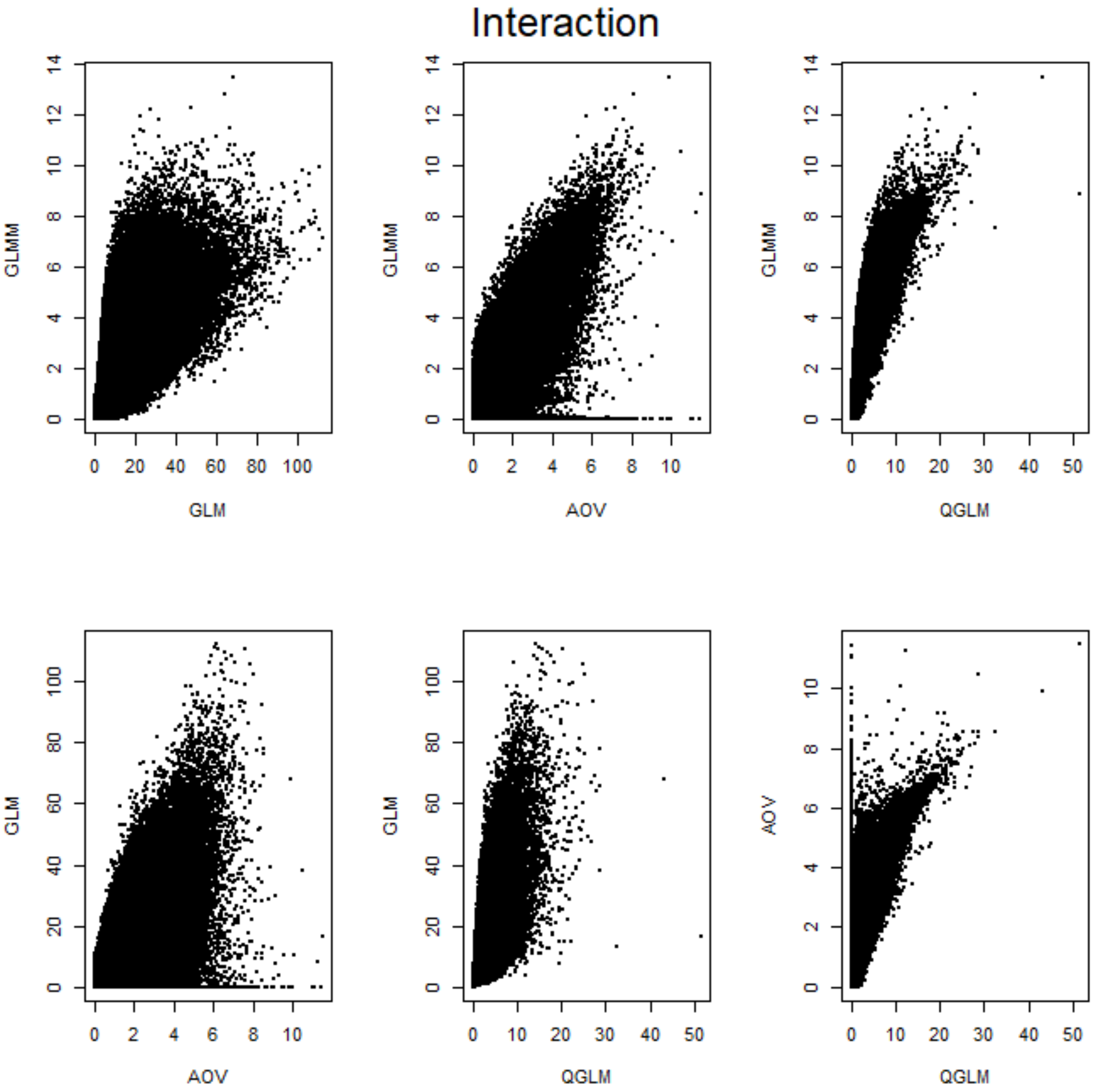

Figure 10. Pairwise *P*-values (-10 log transformed) for "Interaction". Every dot represents the *P*-value for a SNP for two different models indicated by the x and y axis. The values represent raw *P*-values.

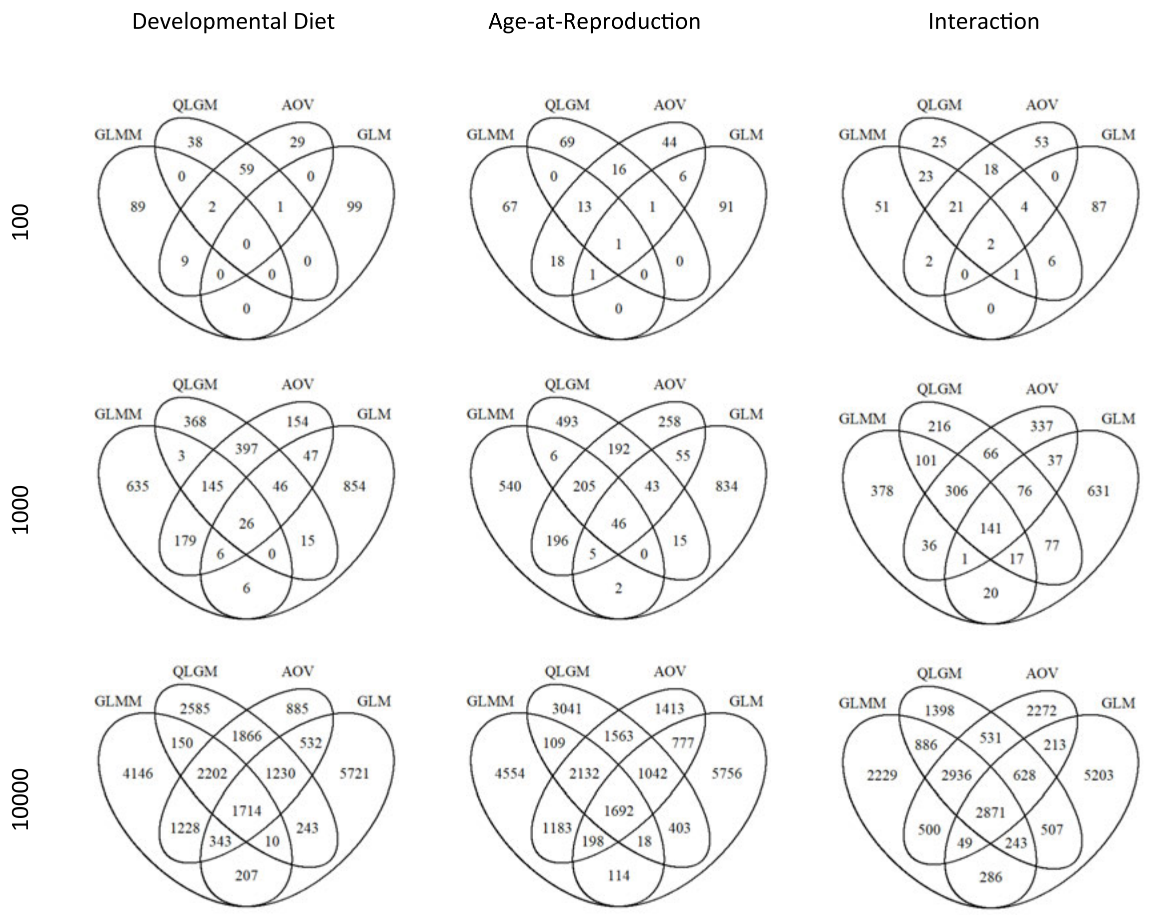

Figure 11. Venn diagrams showing the overlap for the most significant SNPs per model. The overlaps among the 100 (top), 1000 (middle), and 10000 (bottom row) most significant SNPs are shown for the two main factors (developmental diet (left) and age-at-reproduction (middle)) and the interaction (right column).

Table 2. Pairwise overlap among the four models in percentage. The overlaps among the 100, 1000, and 10000 most significant SNPs are shown (same data as in Figure 12).

A. Developmental diet		GLM	GLMM	QGLM	AOV
GLM	top-100	100	0.0	1.0	0.0
	top-1000	100	3.8	8.7	12.5
	top-10000	100	22.7	32.0	38.2
GLMM	top-100	0.0	100	2.0	11.0
	top-1000	3.8	100	17.4	35.6
	top-10000	22.7	100	40.8	54.9
QGLM	top-100	1.0	2.0	100	62.0
	top-1000	8.7	17.4	100	61.4
	top-10000	32.0	40.8	100	70.1
AOV	top-100	0.0	11.0	62.0	100
	top-1000	12.5	35.6	61.4	100
	top-10000	38.2	54.9	70.1	100

B. Age-at-reproduction		GLM	GLMM	QGLM	AOV
GLM	top-100	100	2.0	2.0	9.0
	top-1000	100	5.3	10.4	19.7
	top-10000	100	20.2	31.6	37.1
GLMM	top-100	2.0	100	14.0	33.0
	top-1000	5.3	100	25.7	45.2
	top-10000	20.2	100	39.5	52.1
QGLM	top-100	2.0	14.0	100	31.0
	top-1000	10.4	25.7	100	48.6
	top-10000	31.6	39.5	100	64.3
AOV	top-100	9.0	33.0	31.0	100
	top-1000	19.7	45.2	48.6	100
	top-10000	37.1	52.1	64.3	100

C. Interaction		GLM	GLMM	QGLM	AOV
GLM	top-100	100	3.0	13.0	6.0
	top-1000	100	17.9	31.1	25.5
	top-10000	100	34.5	42.5	37.6
GLMM	top-100	3.0	100	47.0	25.0
	top-1000	17.9	100	56.5	48.4
	top-10000	34.5	100	69.4	63.6
QGLM	top-100	13.0	47.0	100	45.0
	top-1000	31.1	56.5	100	58.9
	top-10000	42.5	69.4	100	69.7
AOV	top-100	6.0	25.0	45.0	100
	top-1000	25.5	48.4	58.9	100
	top-10000	37.6	63.6	69.7	100

Supplementary Result S3: *P*-value distribution of GLM

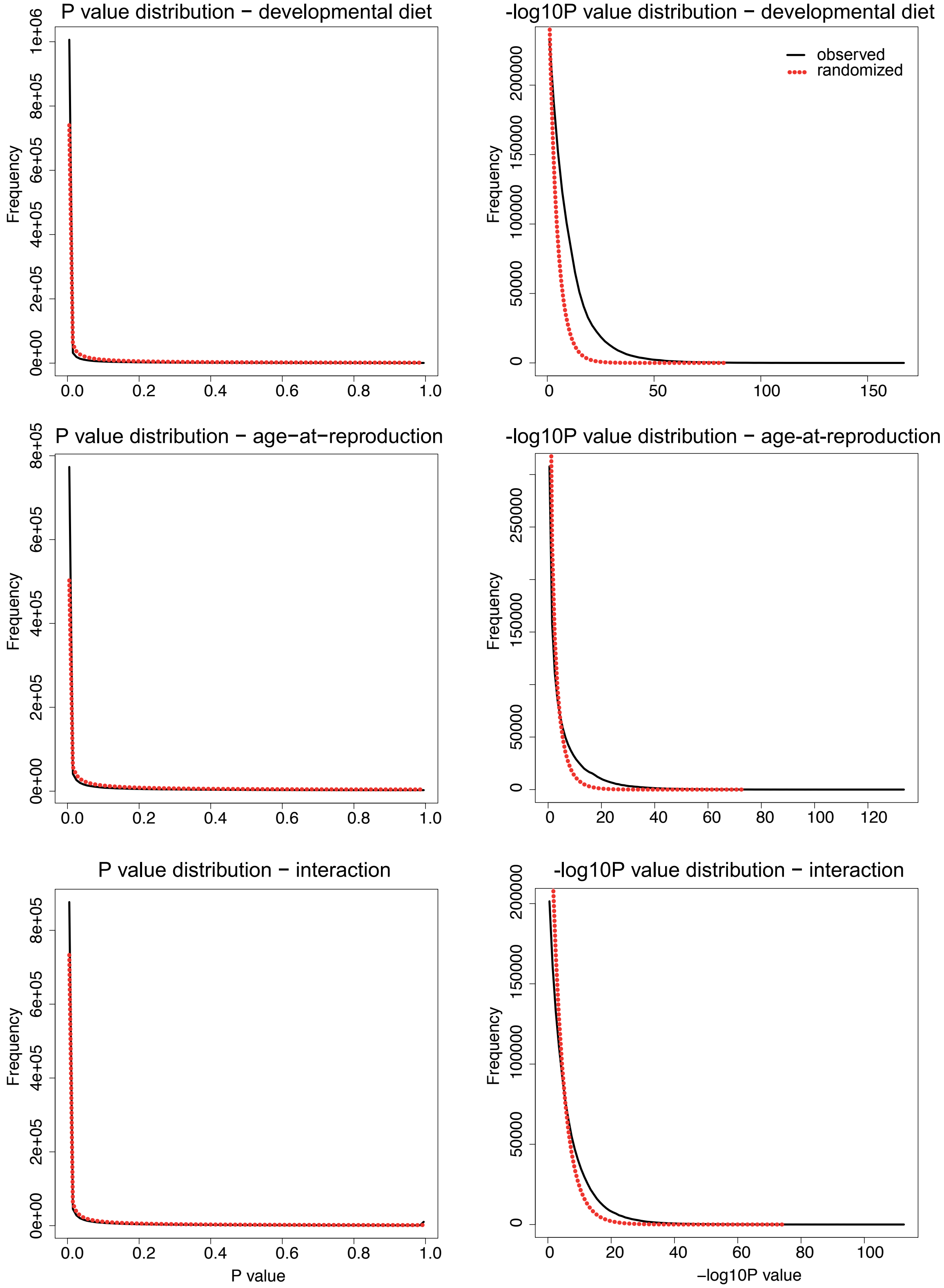

Figure 1: *P*-value (left) and -log10*P*-value (right) distribution plots for all SNPs analyzed with binomial GLMs (solid, black: observed data; dotted, red: average of the 10 randomized datasets). Distribution plots for the two main factors (Developmental diet (top) and Age-at-reproduction (middle)) and the interaction (bottom) are shown.

Table 1: The lowest *P*-values for the two main factors and their interaction for the observed dataset and each of the ten permuted datasets.

	Diet	Reproduction	Interaction
**Observed**	**2.40E-167**	**1.37E-134**	**5.20E-113**
P1	1.15E-60	8.19E-63	8.03E-75
P2	1.31E-82	3.69E-50	1.18E-76
P3	6.92E-58	3.61E-73	9.59E-56
P4	8.84E-63	1.23E-57	1.10E-65
P5	1.28E-65	3.02E-52	2.50E-63
P6	4.33E-61	1.14E-53	2.47E-51
P7	3.42E-59	1.18E-48	1.20E-65
P8	1.06E-57	9.60E-56	3.71E-65
P9	4.92E-51	1.18E-63	4.86E-76
P10	1.73E-84	2.34E-74	1.96E-59

Supplementary Result S4: Allele frequency differentiation

As a summary of the allele frequency differentiation (ΔAF) in response to the two EE regimes, we plotted the average ΔAF per regime or regime combination for all SNPs (Figure 1). The maximum ΔAF was 0.56 for developmental diet, with a median of 0.08, and 0.48 for age-at-reproduction, with a median of 0.05. This indicates that genomic differentiation has occurred in response to both EE regimes. Both the median (0.19) and maximum ΔAF (0.88) among the six EE regime combinations were, however, higher than either of the two separate regimes, which suggests that the EE regimes have interacted at the genomic level. Despite the strong allele differentiation observed, there were no cases in which the diverged allele had reached fixation, which suggests that partial soft sweeps took place. This is a common observation for E&R studies in *Drosophila*, especially in case of quantitative traits, such as aging or life history traits. Adaptation of these traits has been found to depend on multiple loci with small effect sizes in general, which may be explained by pleiotropy, epistasis, dominance or frequency-dependent selection among others (Burke *et al*. 2010; Burke and Long 2012; Long *et al*. 2015). We also plotted the allele frequencies of all SNPs on a Manhattan plot as an indication of the regions that have diverged between the selective regimes (Figure 2, significant SNPs as detected by GLM analyses highlighted in red).

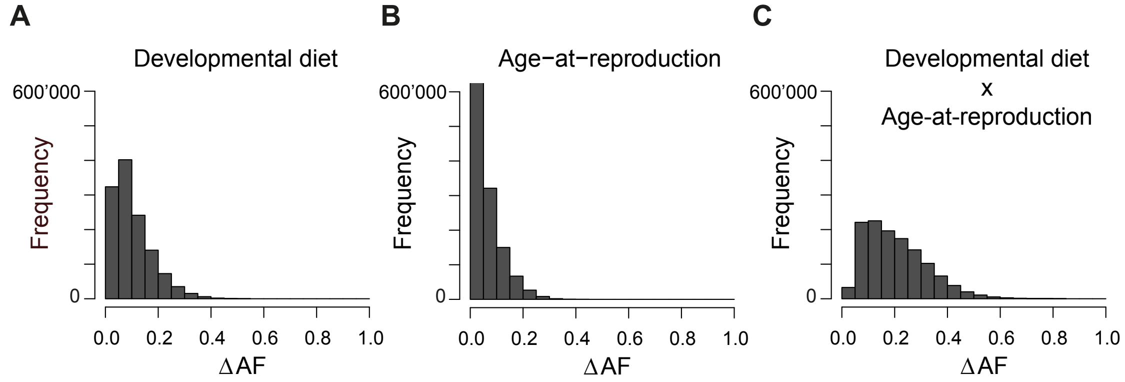

Figure 1: Overview of allele frequency differentiation (ΔAF). Plots of allele frequency differentiation (ΔAF) in response to (A) developmental diet (the maximum pairwise differentiation among the three diets is shown) and (B) age-at-reproduction. (C) The maximum pairwise differentiation among the six EE regime combinations is larger than either of the two separate EE regimes, which suggests an interaction of the regimes in SNP allele differentiation.

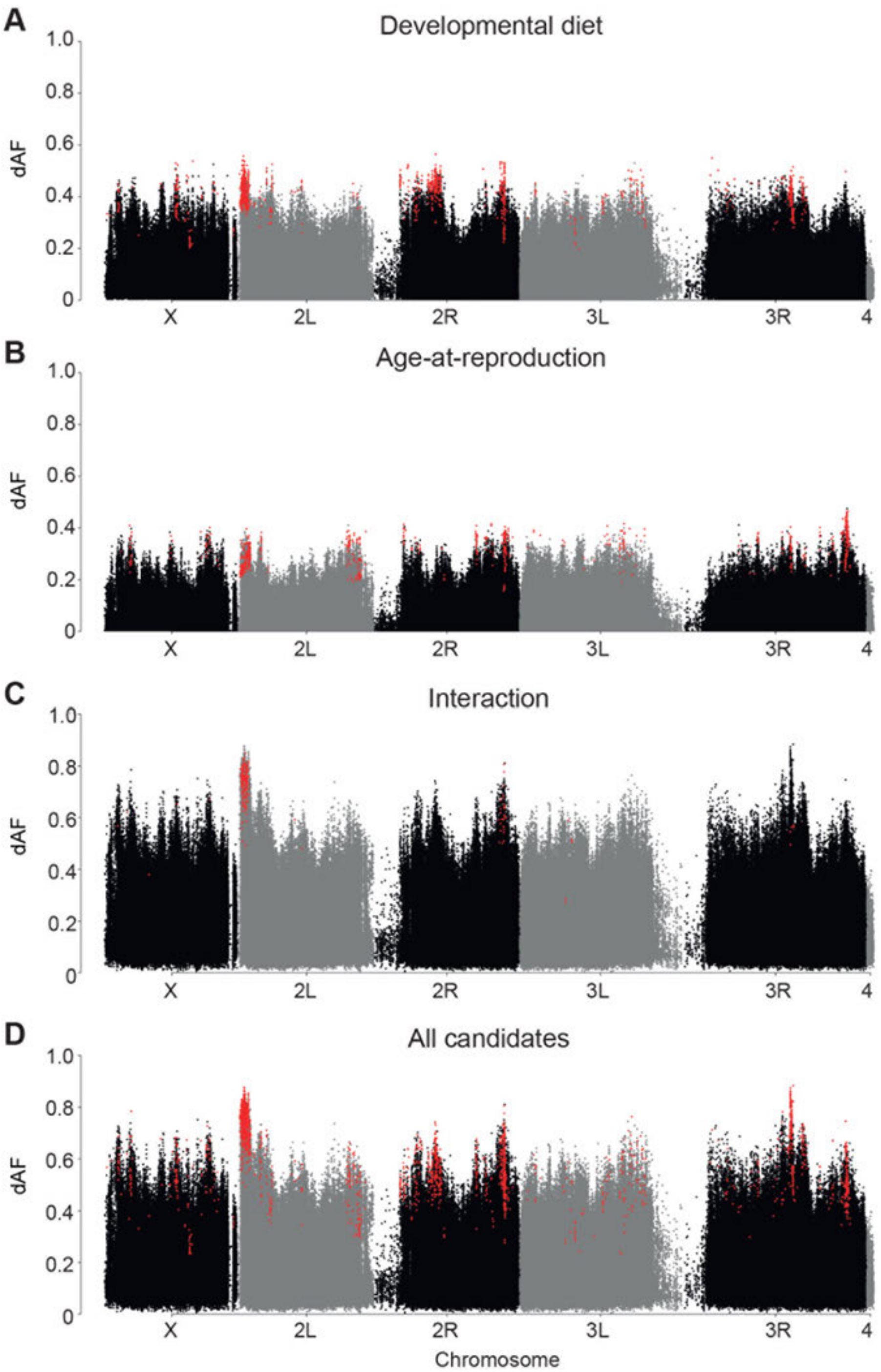

Figure 2: Manhattan plots of allele frequency differences. The Manhattan plots indicate regions of SNP allele frequency differentiation across the genome for the two main factors, (A) 'developmental diet' (the maximum pairwise differentiation among the average allele frequency of the three diets is shown) and (B) 'age at reproduction' (i.e. the difference between the average allele frequency of E and P populations). Significantly differentiated SNPs with a FDR<0.0000008 as detected by binomial GLM analyses are indicated in red. (C-D) The maximum pairwise differentiation among the six EE regime combinations tends to be larger than either of the two separate EE regimes and indicates a combination of main effects and interaction between the two regimes; on (C) the 'interaction' loci as indicated by GLM are highlighted, whereas on (D) all 2252 significant loci are highlighted.

As a second approach to visualize overall patterns of allele frequency differentiation, which supplements the clustering tree presented in figure 2 of the main text, we performed a PCA analysis on the allele frequencies of all SNPs (*prcomp* function in R) (Figure 3). This analysis demonstrates that PCA axis 1 separates the "LE" population from all other populations. Axis 2 separates early from late reproducing populations. Axes 1 and 4 together separate the three different larval diets.

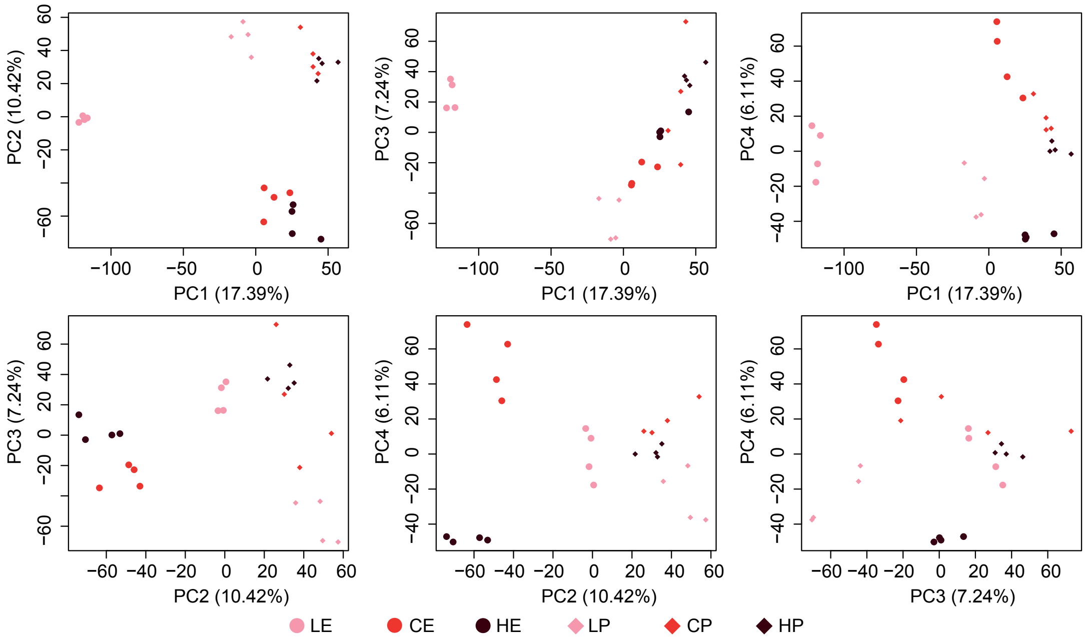

Figure 3: PCA analysis on the allele frequencies of all SNPs. Plots of the first four axes of the PCA analysis are shown.

References

Burke, M.K., Dunham, J.P., Shahrestani, P., Thornton, K.R., Rose, M.R., Long, A.D. (2010). Genome-wide analysis of a long-term evolution experiment with *Drosophila*. *Nature* 467:587-U111.

Burke, M.K., Long, A.D. (2012). What paths do advantageous alleles take during short-term evolutionary change? *Mol Ecol*. 21:4913-4916.

Long, A., Liti, G., Luptak, A., Tenaillon, O. (2015). Elucidating the molecular architecture of adaptation via evolve and resequence experiments. *Nat Rev Genet*. 16:567-582.

Supplementary Result S5: Cluster analysis

Inspection of allele frequency differentiation patterns of significant SNPs revealed distinctly different patterns among SNPs significant for "Developmental diet" or "Age-at-reproduction". Importantly, we also observed cases in which SNPs with similar differentiation patterns were considered significant for different factors in the GLM analyses. To quantify the number of distinct patterns in allele frequencies among the significant loci we performed a clustering analysis on the complete set of significant SNPs (n = 2252).

Identification of clusters
The significant SNPs were clustered (*hclust* package in *R*, hierarchical clustering, method = average) using 1 – absolute correlation ("Pearson") as a distance parameter. This procedure groups together loci on which selection has had similar effects with respect to relative allele frequency changes, while the effect on absolute allele frequency divergence might differ between loci. The pairwise distances between SNPs are represented by a clustering tree (Figure 1). To obtain the optimal number of clusters, this clustering tree was cut in 1 to 100 clusters and for each cluster a PCA was performed to obtain the eigenvector (PC1, representing 73.0 - 89.2% of total variation in cluster). Subsequently, the log likelihood of regressions on all loci were summed for analyses with different number of clusters. As an expected value, a chi square distribution was equated to these differences in log likelihoods (α = 0.95, d.f. 2 x number of clusters). The first value (from 2 to 100 clusters) for which the difference in likelihood fell below this χ^2 distributed value was considered the optimal number of clusters, which was 25.

Characterization of allele frequency differentiation in the clusters
To illustrate the variation that is present in the 25 clusters, the PC1 values of all populations, which give an overview of the allele frequency differentiation in each cluster, are shown in Figure 2.

To identify whether these patterns reflected a response to "Developmental diet", "Age-at-reproduction", to both, or an interaction of the two regimes, we performed ANOVAs on the PC1 values, which are normally distributed. In the first ANOVA, 'Developmental diet', 'Age-at-reproduction' and their interaction were analyzed. The *P*-values of this ANOVA on the PC1 values of the clusters with respect to the main effects "Developmental diet" and "Age-at-reproduction", and "Interaction" are shown in Table 1, together with the numbers of SNPs that were significant in the GLM analysis for these three terms. Generally, the most significant term in the ANOVA agrees well with the significant terms in the GLM. For instance, for cluster 1 (44 SNPs) the ANOVA of PC1 indicated the most significant term was "Developmental Diet" and

indeed these 44 SNPs are significant for "Developmental diet" in the GLM as well. One exception is cluster 7 (1112 SNPs) that has overlapping effects for "Development diet", "Age-at-reproduction" and Interaction for both tests.

While the most significant terms aligned well between the ANOVA on PC1 and the GLM outcomes, for some clusters two or three terms were significant. To further evaluate these patterns, we performed a post hoc Tukey-HSD test on the two main effects after the ANOVA. We performed a second ANOVA with the factor 'EE regime combination' (six in total), followed by post hoc Tukey-HSD tests to be able to better investigate the interaction of the two selection regimes. Contrasts were considered significant if P < 1.0502e-4 (Bonferroni correction: 0.05 / [19 comparisons * 25 clusters]) and are indicated by pink shading in Table 2. Some clusters only showed a clear main effect. For instance, cluster 5 (298 SNPs) can be considered an "Age-at-reproduction" cluster, for which the effect of early *versus* late reproduction was significant as a main effect, as well as for all specific early *versus* late contrasts between EE regime combinations. A main effect of "Developmental diet" was seen for cluster 12 (321), for which there is a significant contrast between "L" and the two other developmental diets. On the other hand, Cluster 7 (1112 SNPs) can be annotated as a "LE" cluster, as the LE populations differ significantly from all the others, whereas these other populations do not differ from each other. This indicates an interaction of the two EE regimes.

Table 3 provides an overview of the inferred effects of the EE regimes on the allele frequencies as determined by the ANOVAs and post hoc tests for all clusters. Main effects of "Developmental diet" (clusters 6, 9, 12, 13, 14, 15, 17, 23) and "Age-at-reproduction" (4, 5, 10, 11, 16, 19, 22, 25) are observed for a total of 513 SNPs (222 genes) and 399 SNPs (154 genes), respectively. Clusters 1, 2, 3, 7, 8, 18, 20, 21 and 24 show significant interactions, i.e. 1340 SNPs (346 genes) in total. Of the 1340 interaction loci, 1112 (241 genes, cluster 7) represent cases in which the four LE populations differ from all others. Supplementary tables S1 and S4 provide an overview of these SNPs and genes. Figure 3 provides an overview of the location of the candidate SNPs within each cluster on the genome. This figure shows that loci with similar allele differentiation patterns occur across distant locations of the genome.

Each candidate SNP has been grouped into a single cluster depending on the allele divergence pattern, but SNPs with different divergence patterns can fall into the same genes. This results in overlap in candidate genes associated with "Age-at-reproduction", "Developmental diet", and "Interaction". The overlap is shown in Figure 4 and Table 4. We tested if the overlap between the three sets of genes was significantly higher or lower than expected using

the package "SuperExactTest" (v0.99.4) in R. Bonferroni correction (α = 0.05/43 intersections analysed with the SuperExactTest in total = 0.0012) was applied to account for multiple testing. These analyses show a small (n=8, 2%), but significantly higher overlap than expected by chance between "Age-at-reproduction" and "Develomental diet". The overlap between the two main effect genes and "interaction" genes was also significantly enriched, respectively with 12 (2%) and 27 (5%) overlapping genes with "Age-at-reproduction" and "Developmental diet", respectively.

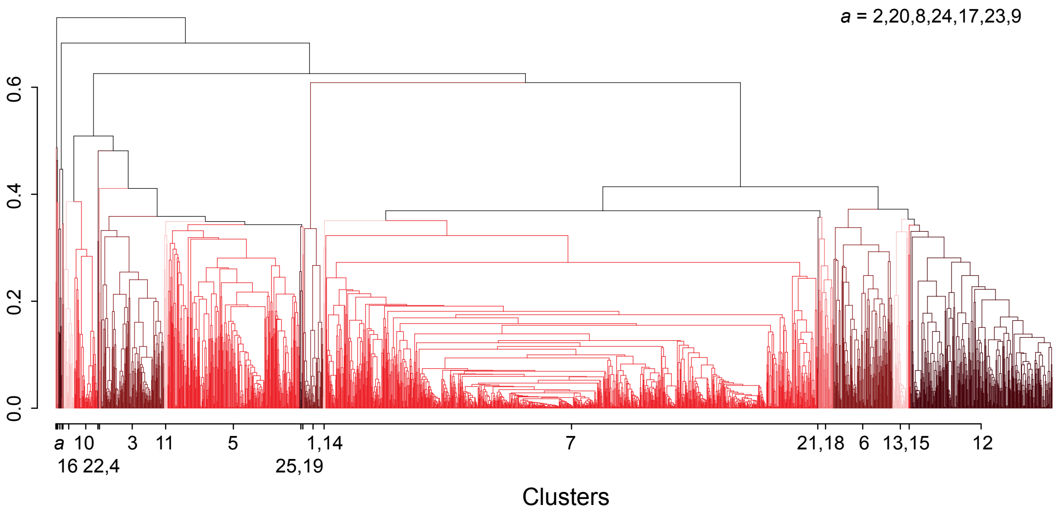

Figure 1: Clustering tree resulting from the hierarchical clustering. The y-axis indicates the average distance between sister clusters. The 25 different clusters are indicated on the x-axis and by different colors.

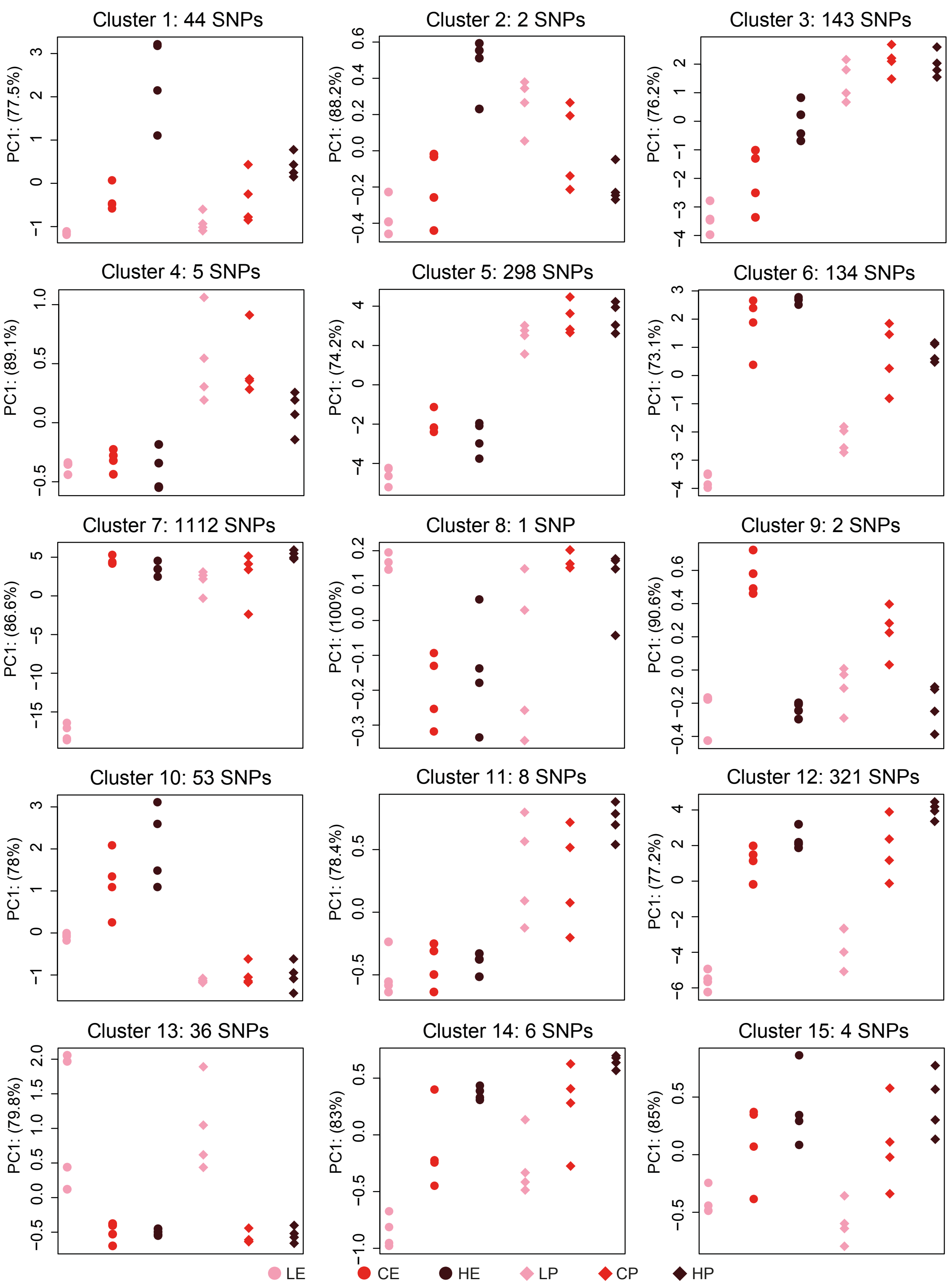

Cluster 1: 44 SNPs
PC1: (77.5%)
Cluster 2: 2 SNPs
PC1: (88.2%)
Cluster 3: 143 SNPs
PC1: (76.2%)
Cluster 4: 5 SNPs
PC1: (89.1%)
Cluster 5: 298 SNPs
PC1: (74.2%)
Cluster 6: 134 SNPs
PC1: (73.1%)
Cluster 7: 1112 SNPs
PC1: (86.6%)
Cluster 8: 1 SNP
PC1: (100%)
Cluster 9: 2 SNPs
PC1: (90.6%)
Cluster 10: 53 SNPs
PC1: (78%)
Cluster 11: 8 SNPs
PC1: (78.4%)
Cluster 12: 321 SNPs
PC1: (77.2%)
Cluster 13: 36 SNPs
PC1: (79.8%)
Cluster 14: 6 SNPs
PC1: (83%)
Cluster 15: 4 SNPs
PC1: (85%)
LE
CE
HE
LP
CP
HP

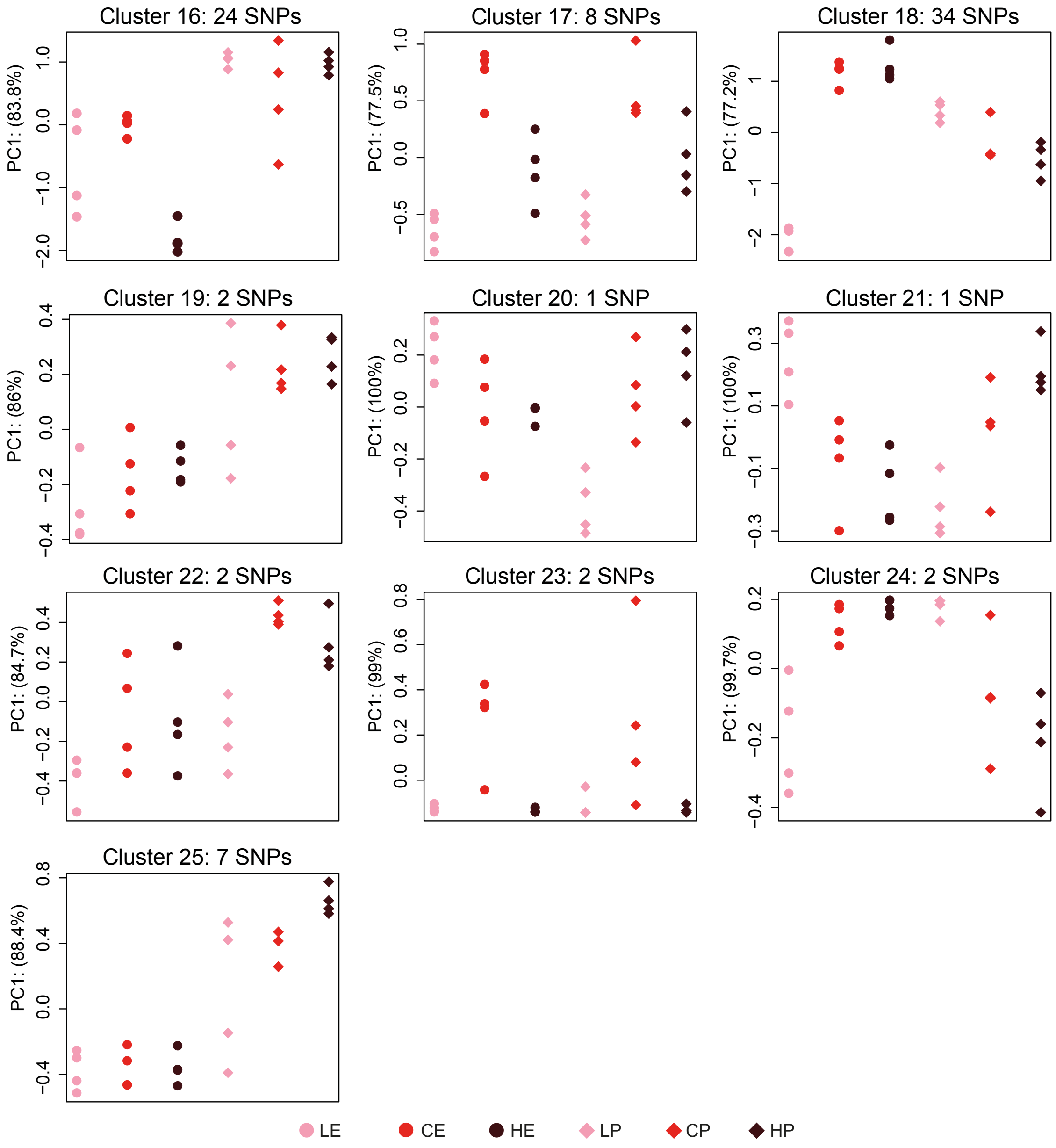

Figure 2: Clustering of SNPs with similar allele frequency differentiation patterns. The 25 clusters of SNPs that result from our clustering analysis are shown here. The location of the 24 populations on PC1 (y-axis) is indicated in the graphs. Populations with a high frequency of the major allele have a high value on the PC1 axis, whereas a low frequency of the major allele is indicated by lower PC1 values. These figures, therefore, give an average overview of the allele frequency differentiation in each cluster of SNPs.

Table 1: ANOVA on the PC1 values of the 25 clusters. The *P*-values of the ANOVA with Developmental diet, Age-at-reproduction and their interaction as factors are given. Effects were considered significant when $P < 0.002$ (Bonferroni correction: 0.05/25 clusters). Significant *P*-values are indicated in bold and pink shading indicates the most significant factor. In addition, the number of SNPs per cluster that is significant for each of the three factors in the GLM analysis is listed, to show the similarities in outcomes of the two analyses. The largest group of significant SNPs in the GLM analysis is indicated by pink shading as well.

Cluster	N SNPs	*P* Dev. Diet	*P* Age-at-Rep.	*P* Interaction	GLM Dev. Diet	GLM Age-at-Rep.	GLM Interaction
1	44	**<0.00001**	0.0123	**0.00056**	44	0	0
2	2	0.03478	0.39051	**<0.00001**	0	0	2
3	143	**7e-05**	**<0.00001**	**0.00177**	0	143	0
4	5	0.08068	**<0.00001**	0.21233	0	5	0
5	298	**0.00016**	**<0.00001**	0.07796	0	298	0
6	134	**<0.00001**	0.09724	**0.00042**	134	0	0
7	1112	**<0.00001**	**<0.00001**	**<0.00001**	828	215	191
8	1	0.7584	0.03262	**0.00024**	0	0	1
9	2	**<0.00001**	0.2781	0.00379	2	0	0
10	53	**0.00149**	**<0.00001**	0.00403	0	53	0
11	8	0.14774	**<0.00001**	0.30427	0	8	0
12	321	**<0.00001**	0.00203	0.44202	321	0	0
13	36	**<0.00001**	0.69587	0.9728	36	0	0
14	6	**<0.00001**	**0.00075**	0.50459	6	0	0
15	4	**2e-05**	0.6903	0.71865	4	0	0
16	24	0.0279	**<0.00001**	**0.00069**	0	23	1
17	8	**<0.00001**	0.84458	0.50692	8	0	0
18	34	**<0.00001**	0.09076	**<0.00001**	1	0	33
19	2	0.10305	**<0.00001**	0.9888	0	2	0
20	1	0.14833	0.05182	**6e-05**	0	0	1
21	1	0.6205	0.95953	**1e-05**	0	0	1
22	2	**0.00027**	**0.00014**	0.36475	1	1	0
23	2	**0.00054**	0.94888	0.99358	2	0	0
24	2	0.72001	0.14301	**1e-05**	0	0	2
25	7	0.04013	**<0.00001**	0.05435	0	7	0

Table 2: Post hoc tests. The *P*-values of the post hoc tests for (A) Age-at-reproduction (E-P) and Developmental diet (L-C, L-H, C-H) and (B) EE regime combination specific contrasts are shown for each of the 25 clusters (number of SNPs per cluster is indicated in brackets). Significant *P*-values are indicated by pink shading.

A

	E - P	L - C	C - H	L - H
**1 (44)**	0.012	0.045	1.0E-05	0
**2 (2)**	0.391	0.944	0.044	0.082
**4 (5)**	0	0.999	0.116	0.126
**3 (143)**	0	0.019	0.032	5.0E-05
**5 (298)**	0	2.1E-04	0.617	0.002
**6 (134)**	0.097	0	0.350	0
**7 (1112)**	0	0	0.575	0
**8 (1)**	0.033	0.830	0.993	0.767
**9 (2)**	0.278	0	0	0.654
**10 (53)**	0	0.038	0.258	0.001
**11 (8)**	0	0.996	0.217	0.188
**12 (321)**	0.002	0	0.009	0
**13 (36)**	0.696	1.0E-05	0.997	1.0E-05
**14 (6)**	0.001	2.8E-04	0.007	0
**15 (4)**	0.690	0.002	0.086	1.0E-05
**16 (24)**	0	0.998	0.045	0.052
**17 (8)**	0.845	0	8.0E-05	0.002
**18 (34)**	0.091	0	0.842	0
**19 (2)**	0	0.222	0.910	0.109
**20 (1)**	0.052	0.352	0.829	0.140
**21 (1)**	0.960	0.735	0.627	0.983
**22 (2)**	1.4E-04	3.5E-04	0.663	0.002
**23 (2)**	0.949	0.002	0.001	0.989
**24 (2)**	0.143	0.803	0.729	0.991
**25 (7)**	0	0.442	0.301	0.032

B

	LE- CE	LE- HE	CE- HE	LP- CP	LP- HP	CP- HP	LE- LP	CE- CP	HE- HP	LE- CP	LE- HP	1E- LP	CE- HP	HE- LP	HE- CP
**1 (44)**	0.301	0	1.0E-05	0.654	0.020	0.325	0.982	1.000	3.6E-04	0.284	0.005	0.678	0.306	0	1.0E-05
**2 (2)**	0.647	2.0E-05	3.1E-04	0.383	0.011	0.424	0.001	0.465	2.6E-04	0.033	0.690	0.013	1.000	0.501	0.015
**3 (143)**	0.100	2.0E-05	0.006	0.684	0.827	1.000	0	0	0.006	0	0	2.0E-05	0	0.076	0.004
**4 (5)**	0.999	1.000	0.993	1.000	0.122	0.199	2.9E-04	0.001	0.053	0.001	0.080	6.0E-04	0.151	1.9E-04	3.4E-04
**5 (298)**	0.001	0.013	0.685	0.439	0.370	1.000	0	0	0	0	0	0	0	0	0
**6 (134)**	0	0	0.543	1.4E-04	7.0E-05	1.000	0.075	0.245	0.017	0	0	0	0.383	0	0.009
**7 (1112)**	0	0	0.941	0.992	0.083	0.228	0	0.580	0.609	0	0	0.276	0.980	0.771	0.973
**8 (1)**	0.012	0.036	0.993	0.062	0.224	0.978	0.089	0.008	0.100	1.000	0.994	0.909	0.034	0.997	0.025
**9 (2)**	0	1.000	0	0.011	0.804	8.0E-04	0.642	0.015	1.000	4.4E-04	1.000	1.0E-05	0	0.641	4.4E-04
**10 (53)**	0.029	2.1E-04	0.218	0.999	1.000	1.000	0.099	0.000	0	0.191	0.172	8.0E-05	1.6E-04	0	0
**11 (8)**	0.998	0.994	1.000	1.000	0.356	0.231	0.004	0.019	1.9E-04	0.008	6.0E-05	0.010	1.4E-04	0.014	0.025
**12 (321)**	0	0	0.526	1.0E-05	0	0.064	0.098	0.895	0.220	0	0	4.0E-05	0.007	0	0.981
**13 (36)**	0.002	0.002	1.000	0.003	0.004	1.000	0.998	1.000	1.000	0.001	0.002	0.005	1.000	0.005	1.000
**14 (6)**	0.008	3.0E-05	0.112	0.073	0.001	0.308	0.044	0.291	0.620	9.0E-05	0	0.965	0.005	0.024	0.992
**15 (4)**	0.171	0.010	0.704	0.038	0.001	0.508	0.948	1.000	1.000	0.195	0.006	0.033	0.554	0.002	0.659
**16 (24)**	0.498	0.032	0.001	0.553	1.000	0.665	0.002	0.789	0	0.062	0.003	0.074	0.105	0	6.0E-05
**17 (8)**	1.0E-05	0.077	0.002	1.0E-04	0.076	0.047	0.990	0.952	0.989	3.0E-05	0.023	2.0E-05	0.008	0.221	0.014
**18 (34)**	0	0	0.989	0.076	0.004	0.723	0	6.0E-05	0	0	1.0E-05	0.028	0	0.008	2.0E-05
**19 (2)**	0.847	0.721	1.000	0.792	0.596	0.999	0.019	0.015	0.012	0.001	0.001	0.175	0.007	0.261	0.024
**20 (1)**	0.214	0.192	1.000	0.004	0.001	0.943	1.3E-04	0.975	0.552	0.580	0.972	0.019	0.592	0.022	0.964
**21 (1)**	0.019	0.003	0.932	0.149	0.002	0.263	0.001	0.914	0.006	0.131	0.998	0.608	0.044	0.983	0.418
**22 (2)**	0.204	0.262	1.000	0.003	0.033	0.882	0.540	0.015	0.094	1.0E-04	0.001	0.980	0.125	0.994	0.011
**23 (2)**	0.071	1.000	0.061	0.095	1.000	0.076	1.000	1.000	1.000	0.078	1.000	0.086	0.069	1.000	0.068
**24 (2)**	0.011	0.003	0.992	0.069	0.002	0.580	0.003	0.190	0.002	0.694	1.000	0.993	0.007	1.000	0.068
**25 (7)**	1.000	1.000	1.000	0.552	0.014	0.322	0.038	0.001	2.0E-05	0.001	2.0E-05	0.044	2.0E-05	0.048	0.001

Table 3: Inferred effect of selection regimes on the variation of PC1 for the 10 largest clusters.

Cluster	N SNPs	Inferred effect	Basis of inference
1	44	Interaction (HE)	HE regime significantly different from all other populations
2	2	Interaction	Significant effect for interaction only, and only LE *versus* HE is significant for the regime combination contrasts
3	143	Interaction	E is significantly different from P populations, also for the EE regime combination contrasts. Also, LE differs from HE
4	5	Age-at-reproduction	E significantly different from P populations
5	298	Age-at-reproduction	E significantly different from P, also for all EE regime combination contrasts for E *versus* P
6	134	Developmental diet (L)	L *versus* C and L *versus* H significant, and several EE regime combination contrasts with L samples
7	1112	Interaction (LE)	While E *versus* P, L *versus* C and L *versus* H are significant, all significant contrasts for the EE regime combinations are between LE and others
8	1	Interaction	Significant effect for interaction only, no contrasts
9	2	Developmental diet (C)	L *versus* C and C *versus* H significant, as well as several EE regime combination contrasts with C
10	53	Age-at-reproduction	E *versus* P and four EE regime combination contrasts between E *versus* P are significant
11	8	Age-at-reproduction	E significantly different from P populations
12	321	Developmental diet (L)	L *versus* C and L *versus* H significant, as well as several specific contrasts with L samples
13	36	Developmental diet (L)	L *versus* C and L *versus* H significant, and several regime combination contrasts with L populations
14	6	Developmental diet (L)	L *versus* H significant, and several EE regime combinations with L populations
15	4	Developmental diet	Significant effect of diet, but no contrast significant
16	24	Age-at-reproduction	E *versus* L and three EE regime combination contrasts of the E *versus* L are significant
17	8	Developmental diet (C)	0.25 *versus* 1 and 1 *versus* 2.5 significant, as well as several EE regime combination contrasts with 1 samples
18	34	Interaction	The main contrasts L *versus* C, and L *versus* H were significant, but the EE regime combination contrasts indicate an interaction. Among others, CE *versus* CL and HE *versus* HP are significant
19	2	Age-at-reproduction	E significantly different from P populations
20	1	Interaction	Significant effect for interaction only, no contrasts
21	1	Interaction	Significant effect for interaction only, no contrasts
22	2	Age-at-reproduction	Significant effect for age-at-reproduction. Only LE *versus* PL contrast is significant
23	2	Developmental diet	Significant effect for developmental diet only, no contrasts
24	2	Interaction	Significant effect for interaction only, no contrasts
25	7	Age-at-reproduction	E *versus* P and three EE regime combination contrasts between E *versus* P are significant

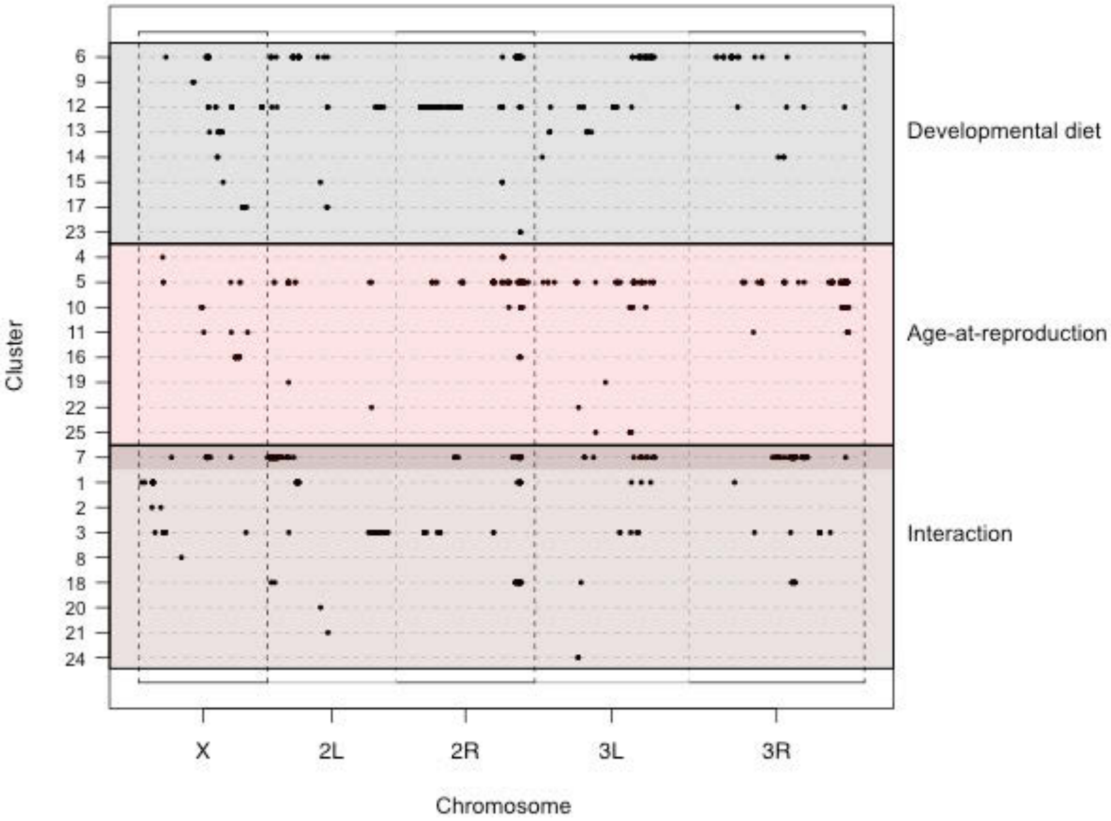

Figure 3: Location of SNPs within each cluster on the genome. The location of the SNPs located in each of the 25 clusters on the genome is shown. The clusters are sorted based on their inferred effect (see Table 3), i.e. an effect of Developmental diet only (top), of Age-at-reproduction only (middle), or an interaction of the two regimes (bottom; with cluster 7 containing "LE" loci indicated by darker shading). This figure shows that loci with similar allele differentiation patterns occur across distant locations of the genome.

Table 4: Overlap among subsets of candidate genes. The overlap of candidate genes associated with "Age-at-reproduction", "Developmental diet" and "Interaction". *P* was considered significant if < 0.0012 (Bonferroni correction: 0.05 / 43 intersections). *** $P_{Bonferroni}$ < 0.001, ** < $P_{Bonferroni}$ < 0.01, * 0.01 < $P_{Bonferroni}$ < 0.05.

**Intersections**	**Degree**	**Observed Overlap**	**Expected Overlap**	**Fold Enrichment**	***P***	
Age-at-reproduction & Diet	2	8	2.04	3.93	0.001	*
Age-at-reproduction & Interaction	2	12	3.18	3.78	8.52E-05	**
Developmental diet & Interaction	2	27	4.58	5.90	1.17E-13	***
Age-at-reproduction & Diet & Interaction	3	1	0.04	23.79	0.041	
Age-at-reproduction & LE	2	10	2.21	4.52	7.82E-05	**
Developmental diet & LE	2	19	3.19	5.96	5.12E-10	***
Age-at-reproduction & Diet & LE	3	1	0.03	34	0.029	

Figure 4: Overlaps between candidate genes associated with "Age-at-reproduction", "Developmental diet" and "Interaction".

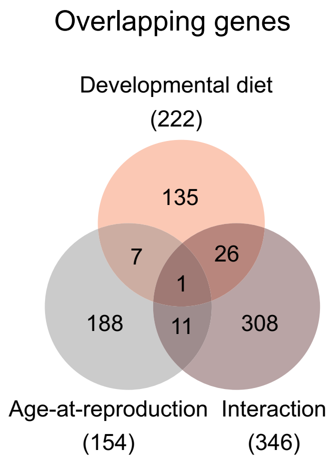

Supplementary Result S6: π and Tajima's *D*

To provide an overview of differences between the EE regimes at a global genomic scale, we made genome-wide estimations of genetic diversity (π) (Figure 1) and Tajima's *D* (Figure 2). The pattern of genetic diversity, with a decrease near the centromeres and the telomeres, is typical for natural populations (Orozco-TerWengel *et al*. 2012) and strongly dependent on genome-wide variation in recombination rate (Comeron *et al*. 2012). The average π per chromosome arm and of the entire genome was determined for each of the six EE regime combinations (Table 1). ANOVA of the average genome-wide π shows that there is no effect of the developmental diet regime ($F_{2,18}$ = 2.4, *P* = 0.12), but there is an effect of age-at-reproduction ($F_{1,18}$ = 11.2, *P* = 0.0036). In addition, there is an interaction of the two regimes ($F_{2,18}$ = 4.3, *P* = 0.029). Post-hoc Tukey-HSD contrasts indicate, however, that the significant variation in average π can be attributed solely to the LE populations, which had the lowest average π of all populations (significant post-hoc contrasts: (1) LE-CE: *P* = 0.041, (2) LE-CP: *P* = 0.034, (3) LE-LP: *P* = 0.006, (4) LE-HP: *P* = 0.005), whereas the other populations did not differ from each other.

This finding is especially relevant given that "E" and "P" populations have been subjected to different numbers of generations of selection at the time of sampling (respectively, 115 and 58 generations). This difference is inherent to our experimental design, which is based on imposing different generation times and has been applied by a number of other studies as well (Burke *et al*. 2010, Remolina *et al*. 2012, Carnes *et al*. 2015, Fabian *et al*. 2018), but may have implications for the interpretation of the results. One result of this difference in generations of selection may be that drift could have played a larger role for "E" compared to "P". Our results, however, indicate that "E" and "P" populations do not differ significantly from each other in terms of genetic diversity. Only "LE" populations differed from the others. This finding suggests that "E" populations did not differentiate more compared to "P" populations just as a result of drift. This can likely be attributed to the large population sizes and the relatively weak selection pressures applied in this EE study, which enables comparing "E" and "P" populations despite the difference in generations. Another concern associated with the difference in generations is that the "E" populations have had more time to adapt than the "P" populations. This raises questions about interpreting the observed interaction between larval diet and the "E" regime (but not the "P" regime). The results of the phenotyping experiments described in May *et al*. 2019 suggest that this may not be a problem as both "E" and "P" populations showed significant shifts in the two most important life history phenotypes, lifespan and development time, very quickly after selection had started. For example, for

both phenotypes effects of diet could be observed between "E" and "P" populations when assayed at 30-38 ("E") and 15-19 ("P") generations of selection, respectively. We sequenced the genomes of these lines considerably later, at 115 ("E") and 58 ("P") generations of selection. Also, we detected many loci associated with the developmental diet regime, which display similar patterns of differentiation among diets for both "E" and "P" populations, which suggests that the "P" populations have had sufficient time to adapt, despite their lower number of generations.

Although the overall genetic diversity is mostly similar between the six EE regime combinations, there are localized differences visible. To investigate these localized patterns of π, we performed ANOVA to analyze variation on the average π per 200 kB bins (665 bins in total). P was considered significant when < 0.000075 (Bonferroni: 0.05/665), which was observed for 35 bins (Figure 3, Table 2). Similarly, variation in Tajima's D among the six EE regime combinations was analyzed per 200 kB bins. A significant differentiation in Tajima's D was observed for 15 bins, of which 11 also had a significant differentiation of π (Table 3). Tukey-HSD post-hoc contrasts indicate that in almost all cases, LE populations had a significantly lower Tajima's D than the other populations (also see Figure 2). P was considered significant when < 0.00022 (Bonferroni: 0.05/(15*15)).

Tukey-HSD post-hoc contrasts indicate that in almost all cases, LE populations had a significantly lower π than all other populations (also see Figure 1). P was considered significant when < 0.000095 (Bonferroni: 0.05/(15*35)). Most notable are two large regions, close to the telomere of chromosome 2L (~2 Mb) and in the middle of chromosome 3R (~1 Mb), in which the LE populations had a lower genetic diversity as compared to all other populations (P < 0.0001 for all relevant contrasts). This may indicate that these regions have been under selection. Also, Tajima's D of the LE populations was significantly lower than in all other populations in these regions (P < 0.0001 for all relevant contrasts), which could indicate either a recent (hard) selective sweep or ongoing purifying selection. The observations of π and Tajima's D combined suggest that a selective sweep took place in these two genomic regions for the LE regime only. For the other EE regimes, few 200 kB regions in which π and Tajima's D differ from other regimes were observed, which suggests that the observed genetic variation among these likely occurs at a smaller genomic scale than what we analyzed.

References

Burke, M.K., Dunham, J.P., Shahrestani, P., Thornton, K.R., Rose, M.R., Long, A.D. (2010) Genome-wide analysis of a long-term evolution experiment with *Drosophila*. *Nature* 467:587-U111.

Carnes, M.U., Campbell, T., Huang, W., Butler, D.G., Carbone, M.A., Duncan, L.H., et al. (2015). The Genomic Basis of Postponed Senescence in *Drosophila melanogaster*. PLoS ONE 10:e0138569.

Comeron, J.M., Ratnappan, R., Bailin, S. (2012). The Many Landscapes of Recombination in *Drosophila melanogaster*. *PLoS Genet*. 8: e1002905.

Fabian, D.K., Garschall, K., Klepsatel, P., Santos-Matos, G., Sucena, E., Kapun, M., et al. (2018). Evolution of longevity improves immunity in *Drosophila*. Evol Lett. 2:567-579.

Orozco-TerWengel, P., Kapun, M., Nolte, V., Kofler, R., Flatt, T., Schlotterer, C. (2012). Adaptation of *Drosophila* to a novel laboratory environment reveals temporally heterogeneous trajectories of selected alleles. *Mol Ecol*. 21:4931-4941.

Remolina, S.C., Chang, P.L., Leips, J., Nuzhdin, S.V., Hughes, K.A. (2012). Genomic basis of aging and life-history evolution in *Drosophila melanogaster*. Evolution 66:3390-3403.

Table 1: average π per EE regime

	LE	CE	HE	LP	CP	HP
**2L**	0.00310	0.00361	0.00337	0.00331	0.00343	0.00336
**2R**	0.00235	0.00244	0.00240	0.00249	0.00248	0.00244
**3L**	0.00248	0.00261	0.00259	0.00267	0.00269	0.00268
**3R**	0.00213	0.00236	0.00233	0.00241	0.00240	0.00244
**X**	0.00167	0.00172	0.00170	0.00178	0.00174	0.00174
**All**	0.00234	0.00254	0.00247	0.00253	0.00254	0.00253

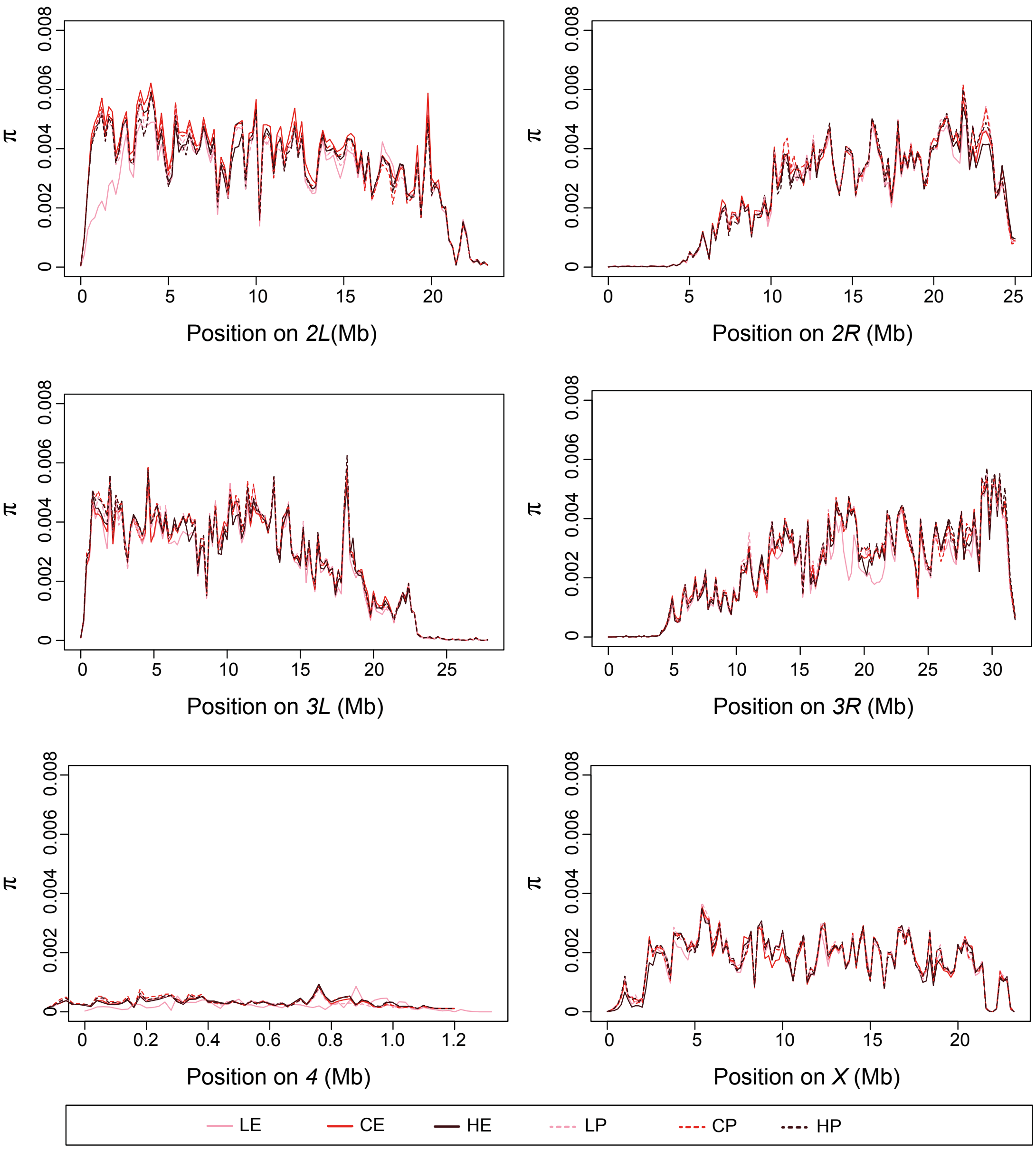

Figure 1: Average genetic diversity (*π*). Average genetic diversity (*π*) per experimental evolution (EE) regime along each chromosome as calculated for 200 kb, non-overlapping windows (20 kb for the 4th chromosome). EE regimes (Developmental diet x Age-at-reproduction): low (L), control (C) and high (H) diet, and early (E) versus postponed (P) reproduction.

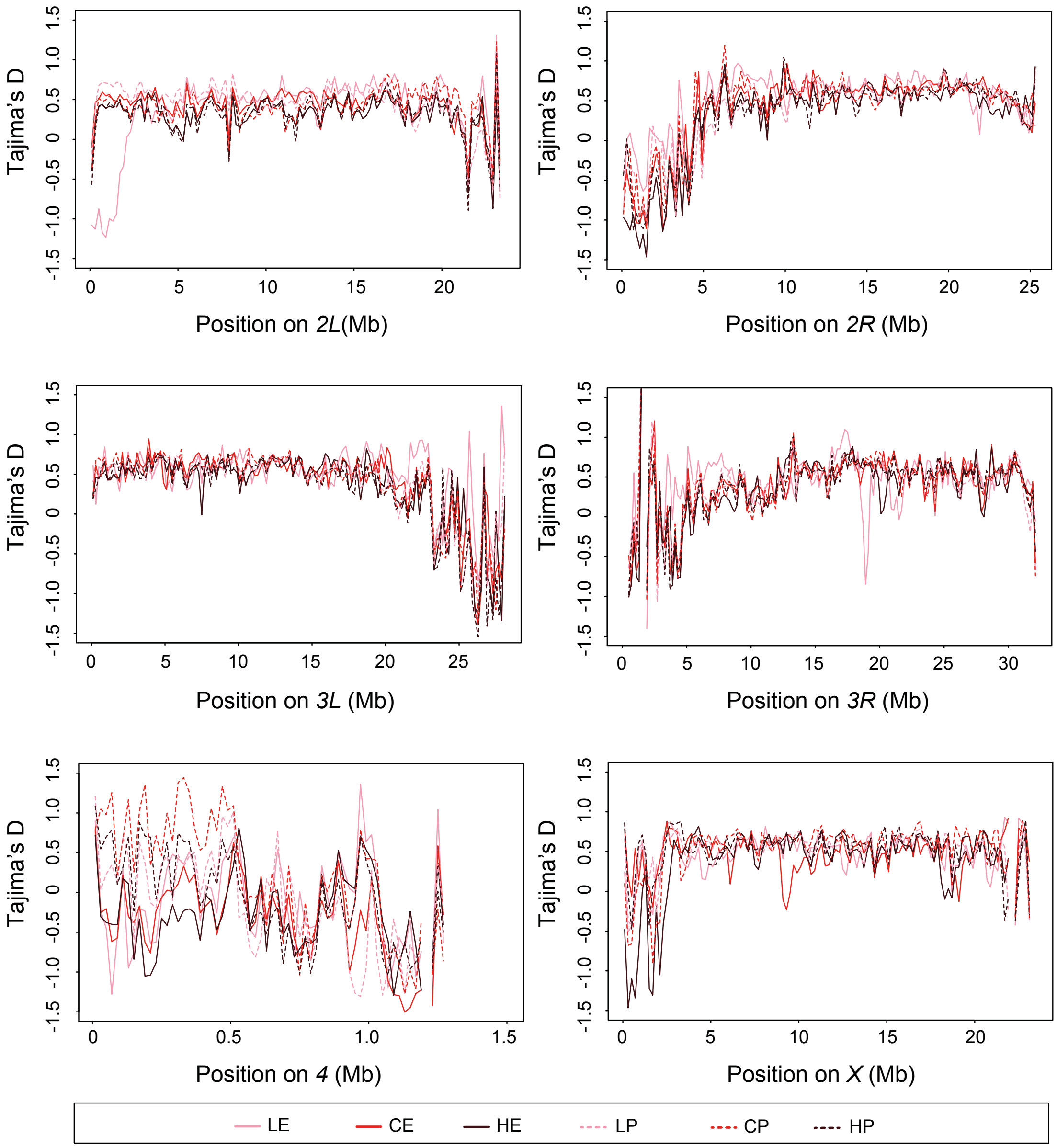

Figure 2: Tajima's *D*. Average Tajima's *D* per experimental evolution (EE) regime along each chromosome as calculated for 200 kb, non-overlapping windows (20 kb for the 4th chromosome). EE regimes (Developmental diet x Age-at-reproduction): low (L), control (C) and high (H) diet, and early (E) versus postponed (P) reproduction.

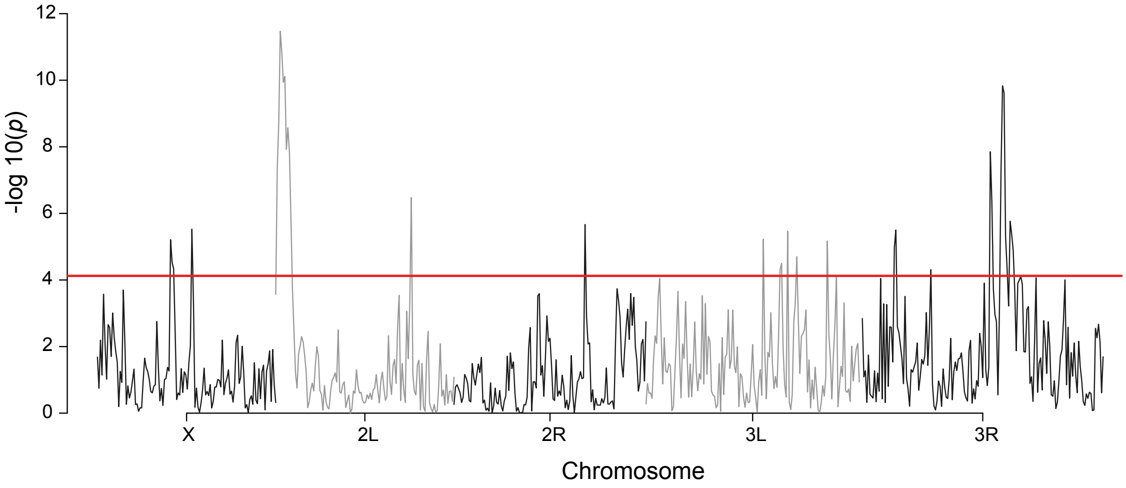

Figure 3: Variation in π across the genome. The variation in average *π* among the six EE regime combinations was assessed for non-overlapping 200 kB regions across the entire genome using ANOVA. The *P*-values are plotted on the y-axis and the horizontal red line indicates the threshold for significance (Bonferroni: 0.05/665 = 0.000075)

Table 2: ANOVA and Tukey-HSD post-hoc tests on the average π per 200 kB regions. The *P*-values of the ANOVA and the post-hoc contrasts of the 35 significant regions are shown. The ANOVA is considered significant when $P < 0.000075$ (Bonferroni: 0.05/665). The Tukey-HSD post-hoc contrasts are considered significant when $P < 0.000095$ (Bonferroni: 0.05/(15*35). Significant post-hoc contrasts are indicated by pink shading.

Position (Mb)	*ANOVA P*	*CE-CP*	*CE-LE*	*CE-LP*	*CE-HE*	*CE-HP*	*CL-LE*	*CL-LP*	*CP-HE*	*CP-HP*	*LE-LP*	*LE-HE*	*LE-HP*	*HE-LP*	*LP-HP*	*HE-HP*
**2L:0.2-0.4**	4.8E-08	0.986	1.3E-07	0.804	0.966	0.995	3.5E-07	0.988	1.000	1.000	9.7E-07	4.5E-07	2.8E-07	0.997	0.973	1.000
**2L:0.4-0.6**	1.8E-09	0.998	1.7E-08	0.999	1.000	0.991	9.6E-09	0.973	0.994	1.000	2.7E-08	2.0E-08	7.7E-09	1.000	0.939	0.980
**2L:0.6-0.8**	3.4E-12	0.595	1.2E-11	0.957	0.642	0.979	6.6E-11	0.966	1.000	0.937	2.8E-11	6.1E-11	2.5E-11	0.978	1.000	0.956
**2L:0.8-1.0**	1.4E-11	0.898	6.0E-11	0.991	0.882	0.943	1.9E-10	0.997	1.000	1.000	1.1E-10	2.0E-10	1.6E-10	0.995	1.000	1.000
**2L:1.0-1.2**	1.1E-10	0.983	4.3E-10	0.987	0.801	0.907	9.5E-10	1.000	0.991	0.999	9.1E-10	2.0E-09	1.4E-09	0.988	0.999	1.000
**2L:1.2-1.4**	7.7E-11	0.871	1.7E-10	0.528	0.671	0.313	6.0E-10	0.988	0.999	0.903	1.3E-09	9.5E-10	2.1E-09	1.000	0.998	0.986
**2L:1.4-1.6**	1.2E-08	0.631	1.6E-08	0.379	0.933	0.300	1.6E-07	0.998	0.987	0.989	3.2E-07	6.1E-08	4.2E-07	0.887	1.000	0.814
**2L:1.6-1.8**	2.7E-09	0.684	4.4E-09	0.247	0.865	0.706	3.4E-08	0.960	0.999	1.000	1.1E-07	2.0E-08	3.2E-08	0.846	0.952	1.000
**2L:1.8-2.0**	1.4E-08	0.337	1.5E-08	0.145	0.940	0.193	3.4E-07	0.994	0.840	0.999	8.0E-07	5.5E-08	6.0E-07	0.545	1.000	0.646
**2L:2.0-2.2**	1.5E-06	0.247	1.1E-06	0.311	0.961	0.393	7.6E-05	1.000	0.681	0.999	5.7E-05	4.7E-06	4.1E-05	0.766	1.000	0.849
**2L:17.8-18.0**	3.3E-07	6.8E-05	0.019	0.594	0.300	0.025	1.2E-07	0.002	0.006	0.094	0.001	2.0E-04	1.4E-05	0.994	0.437	0.753
**2R:17.2-17.4**	2.2E-06	0.004	0.108	0.005	0.319	0.003	1.5E-05	1.000	0.259	1.000	1.9E-05	0.001	1.0E-05	0.314	0.999	0.185
**3L:15.4-15.6**	6.0E-06	0.992	3.5E-05	0.992	0.992	0.869	1.1E-04	1.000	1.000	0.556	1.0E-04	1.1E-04	4.4E-06	1.000	0.562	0.560
**3L:17.6-17.8**	4.9E-05	0.050	0.325	0.017	0.982	0.020	0.001	0.995	0.175	0.997	2.1E-04	0.106	2.4E-04	0.068	1.000	0.077
**3L:17.8-18.0**	3.2E-05	0.270	5.6E-05	0.458	0.997	0.400	0.006	0.999	0.125	1.000	0.003	2.3E-05	0.003	0.239	1.000	0.201
**3L:18.6-18.8**	3.4E-06	0.972	0.006	0.879	0.002	0.729	0.030	0.999	0.000	0.986	0.059	7.7E-07	0.105	2.4E-04	1.000	1.3E-04
**3L:19.8-20.0**	2.0E-05	0.006	1.1E-05	0.002	0.093	0.448	0.055	0.992	0.749	0.229	0.158	0.003	4.2E-04	0.419	0.085	0.919
**3L:23.8-24.0**	6.8E-06	0.541	0.001	0.915	0.998	0.118	0.020	0.123	0.790	0.004	7.3E-05	0.001	2.9E-06	0.714	0.528	0.052
**3R:4.6-4.8**	1.1E-05	0.001	5.0E-05	0.331	2.2E-04	0.735	0.683	0.078	0.975	0.019	0.004	0.973	0.001	0.018	0.978	0.004
**3R:4.8-5.0**	3.2E-06	0.999	6.6E-05	0.953	0.213	0.291	1.3E-04	0.995	0.358	0.167	3.7E-04	0.009	1.2E-06	0.651	0.065	0.003
**3R:9.4-9.6**	4.9E-05	0.554	0.005	0.439	1.000	0.460	1.5E-04	1.000	0.430	1.000	9.9E-05	0.008	1.1E-04	0.328	1.000	0.346
**3R:17.2-17.4**	1.4E-08	0.556	3.7E-06	0.005	0.999	0.857	2.2E-07	0.145	0.376	0.993	5.1E-09	6.5E-06	5.2E-07	0.002	0.052	0.690
**3R:17.4-17.6**	4.6E-07	0.591	1.6E-04	0.124	0.262	0.291	7.0E-06	0.887	0.012	0.993	1.1E-06	0.018	2.6E-06	0.001	0.995	0.004

**3R:18.6-18.8**	5.6E-08	0.885	1.0E-06	0.997	0.999	0.931	1.8E-07	0.988	0.976	1.000	4.8E-07	5.8E-07	2.2E-07	1.000	0.996	0.990
**3R:18.8-19.0**	1.5E-10	0.997	1.9E-09	0.980	0.891	0.874	1.1E-09	1.000	0.650	0.987	8.2E-10	7.5E-09	5.1E-10	0.517	0.998	0.301
**3R:19.0-19.2**	2.4E-10	0.996	5.1E-09	0.852	0.992	0.818	2.7E-09	0.984	1.000	0.974	1.2E-09	2.4E-09	1.1E-09	0.991	1.000	0.985
**3R:19.2-19.4**	5.2E-06	0.994	2.2E-05	0.996	1.000	0.986	6.1E-05	0.906	0.999	1.000	8.9E-06	2.9E-05	7.5E-05	0.987	0.867	0.996
**3R:19.4-19.6**	4.6E-05	0.557	3.8E-05	0.902	0.929	0.797	0.001	0.984	0.974	0.998	2.9E-04	2.5E-04	4.8E-04	1.000	1.000	0.999
**3R:19.8-20.0**	1.7E-06	0.513	0.001	0.139	0.849	0.113	2.3E-05	0.947	0.082	0.916	4.5E-06	0.009	3.7E-06	0.014	1.000	0.011
**3R:20.0-20.2**	4.2E-06	0.216	0.053	0.118	0.015	0.514	3.7E-04	0.999	1.1E-04	0.988	1.8E-04	0.989	0.001	5.3E-05	0.923	3.7E-04
**3R:20.2-20.4**	1.3E-05	0.954	0.008	0.998	0.003	0.665	0.001	0.998	0.001	0.984	0.003	0.997	3.5E-04	0.001	0.882	1.4E-04
**X:9.6-9.8**	6.1E-06	0.007	1.5E-05	4.6E-05	4.6E-05	3.1E-05	0.066	0.197	0.197	0.138	0.989	0.989	0.998	1.000	1.000	1.000
**X:9.8-10.0**	2.9E-05	0.004	1.4E-04	2.0E-04	4.7E-05	1.3E-04	0.594	0.706	0.305	0.575	1.000	0.994	1.000	0.977	1.000	0.996
**X:10.0-10.2**	4.6E-05	0.001	1.5E-04	0.001	1.4E-04	7.2E-05	0.978	1.000	0.975	0.880	0.923	1.000	0.999	0.915	0.758	0.999
**X:12.4-12.6**	3.0E-06	0.419	0.002	0.215	0.033	0.302	4.7E-05	0.997	0.693	1.000	2.0E-05	3.2E-06	3.0E-05	0.910	1.000	0.820

Table 3: ANOVA and Tukey-HSD post-hoc tests on the average Tajima's *D* per 200 kB regions. The *P*-values of the ANOVA and the post-hoc contrasts of the 15 significant regions are shown. The ANOVA is considered significant when $P < 0.000075$ (Bonferroni: 0.05/665). The Tukey-HSD post-hoc contrasts are considered significant when $P < 0.00022$ (Bonferroni: 0.05/(15*15). Regions for which π has differentiated as well are indicated with an asterisk. Significant post-hoc contrasts are indicated by pink shading.

Position (Mb)	*ANOVA P*	*CE-CP*	*CE-LE*	*CE-LP*	*CE-HE*	*CE-HP*	*CP-LE*	*CP-LP*	*CP-HE*	*CP-HP*	*LE-LP*	*LE-HE*	*LE-HP*	*HE-LP*	*LP-HP*	*HE-HP*
*** 2L:0.2-0.4**	2.2E-07	0.789	3.6E-07	1.000	0.715	0.336	3.1E-06	0.910	1.000	0.963	5.6E-07	3.9E-06	1.3E-05	0.858	0.485	0.983
*** 2L:0.4-0.6**	8.9E-09	1.000	8.0E-08	0.797	0.984	0.978	7.0E-08	0.840	0.973	0.964	1.3E-08	2.2E-07	2.4E-07	0.418	0.390	1.000
*** 2L:0.6-0.8**	3.1E-10	0.948	1.7E-09	0.934	0.863	0.818	5.2E-09	0.488	1.000	0.999	5.6E-10	7.3E-09	8.4E-09	0.350	0.304	1.000
*** 2L:0.8-1.0**	1.3E-09	0.999	8.8E-09	0.892	0.893	0.902	1.4E-08	0.727	0.978	0.981	2.2E-09	3.9E-08	3.7E-08	0.325	0.337	1.000
*** 2L:1.0-1.2**	8.2E-10	1.000	5.9E-09	0.885	0.863	0.965	7.5E-09	0.806	0.927	0.988	1.5E-09	2.8E-08	1.7E-08	0.283	0.451	0.999
*** 2L:1.2-1.4**	2.3E-11	1.000	2.4E-10	0.618	0.924	0.966	2.2E-10	0.673	0.892	0.946	4.3E-11	7.4E-10	6.0E-10	0.162	0.217	1.000
*** 2L:1.4-1.6**	4.1E-08	0.997	4.5E-07	0.645	1.000	0.995	9.7E-07	0.374	0.997	1.000	4.2E-08	4.5E-07	1.0E-06	0.643	0.353	0.995
*** 2L:1.6-1.8**	4.3E-08	0.965	3.3E-07	0.686	0.924	0.992	1.2E-06	0.257	1.000	1.000	3.5E-08	1.6E-06	8.4E-07	0.197	0.360	0.999
*** 2L:1.8-2.0**	1.2E-06	0.829	4.5E-06	0.920	0.777	0.775	4.3E-05	0.294	1.000	1.000	8.4E-07	5.2E-05	5.3E-05	0.251	0.249	1.000
**2R:23.2-23.4**	4.7E-05	0.250	1.000	0.051	0.031	0.301	0.392	0.946	2.6E-04	1.000	0.093	0.016	0.457	4.4E-05	0.912	3.3E-04
**3L:22.2-22.4**	4.8E-05	0.947	7.7E-04	0.203	0.641	1.000	0.005	0.646	0.200	0.889	0.111	3.4E-05	5.2E-04	0.010	0.147	0.749
**3R:3.6-3.8**	3.0E-06	1.000	7.2E-05	0.137	0.006	0.868	6.1E-05	0.118	0.005	0.902	0.017	0.332	8.6E-06	0.609	0.015	5.3E-04
**3R:17.0-17.2**	2.2E-05	1.000	1.7E-04	0.407	0.586	0.867	1.0E-04	0.282	0.435	0.951	0.010	0.005	1.9E-05	1.000	0.062	0.112
*** 3R:18.8-19.0**	1.2E-09	1.000	7.0E-09	0.997	0.996	0.998	4.8E-09	0.972	0.969	0.981	1.3E-08	1.4E-08	1.2E-08	1.000	1.000	1.00
*** 3R:19.0-19.2**	3.2E-09	0.968	4.4E-08	0.997	0.997	0.999	1.5E-08	0.999	0.999	0.998	2.3E-08	2.3E-08	2.7E-08	1.000	1.000	1.00

Supplementary Result S7: Low-recombining regions

Compared to regions with high recombination rates, low-recombining regions that are under selection may contain relatively higher number of hitchhiking loci, linked to the actual locus/loci under selection. Such linked loci may hamper or bias the interpretation of our functional enrichment analyses or comparative studies. Recombination rates are highly variable throughout the genome and display intra-specific variation (Comeron *et al*. 2012), which makes it difficult to estimate the effect of recombination at small genomic scales in our EE populations. However, there is a general pattern of low-/non-recombining regions near the centromeres and the telomeres in the *D. melanogaster* genome. We set out to test if candidate loci within these low-/non-recombining regions provide any bias to our functional enrichment and comparative analyses, by excluding SNPs from these region and re-running (1) the GO enrichment analyses using Gowinda, (2) the comparative analyses with the GenAge database, and (3) overlap analyses with the three other longevity "E&R" studies (i.e. the non-/low-recombining regions were excluded from these three other studies as well). All analyses were performed as described in the Supplementary Methods file.

We exclude SNPs from non-/low-recombining regions as described by Comeron et al. (2012); i.e. only SNPs from the following regions were included (coordinates converted to *D. melogaster* genome release 6):
X:2’405’967..20’928’973
2L:500’000..17’400’000
2R:9’312’495..24’912’477
3L:700’000..19’906’900
3R:13’874’278..31’074’278

As a result ~35% of the genome is masked (Figure 1) and 324 of the 2252 candidate SNPs (14.4%) are excluded: 110 of 513 (21.4%) "developmental diet" loci, 28 of 399 (7.0%) "postponed reproduction" loci, and 186 of 1340 (13.9%) "interaction" loci (of which 75 are "LE" loci) (Supplementary Table S1).

Comparative analyses yielded mostly comparable results for the filtered dataset compared to the full dataset. There is no significant overlap of our candidate genes with the GenAge databank (Table 1), but there is a significant overlap of our candidates with those of the three other longevity E&R studies. Our "age-at-reproduction" loci have a significant overlap with the Carnes *et al*. (2015) and Fabian *et al*. (2018) datasets, while our "LE" loci have a significant overlap with the Remolina *et al*. (2012) dataset (Table 2). Also, the Carnes *et al*. (2015) candidates have a significant overlap with our "development diet" loci, and (in contrast to the analysis on the complete

dataset) with the "interaction"/"LE" loci. This likely reflects the differentiation in development time that was observed among E and P populations for this study. In conclusion, although the results when removing loci from non-/low-recombining regions show some subtle differences, the main patterns and conclusions remain valid. From this we conclude that low-recombining regions did not bias our analyses and interpretations.

References

Carnes, M.U., Campbell, T., Huang, W., Butler, D.G., Carbone, M.A., Duncan, L.H., et al. (2015). The Genomic Basis of Postponed Senescence in *Drosophila melanogaster*. *PLoS ONE* 10:e0138569.

Comeron, J. M., Ratnappan, R., Bailin, S. (2012) The many landscapes of recombination in *Drosophila melanogaster*. *PLoS Genet*. 8:e1002905.

Fabian, D.K., Garschall, K., Klepsatel, P., Santos-Matos, G., Sucena, E., Kapun, M., et al. (2018). Evolution of longevity improves immunity in *Drosophila*. *Evol Lett*. 2:567-579.

Remolina, S.C., Chang, P.L., Leips, J., Nuzhdin, S.V., Hughes, K.A. (2012). Genomic basis of aging and life-history evolution in *Drosophila melanogaster*. *Evolution* 66:3390-3403.

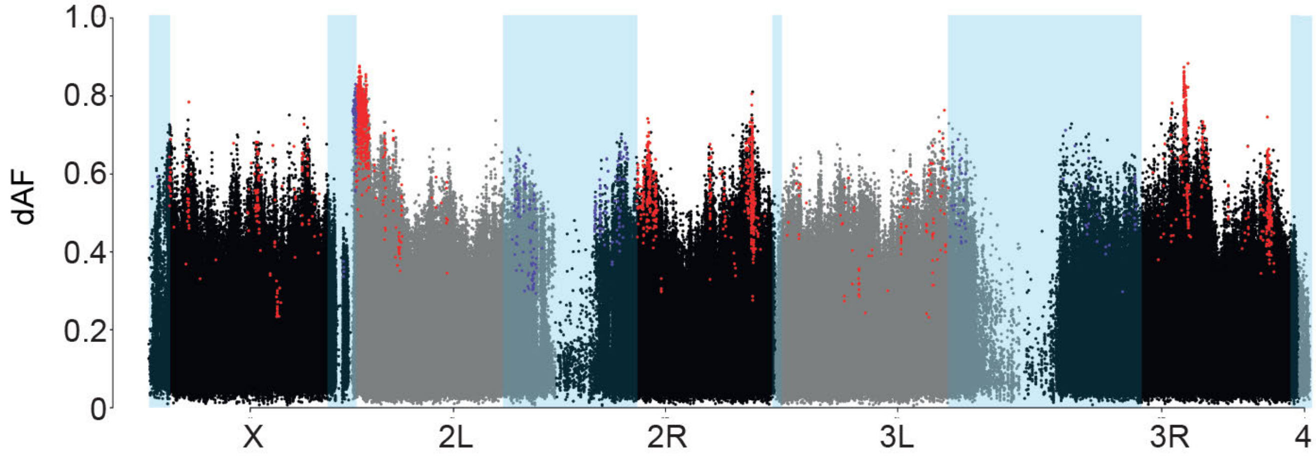

Figure 1: Excluded low-recombining regions. The plots show the maximum pairwise mean SNP allele differentiation among the six EE regime combinations, thereby indicating a combination of main effects and interaction the two regimes. All 2252 significant loci are highlighted. The low-recombining regions that are excluded are indicated with blue shading. GO enrichment analyses on the filtered dataset provided largely similar results as the full dataset. There were no significant GO terms detected for all candidate SNPs combined, for the E-P loci and the interaction loci. However, we did detect three significant GO terms for the developmental diet loci in this case. Five genes belong to these terms (GO: 0042445 "hormone metabolic process", GO:008202 "steroid metabolic process", and GO0010817 "regulation of hormone levels"; FDR = 0.036 for each term, which suggest changes in hormonal signalling during development associated with adaptation to larval diet. Although not significant after FDR correction previously, these three GO terms were also the most significant terms in our analysis of the full dataset.

Table 1: Overlap with the GenAge database. The overlap of our significant genes, or subsets of genes as determined by the clustering analysis (low-recombining regions removed), with "aging" genes from the GenAge database was determined. The overlap with "aging" genes that were confirmed in Drosophila ("Drosophila only"), as well as orthologs of known "aging" genes in other model organisms ("Drosophila + orthologs") was determined. In addition, the overlap of the GenAge gene lists with the three other longevity E&R datasets (Carnes *et al*. 2015; Fabian *et al*. 2018; Remolina *et al*. 2012) was assessed. The total number of genes per dataset is given in brackets. *P* was considered significant if < 0.0012 (Bonferroni correction: 0.05 / 43 intersections). O = observed overlap, E = expected overlap, FC = fold change.

	GenAge - *Drosophila* only (188)				GenAge - *Drosophila* + orthologs (579)			
	O	E	FC	*P*	O	E	FC	*P*
**All significant genes** (508)								
**Developmental diet** (160)	3	2.36	1.27	0.421	6	7.26	0.83	0.740
**Age-at-reproduction** (137)	0	2.02	0.00	n.a.	2	6.22	0.32	0.987
**Interaction** (249)	6	3.67	1.63	0.162	13	11.30	1.15	0.342
**"LE" genes only** (204)	4	3.01	1.33	0.354	11	9.26	1.19	0.323
**Carnes *et al*** (1215)	16	17.91	0.89	0.720	45	55.16	0.82	0.942
**Fabian *et al*** (725)	11	10.69	1.03	0.505	29	32.92	0.88	0.789
**Remolina *et al*** (941)	16	13.87	1.15	0.313	36	42.72	0.84	0.882

Table 2: Overlap among longevity E&R studies. The overlap of significant genes from our study (low-recombining regions removed) with the aging E&R datasets (Carnes *et al.* 2015; Fabian *et al*. 2018; Remolina *et al*. 2012) was determined. We assessed the 2, 3, and 4 degree intersections of our "longevity" loci (i.e. genes with a response to selection for postponed reproduction) with the three other datasets, as well as the 2 degree intersections of our "developmental diet", "interaction" and "LE" genes with these datasets. *P* was considered significant if < 0.0012 (Bonferroni correction: 0.05 / 43 intersections); *** PBonferroni < 0.001, ** < PBonferroni < 0.01, * 0.01 < PBonferroni < 0.05. O = observed overlap, E = expected overlap, FC = fold change.

Intersections	Degree	O	E	FC	*P*	
**Overlap with "Age-at-reproduction genes" (longevity candidates)**						
This study & Remolina *et al*	2	5	10.11	0.49	0.977	
This study & Carnes *et al*	2	33	13.05	2.53	4.17E-07	***
This study & Fabian *et al*	2	26	7.79	3.34	4.65E-08	***
Fabian *et al* & Remolina *et al*	2	64	53.50	1.20	0.074	
Carnes *et al* & Remolina *et al*	2	199	89.65	2.22	3.05E-29	***
Fabian *et al* & Carnes *et al*	2	112	69.07	1.62	1.23E-07	***
This study & Carnes *et al* & Remolina *et al*	3	4	0.96	4.15	0.016	
This study & Fabian *et al* & Remolina *et al*	3	0	0.57	0	n.a.	
This study & Fabian *et al* & Carnes *et al*	3	7	0.74	9.43	1.10E-05	***
Fabian *et al* & Carnes *et al* & Remolina *et al*	3	19	5.10	3.73	1.38E-06	***
This study & Fabian *et al* & Carnes *et al* & Remolina *et al*	4	0	0.05	0	n.a.	
**Overlap with "Developmental diet" genes**						
This study & Remolina *et al*	2	13	11.81	1.10	0.401	
This study & Carnes *et al*	2	42	15.24	2.76	7.01E-10	***
This study & Fabian *et al*	2	12	9.10	1.32	0.200	
**Overlap with "Interaction" genes**						
This study & Remolina *et al*	2	35	18.37	1.90	0.0002	**
This study & Carnes *et al*	2	45	23.72	1.90	1.86E-05	***
This study & Fabian *et al*	2	25	14.16	1.77	0.0041	
**Overlap with "LE" genes**						
This study & Remolina *et al*	2	34	15.05	2.26	5.77E-06	***
This study & Carnes *et al*	2	34	19.44	1.75	0.001	*
This study & Fabian *et al*	2	16	11.60	1.38	0.120	

Supplementary Result S8: Cosmopolitan inversions

The presence of 7 cosmopolitan inversions that occur in *D. melanogaster* was assessed by screening our dataset for a set of SNP markers that are specific to the standard arrangement or the inversion. First, it was tested if any of the markers for a given inversion were polymorphic according to our criteria for SNPs. If part of the markers was polymorphic, indicating the presence of the inversion, we calculated the average frequency of the inversion-specific allele per population of all markers (including the ones that were not polymorphic, in order to not overestimate the frequency of the inversion). The inversions *In(2L)t*, *In(3R)C* and *In(3R)Mo* occur in low frequencies in the EE populations (2-6% on average) (Table 1). There is, however, no significant differentiation in allele frequency for any the marker positions of the three inversions among populations (results not shown), suggesting that there is no variation in inversions frequencies among the EE regimes and, therefore, that these inversions do not play a role in the adaptation to these regimes. There is no indication for the presence of *In(2R)Ns*, *In(3L)P*, *In(3R)K* and *In(3R)Payne*.

Table 1: The occurrence and frequency of inversion polymorphisms in the EE populations

Inversion	Arrangement	LE (1-4)				CE (1-4)				HE (1-4)			
**In(2L)t**	Both	0.02	0.04	0.01	0.00	0.14	0.04	0.23	0.04	0.11	0.03	0.03	0.05
**In(2R)Ns**	Standard	n.a.				n.a.				n.a.			
**In(3L)P**	Standard	n.a.				n.a.				n.a.			
**In(3R)C**	Both	0.01	0.01	0.01	0.00	0.01	0.01	0.01	0.01	0.01	0.01	0.01	0.01
**In(3R)K**	Standard	n.a.				n.a.				n.a.			
**In(3R)Mo**	Both	0.02	0.02	0.01	0.01	0.02	0.02	0.02	0.02	0.03	0.02	0.02	0.02
**In(3R)Payne**	Standard	n.a.				n.a.				n.a.			

Inversion	Arrangement	LP (1-4)				CP (1-4)				HP (1-4)			
**In(2L)t**	Both	0.00	0.00	0.00	0.11	0.04	0.06	0.10	0.10	0.08	0.06	0.08	0.07
**In(2R)Ns**	Standard	n.a.				n.a.				n.a.			
**In(3L)P**	Standard	n.a.				n.a.				n.a.			
**In(3R)C**	Both	0.01	0.01	0.02	0.01	0.01	0.01	0.03	0.01	0.03	0.05	0.02	0.04
**In(3R)K**	Standard	n.a.				n.a.				n.a.			
**In(3R)Mo**	Both	0.03	0.03	0.02	0.02	0.01	0.02	0.02	0.02	0.01	0.01	0.02	0.02
**In(3R)Payne**	Standard	n.a.				n.a.				n.a.			
